# Multi-parametric MRI-based radiomics for preoperative prediction of multiple biological characteristics in endometrial cancer

**DOI:** 10.3389/fonc.2023.1280022

**Published:** 2023-12-15

**Authors:** Changjun Ma, Ying Zhao, Qingling Song, Xing Meng, Qihao Xu, Shifeng Tian, Lihua Chen, Nan Wang, Qingwei Song, Liangjie Lin, Jiazheng Wang, Ailian Liu

**Affiliations:** ^1^ Department of Radiology, First Affiliated Hospital, Dalian Medical University, Dalian, China; ^2^ Medical Imaging Articial Intelligence Engineering Technology Research Center, Dalian, China; ^3^ Dalian Women and Children’s Medical Group, Dalian, China; ^4^ Clinical & Technical Support, Philips Healthcare, Beijing, China

**Keywords:** endometrial cancer, microsatellite instability, human epidermal growth factor receptor-2, deep myometrium invasion, lympho-vascular space invasion, radiomics

## Abstract

**Purpose:**

To develop and validate multi-parametric MRI (MP-MRI)-based radiomics models for the prediction of biological characteristics in endometrial cancer (EC).

**Methods:**

A total of 292 patients with EC were divided into LVSI (*n* = 208), DMI (*n* = 292), MSI (*n* = 95), and Her-2 (*n* = 198) subsets. Total 2316 radiomics features were extracted from MP-MRI (T_2_WI, DWI, and ADC) images, and clinical factors (age, FIGO stage, differentiation degree, pathological type, menopausal state, and irregular vaginal bleeding) were included. Intra-class correlation coefficient (ICC), spearman’s rank correlation test, univariate logistic regression, and least absolute shrinkage and selection operator (LASSO) were used to select radiomics features; univariate and multivariate logistic regression were used to identify clinical independent risk factors. Five classifiers were applied (logistic regression, random forest, decision tree, K-nearest neighbor, and Bayes) to construct radiomics models for predicting biological characteristics. The clinical model was built based on the clinical independent risk factors. The combined model incorporating the radiomics score (radscore) and the clinical independent risk factors was constructed. The model was evaluated by ROC curve, calibration curve (H-L test), and decision curve analysis (DCA).

**Results:**

In the training cohort, the RF radiomics model performed best among the five classifiers for the three subsets (MSI, LVSI, and DMI) according to AUC values (AUC_MSI_: 0.844; AUC_LVSI_: 0.952; AUC_DMI_: 0.840) except for Her-2 subset (Decision tree: AUC=0.714), and the combined model had higher AUC than the clinical model in each subset (MSI: AUC_combined_ =0.907, AUC_clinical_ =0.755; LVSI: AUC_combined_ =0.959, AUC_clinical_ =0.835; DMI: AUC_combined_ = 0.883, AUC_clinical_ =0.796; Her-2: AUC_combined_ =0.812, AUC_clinical_ =0.717; all *P*<0.05). Nevertheless, in the validation cohort, significant differences between the two models (combined vs. clinical model) were found only in the DMI and LVSI subsets (DMI: AUC_combined_ =0.803, AUC_clinical_ =0.698; LVSI: AUC_combined_ =0.926, AUC_clinical_ =0.796; all *P*<0.05).

**Conclusion:**

The radiomics analysis based on MP-MRI and clinical independent risk factors can potentially predict multiple biological features of EC, including DMI, LVSI, MSI, and Her-2, and provide valuable guidance for clinical decision-making.

## Introduction

Endometrial cancer (EC) is the sixth most common cancer in women and the most common malignant tumor of the female reproductive system (1, 2). Over the last two decades, its incidence has been increasing, particularly in young women (3). The main clinical symptoms of EC include vaginal bleeding after menopause, bleeding during and between menstrual periods, and pelvic pain; other important risk factors include obesity, no history of pregnancy, and longer menstruation (4). Different biological characteristics of EC may lead to different treatment efficacies and prognoses.

The expression of the human epidermal growth factor receptor-2 (Her-2) gene in patients with EC was found to be associated with tumor tissue differentiation, deep myometrium invasion (DMI), lymph node metastasis (LNM), and lympho-vascular space invasion (LVSI), which affects clinical treatment decisions (5). Microsatellite instability (MSI) is caused by the defection of mismatch repair (MMR) protein (6), which leads to uncorrectable mismatch bases and the accumulation of gene mutations, and, ultimately, a malignant cell transformation (7). In EC patients, the MSI status prediction has been useful for Lynch syndrome monitoring and disease progress estimation (8). Moreover, DMI is a key factor that determines the surgical approach, affects the prognosis of patients, and is closely related to LNM (9). LVSI is defined as the presence of cancer cells within lymphatics and/or blood vessels and has an essential role in the spread of tumor cells. LVSI-positive EC has a significantly worse prognosis, and LVSI-positive stage I EC patients are at risk for disease recurrence. Since LVSI is associated with LNM, preoperative assessment of LVSI status may aid treatment decisions (10, 11). Thus, identifying the biological characteristics of EC may contribute to the tailored treatment and increase survival rates in EC patients.

Histopathology and molecular sequencing are the main methods for determining biological characteristics (12). However, the dynamic process of tumour genesis and progression exhibits spatial and temporal heterogeneity, which may have a significant impact on tumor metastasis and its response to treatment (13). Invasive sampling methods such as puncture biopsy are risky, invasive and potentially complicating, all of which limit their application in real-time monitoring of disease progression and tumour biology (12, 14). At the same time, the puncture biopsy sample size does not allow for a comprehensive assessment of the biology of the entire tumour region, thus ignoring some of the heterogeneity of the tumor, also limiting the application of these methods. Magnetic resonance imaging (MRI) is a comprehensive, non-invasive, and repeatable assessment of tumor biology that can be used to monitor tumor response to therapy almost in real-time. In particular, multi-parametric MRI (MP-MRI) can reveal phenotypic differences in tumors to a certain extent by displaying a signal intensity and/or enhancement features.

Radiomics is a quantitative process that can simultaneously provide data on tissue composition and spatial tumor heterogeneity by analyzing a large number of radiomics features from medical images to generate imaging biomarkers for evidence-based clinical decision-making. This approach has the advantages of high data dimension and the ability to perform quantifiable analysis and convert image data into high-resolution spatial features, thereby realizing lesion feature extraction and model building. Currently, radiomics is applied in tumor segmentation (15), preoperative evaluation of DMI (16), LNM (17), and LVSI (18), prediction of immune-histochemical indicators (19), efficacy evaluation, and prognosis prediction (20) in EC. Recently, a systematic review and meta-analysis (21)suggested that pre-operative MRI-radiomics analyses in patients with EC is a good predictor of tumor grading, DMI, LVSI, and LNM. At present, there are few comprehensive reports of radiomics studies involving the prediction of biological features of EC. More importantly, previous related studies between radiomics and tumor biological features mainly focused on the evaluation of a single indicator. However, the occurrence and development of tumors and the treatment response are affected by multiple biological characteristics of tumors, so comprehensive analysis and evaluation of multiple biological characteristics are urgently required.

In this study, we aimed to develop and evaluate MP-MRI-based radiomics models as a non-invasive diagnostic method to predict several biological characteristics of EC. To further explore whether the construction of combined models integrating clinical independent risk factors and radscore can improve the accuracy of decision-making in the clinical treatment of EC.

## Materials and methods

### Patients and data collection

The ethics committee approved this retrospective study and waived the requirement for informed consent. Our research was a case-control study. A total of 366 patients confirmed with EC by postoperative pathology between January 2012 and June 2022 were retrospectively analyzed. Inclusion criteria were the following: (1) postoperative pathologically confirmed EC; (2) patients underwent pelvic MRI examination within two weeks before surgery, including T2-weighted imaging (T_2_WI), and diffusion-weighted imaging (DWI); (3) patients without other malignancies in the reproductive system. Exclusion criteria were as follows: (1) no history of surgery (*n* = 12); (2) the maximum diameter of the tumor was < 1 cm (n = 41); (3) the image quality was poor (*n* =16); (4) patients with previous antitumor treatment, including neoadjuvant therapy, conversion therapy, or palliative therapy (*n* = 5). Finally, 292 patients were included in this study.

**Figure 1:**
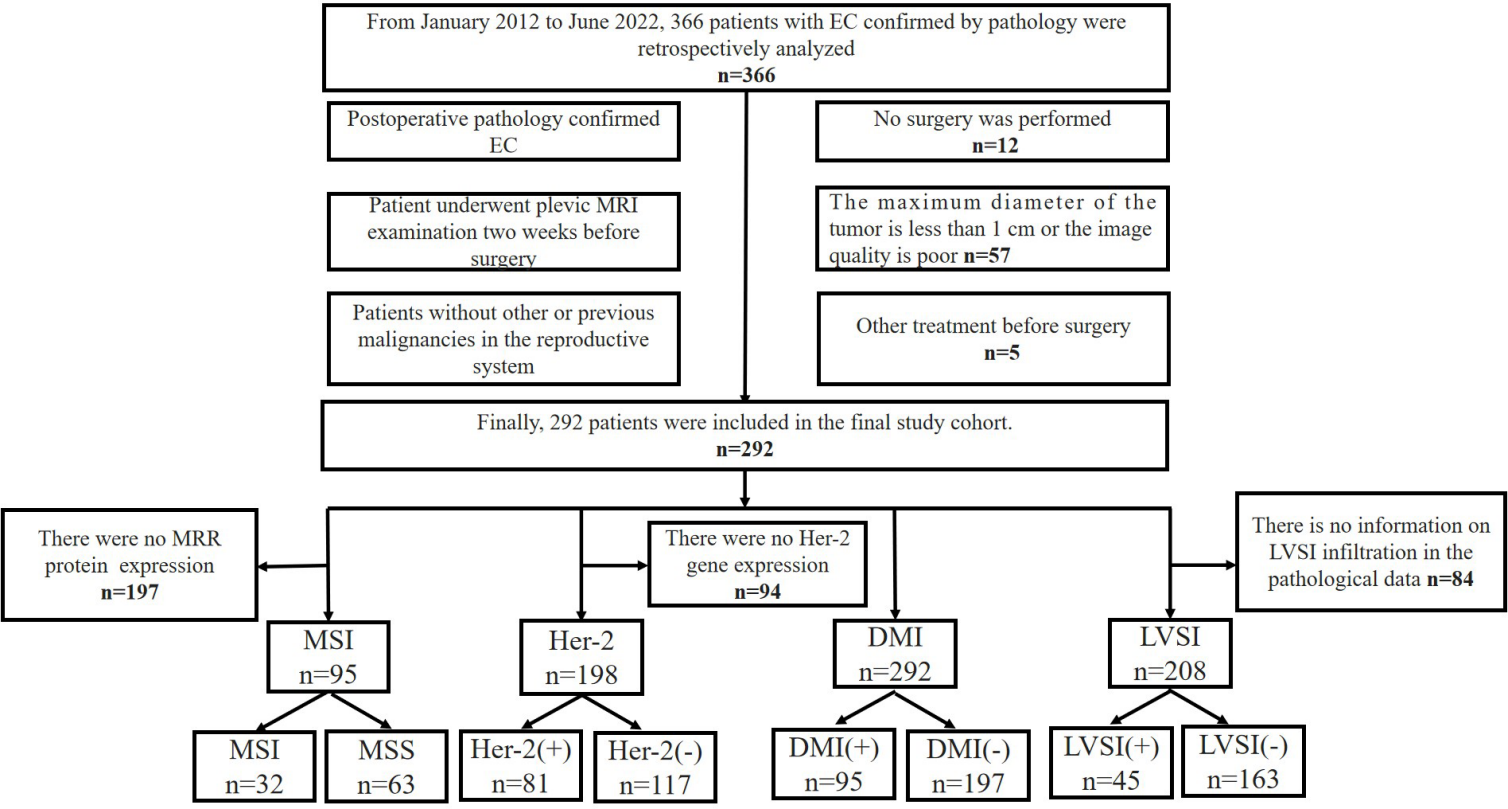
Flowchart of the recruitment pathway for patients. EC, endometrial cancer; MSI, microsatellite instability; Her-2, human epidermal growth factor receptor-2; DMI, deep myometrium invasion; LVSI, lympho-vascular space invasion.

Patients were classified into 4 analysis subsets, including the followings: (1) the MSI subset, from which 197 cases were excluded because of the absence of MRR protein expression in the immunohistochemical indexes; (2) the Her-2 subset excluded 94 cases due to the absence of data about Her-2 gene expressions in the immunohistochemical indices; (3) the LVSI subset, from which 84 cases were excluded due to the absence of LVSI in the pathological information; and (4) the DMI subset, where all enrolled patients were included. In each subset, the patients were divided into two layers according to their positive or negative indicators, after which random sampling was conducted from each layer according to the ratio of 8:2 (training cohort: validation cohort) in MSI and Her-2 subsets or the ratio of 7:3 (training cohort: validation cohort) in DMI and LVSI subsets. **Figure 1** illustrates the recruitment pathways for patients.

General clinical information, including age, irregular vaginal bleeding (IVB), pathological type, Federation International of Gynecology and Obstetr (FIGO) stage, and menopausal state, were collected within one week before surgery.

### MR data acquisition

MRI examination was performed using a 1.5T MR system (Signa, HDXT, GE Healthcare) with an 8-channel phased array body coil and a 3.0T MR system (Ingenia CX, Philips Healthcare, Best, the Netherlands) with a 32-channel abdominal coil (GE 1.5T MR system: n= 236; Philips 3.0T MR system: n=56). Before the examination, the patients were instructed to empty the bladder, and their intrauterine device (IUD) were taken out one day before the examination. The patients were scanned in the supine position, with legs and knees relaxed and not overstretched. MRI sequences included T_2_WI and DWI. The original DWI images were transmitted to the ADW 4.6 Workstation, and the Functool function was applied to generate apparent diffusion coefficient (ADC) images. The detailed scanning parameters are shown in **Supplementary Data S1**.

### Biological characteristics assessment

The diagnosis of DMI and LVSI was determined by hematoxylins and eosin staining. Histopathology was performed to determine the status of immunohistochemical characteristics, including MSI and Her-2. Two pathologists were blinded to the clinical and imaging data, evaluated the biological characteristics. All inconsistencies were resolved by discussion; in addition, a third pathologist (senior pathologist with 10 years of pathology experience) was invited to confirm the data. The criteria for each biological characteristic were as follows: (1) MSI: the expression of mismatch repair (MMR) protein (MLH-1, MSH-2, MSH-6, and PMS-2) was defined as MSS when all four MMR proteins were expressed, and MSI with at least one MMR protein was not expressed (22); (2) Her-2: immune-histochemical images were taken under a high-magnification microscope. The three fields of view were randomly selected for each tissue section, and the percentage of positive cells was divided into the four following grades: 0 points for < 10%, 1 point for 10%-25%, 2 points for 25%-50%, 3 points for 50%-75%, and 4 points for >75%. Scoring was performed according to the degree of coloration, where 0 was used if there was no color, 1 point for light yellow, 2 points for brownish yellow, and 3 points for tan. For comprehensive judgment, the percentage of positive cells and the degree of staining was calculated: 0, 1+, 2+, 3+, 4+ points (0-1+ was considered negative, ≥2+ was considered positive) (23); (3) LVSI: LVSI was defined in accordance with the three-grade system as follows: none (no LVSI), focal (a single focus of LVSI was recognized around a tumor), and substantial (diffuse or multifocal LVSI was recognized around the tumor, or massive LVSI was recognized in the myometrium with a spray-like growth, regardless of the degree of myometrium invasion) (24); (4) DMI: DMI was defined as an infiltration 50% of myometrium wall thickness, which was considered as the most important single morphological prognostic factor.

### Tumor segmentation and radiomics feature extraction

The T_2_WI, DWI, and ADC images stored in digital imaging and communications in medicine (DICOM) format were exported from the picture archiving and communication system (PACS) and used for image preprocessing and tumor segmentation. To avoid data heterogeneity bias, all MRI data were performed for image normalization (the intensity of the image was scaled to 0-100) and resampled to the same resolution (1 × 1 × 1 mm^3^) before tumor segmentation using A.K. software (Artificial Intelligence Kit, Version 3.2.5, GE Healthcare). The ITK-SNAP software (Version3.6, open-source software, http://www.itksnap.org) was used to delineate the region of interest (ROI) around the tumor margin on each slice of T_2_WI, DWI, and ADC images by two experienced radiologists (Tian SF and Ma CJ, with 8 years and 3 years of experience in uterine MRI, respectively) who were blinded to the clinical and pathological information of the patients. The ROIs were placed to avoid including nearby normal myometrium or endometrium. To assess the intra-observer and inter-observer reproducibility, reader 1 performed the segmentation of 30 randomly selected patients twice at the one-month interval, and reader 2 independently performed the segmentation of 30 patients following the same procedure. The segmentation of the remained patients was performed by reader 1. Finally, 772 radiomics features of each sequence, including 14 shape features, 66 first-order features, 306 texture features, and 386 Gaussian transform features, were extracted from the VOIs by using A. K. software. Details of radiomics features are listed in **Supplementary Data S2**.

The Synthetic Minority Oversampling Technique (SMOTE) method was used because of unbalance of positive/negative LVSI samples in the training and validation cohort. Positive LVSI (minority class) was oversampled and negative LVSI (majority class) was under sampled to balance the training cohort to improve the classification performance.

### Feature selection, model construction and model evaluation

**Figure 2:**
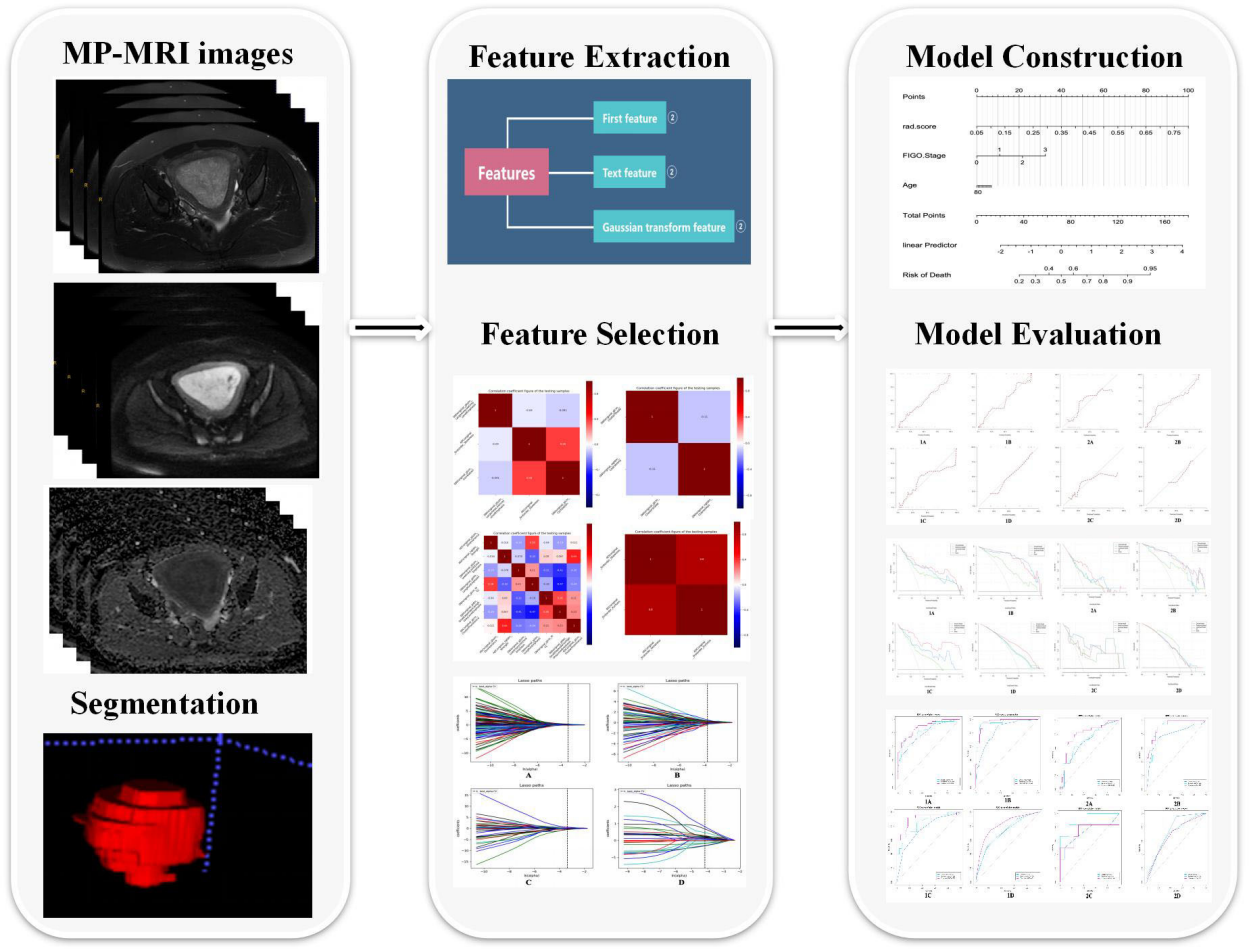
The workflow of radiomics analysis in our study.

A four-step procedure was devised for dimensionality reduction (**Figure 2**). Firstly, in order to ensure the robustness and reproducibility of the model, the radiomics features with high stability in both intra-observer and inter-observer stability (ICC≥0.9) were selected for subsequent analysis. Secondly, Spearman’s rank correlation test was applied to exclude the redundant features (correlation coefficient values≥0.9), after which the features with significant differences between the two groups were selected using univariate logistic regression. Finally, the least absolute shrinkage and selection operator (LASSO) was used to select non-zero coefficient features associated with DMI, LVSI, MSI, and Her-2 in EC patients with 5-fold cross-validation by the penalty parameter to avoid overfitting. Five kinds of classifiers, including logistic regression, random forest (RF), decision tree, K-nearest neighbor (KNN), and Bayes, were used to construct MP-MRI radiomics models for predicting biological characteristics in EC. The best *k* value (number of neighbors) for KNN was found by training in the range of 3–10. For random forest, decision tree, and Bayes, the maximum tree depth was constrained to avoid overfitting (25). The parameters used in the construction of MP-MRI models were listed in **Supplementary Data S3**. The univariate and multivariate analyses were used to assess the association between the clinical characteristics and DMI, LVSI, MSI, or Her-2. The significant clinical risk factors were used to develop and validate clinical models for DMI, LVSI, MSI, and Her-2. Meanwhile, clinical independent risk factors were integrated into the corresponding MP-MRI radiomics models to construct the combined models. Finally, we built a radiomics nomogram with both the radscore and clinical independent risk factors. Calibration curves (Hosmer-Lemeshow H test) were used to evaluate the calibration of the model, and receiver operating characteristic (ROC) curves were used to assess the diagnostic efficiency. The clinical useful of the combined nomograms were evaluated with decision curve analysis (DCA).

### Statistical analysis

All statistical analyses were conducted with R software (Version 4.0.2; http://www.r-project.org). A two-sided *P* value < 0.05 was considered statistically significant. Student’s *t-*test or Mann-Whitney *U* test was used to compare quantitative variables, and the Chi-squared test or Fisher’s exact test was used to compare qualitative variables. The discrimination performances of radiomics models, clinical models, and combined models for predicting biological characteristics in EC were evaluated according to the area under the receiver operator characteristic (ROC) curve (AUC) in both training and validation cohorts. Delong’s test was used to assess the difference between the AUC values of the model. Calibration curve was used to assess the goodness of fit of the radiomics nomogram in the training and validation cohorts. Decision curve analysis (DCA) was performed to determine the clinical usefulness of the prediction models by quantifying the net benefits at different threshold probabilities.

## Results

### Patient profiles

A total of 292 EC patients were included in the study. According to their pathological results, they were divided into four subsets: DMI (n = 292), LVSI (n = 208), MSI (n = 95), and Her-2 (n = 198). Clinical and histopathological characteristics of patients with EC in the training and validation cohorts are summarized in **Table 1**. There were 32 (33.68%) patients in the MSI group and 63 (66.32%) in the microsatellite stabilization (MSS) group. In addition, 198 patients were assigned to the Her-2 subset, including 81 in the Her-2 positive group and 117 in the Her-2 negative group. For the DMI subset, 95 (32.53%) were in the DMI positive group and 197 (67.47%) were in the DMI negative group. For the LVSI subset, 208 patients were assigned to the LVSI subgroup, including 45(21.63%) in the LVSI positive group and 163(78.37%) in the LVSI negative group. No significant differences were observed in the clinical and histopathological characteristics between the training and validation cohort (P>0.05), except for menopausal state in Her-2 subset, differentiation degree in DMI subset, differentiation degree and FIGO stage in LVSI subset between training and validation cohorts.

**Table 1:** Patient profiles of each subset.

Parameters	*Training cohort*	*Validation cohort*	*P*
MSI subset	n = 76	n = 19	
Age	58.33 ± 10.37	60.53 ± 12.16	0.427
Differentiation degree n/%			
High	20/26.32%	4/21.05%	
Median	32/42.11%	12/63.16%	0.244
Low	24/31.57%	3/15.79%	
Pathological type n/%			
Type I	62/81.58%	16/84.21%	1.000
Type II	14/18.42%	3/15.79%	
FIGO Stage n/%			
I	46/60.53%	13/17.11%	
II	17/19.77%	2/10.53%	0.598
III	11/14.47%	3/15.79%	
IV	2/2.63%	1/5.26%	
Menopausal state n/%			
Before	24/31.58%	3/15.79%	0.256
After	52/68.42%	16/84.21%	
IVB n/%			
Yes	47/61.84%	11/57.89%	0.796
No	29/38.16%	8/42.11%	
**Her-2 subset**	**n = 158**	**n = 40**	
Age	57.63 ± 10.63	61.10 ± 9.94	0.064
Differentiation degree n/%			
High	41/25.95%	12/30.00%	
Median	78/49.37%	17/42.50%	0.741
Low	39/24.68%	11/27.50%	
Pathological type n/%			
Type I	138/87.34%	34/85.00%	0.793
Type II	20/12.66%	6/15.00%	
FIGO Stage n/%			
I	110/69.62%	30/75.00%	
II	27/17.09%	5/12.50%	0.871
III	18/11.39%	4/10.00%	
IV	3/1.90%	1/2.50%	
Menopausal state n/%			
Before	57/36.08%	6/15.00%	***0.013***
After	101/63.92%	34/85.00%	
IVB n/%			
Yes	101/63.92%	27/67.50%	
No	57/36.07%	13/32.50%	
**DMI subset**	**n = 204**	**n = 88**	
Age	58.73 ± 10.42	56.81 ± 10.90	0,156
Differentiation degree n/%			
High	70/34.31%	16/18.18%	
Median	85/41.67%	40/45.45%	***0.010***
Low	49/24.02%	32/36.37%	
Pathological type n/%			
Type I	167/81.86%	77/87.50%	0.302
Type II	37/18.14%	11/12.50%	
FIGO Stage n/%			
I	155/75.98%	57/64.77%	
II	22/10.78%	16/18.18%	0.057
III	25/12.25%	11/12.50%	
IV	2/0.99%	4/4.55%	
Menopausal state n/%			
Before	62/30.39%	34/38.64%	0.177
After	142/69.61%	54/61.36%	
IVB n/%			
Yes	138/67.65%	62/70.45%	0.682
No	66/32.35%	26/29.55%	
**LVSI subset**	**n = 146**	**n = 62**	
Age			
Differentiation degree n/%			
High	48/32.88%	9/14.52%	
Median	57/39.04%	33/53.23%	***0.018***
Low	41/28.08%	20/32.26%	
Pathological type n/%			
Type I	118/80.82%	50/80.65%	1.000
Type II	28/19.18%	12/19.35%	
FIGO Stage n/%			
I	114/70.08%	32/51.61%	
II	12/8.22%	14/22.58%	***0.001***
III	19/13.01%	12/19.35%	
IV	1/0.69%	3/4.84%	
Menopausal state n/%			
Before	46/31.51%	18/29.03%	0.406
After	100/68.49%	44/70.97%	
IVB n/%			
Yes	91/62.33%	47/75.81%	0.077
No	55/37.67%	15/24.19	

MSI, microsatellite instability; Her-2, human epidermal growth factor receptor-2; DMI, deep myometrium invasion; LVSI, lympho-vascular space invasion; FIGO, Federation International of Gynecology and Obstetrics; IVB, Irregular vaginal bleeding.The meaning of bold and italic values are the number of patients in each subgroup and statistically significant data.

### Feature selection and radiomics model building

**Figure 3:**
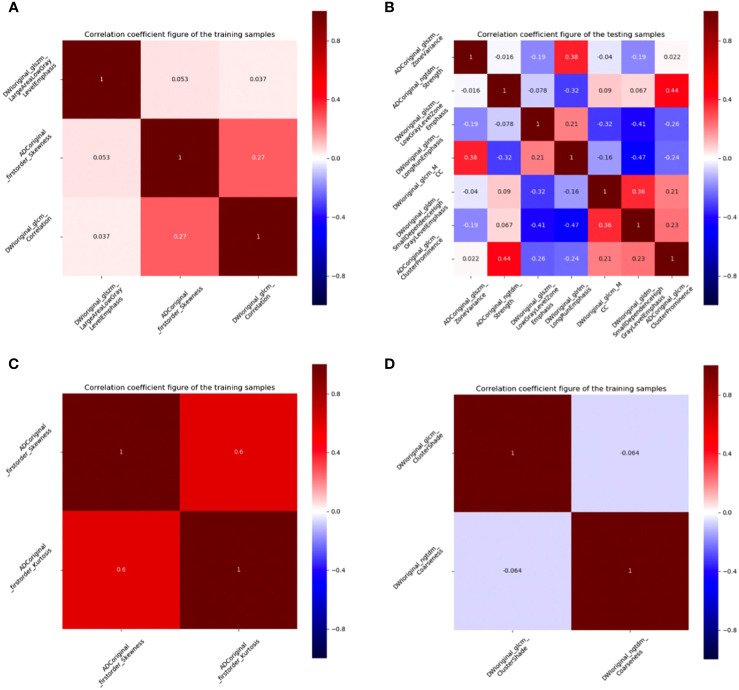
Correlation coefficient figures of the remaining features. **(A)** DMI; **(B)** LVSI; **(C)** MSI; **(D)** Her-2. EC, endometrial cancer; MSI, microsatellite instability; Her-2, human epidermal growth factor receptor-2; DMI, deep myometrium invasion; LVSI, lympho-vascular space invasion.

**Figure 4:**
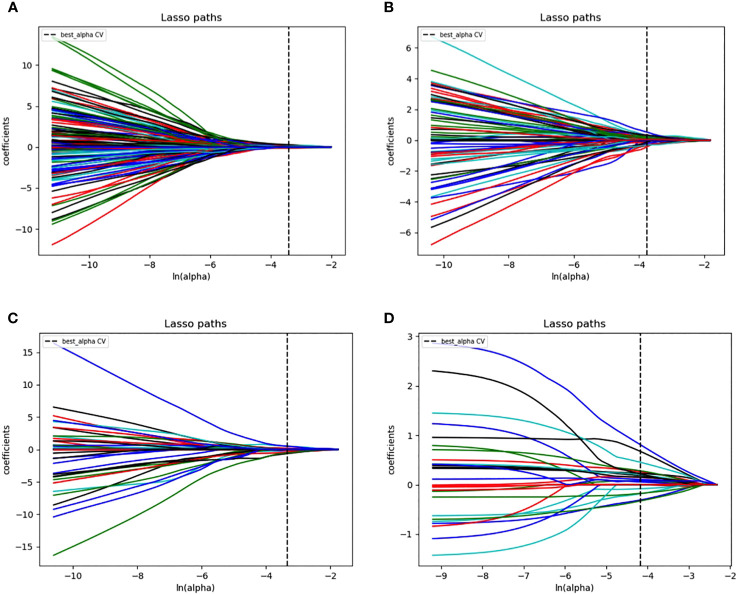
LASSO regression figures. Radiomics features were selected by LASSO regression. **(A)** DMI; **(B)** LVSI; **(C)** MSI; **(D)** Her-2. EC, endometrial cancer; MSI, microsatellite instability; Her-2, human epidermal growth factor receptor-2; DMI, deep myometrium invasion; LVSI, lympho-vascular space invasion.

First, features with ICC values < 0.9 were excluded, and the radiomics features of DMI, LVSI, MSI, and Her-2 subsets were reduced from 2316 to 1199. Details of ICC values were listed in **Supplementary Data S4**. Among the remaining features, 409, 366, 390, and 409 features of DMI, LVSI, MSI, and Her-2 subsets were retained with correlation coefficients > 0.9 by Spearman’s correlation test. Next, 174, 122, 61, and 23 features of DMI, LVSI, MSI, and Her-2 subsets were retained using univariate analysis. Finally, 3, 7, 2, and 2 features were selected *via* LASSO regression (**Figure 3** and **Figure 4**). The intra-observer and inter-observer reproducibility was high.

### Performance of radiomics models on different classifiers

The predictive performance of five classifiers (logistic regression, RF, decision tree, KNN, and Bayes) is listed in **Table 2**. In general, the performance of the RF was the best according to AUC values, so the RF was chosen as the best prediction model for the three subsets (MSI: AUC_training_ = 0.844, AUC_validation_= 0.897; LVSI: AUC_training_ = 0.952, AUC_validation_= 0.908; DMI: AUC_training_ = 0.840, AUC_validation_= 0.739). As for the Her-2 subset, the performance of the decision tree was relatively higher (AUC_training_ = 0.714, AUC_validation_ = 0.708), so a decision tree with a polynomial kernel function was selected as the optimal classifier for the Her-2 subset.

**Table 2:** Discriminative performance of different MP-MRI radiomics classifiers for predicting the four biological characteristics.

Different models	Training cohort				Validation cohort			
	AUC	SEN	SPE	ACC	AUC	SEN	SPE	ACC
MSI								
Logistic regression	0.747	0.640	0.725	0.697	0.782	0.500	0.923	0.789
Random forest	**0.844**	**0.560**	**0.902**	**0.789**	**0.897**	**0.667**	**1.000**	**0.895**
Decision tree	0.825	0.480	1.000	0.829	0.776	0.500	0.846	0.737
KNN	0.753	0.440	0.843	0.711	0.705	0.500	0.769	0.684
Bayes	0.784	0.520	0.843	0.737	0.769	0.500	0.846	0.737
Her-2								
Logistic regression	0.636	0.602	0.646	0.620	0.669	0.708	0.562	0.650
Random forest	0.747	0.742	0.554	0.665	0.690	0.708	0.500	0.625
Decision tree	**0.714**	**0.634**	**0.692**	**0.658**	**0.704**	**0.708**	**0.625**	**0.675**
KNN	0.704	0.645	0.631	0.639	0.651	0.833	0.500	0.700
Bayes	0.626	0.570	0.508	0.544	0.641	0.625	0.562	0.600
DMI								
Logistic regression	0.726	0.576	0.819	0.740	0.724	0.517	0.864	0.750
Random forest	**0.840**	**0.561**	**0.899**	**0.789**	**0.739**	**0.448**	**0.932**	**0.773**
Decision tree	0.773	0.636	0.848	0.779	0.726	0.517	0.864	0.750
KNN	0.800	0.409	0.928	0.760	0.713	0.414	0.898	0.739
Bayes	0.702	0.485	0.826	0.716	0.743	0.552	0.780	0.705
LVSI								
Logistic regression	0.831	0.800	0.823	0.811	0.831	0.735	0.914	0.826
Random forest	**0.952**	**0.937**	**0.888**	**0.912**	**0.908**	**0.943**	**0.765**	**0.855**
Decision tree	0.858	0.862	0.747	0.805	0.823	0.882	0.657	0.768
KNN	0.868	0.772	0.812	0.792	0.856	0.771	0.882	0.826
Bayes	0.801	0.612	0.861	0.736	0.792	0.647	0.771	0.710

MSI, microsatellite instability; Her-2, human epidermal growth factor receptor-2; DMI, deep myometrium invasion; LVSI, lympho-vascular space invasion; AUC, area under the curve; SEN, sensitivity; SPE, specificity; ACC, accuracy; KNN, K-nearest neighbor; RF, Random Forest.The meaning of bold values are the best performance classifiers.

### Performance of clinical and combined models

After the univariate and multivariate logistic analysis, two characteristics were included for MSI (differentiation degree and IVB), two for Her-2 (IVB and FIGO stage), two for DMI (age and FIGO stage), and three for LVSI (differentiation degree, IVB, and FIGO stage) (**Table 3**). Clinical characteristics were then added to the optimal MP-MRI radiomics model to construct the combined models.

**Table 3:** Statistical analysis of univariate and multivariate logistic analyses.

Subgroups	Parameters	Univariate Analysis		Multivariate Analysis	
		OR (95%CI)	*P*	OR (95%CI)	P
**MSI**	Age	1.053 (1.001 - 1.107)	***0.044***	/	/
	Differentiation degree	2.345 (1.168 - 4.706)	***0.017***	2.325 (1.130 - 4.784)	***0.022***
	Menopausal state	3.387 (1.012 - 11.336)	***0.478***	/	/
	IVB	3.555 (1.156 - 10.936)	***0.027***	3.492 (1.092 - 11.170)	***0.035***
	Pathologic type	3.530 (1.067 - 11.678)	***0.039***	/	/
**Her-2**	FIGO stage	0.576 (0.376 - 0.883)	***0.011***	0.537 (0.340 - 0.850)	***0.008***
	IVB	0.242 (0.114 - 0.510)	***0.000***	0.223 (0.103 - 0.484)	***0.000***
	Differentiation degree	0.664 (0.422 - 1.046)	***0.078***	/	/
**DMI**	Age	1.062 (1.028 - 1.096)	***0.000***	1.065 (1.030 - 1.102)	***0.000***
	Differentiation degree	1.658 (1.116 - 2.465)	***0.012***	/	/
	Menopausal state	2.557 (1.250 - 5.229)	***0.010***	/	/
	FIGO stage	2.351 (1.565 - 3.529)	***0.000***	2.469 (1.596 - 3.821)	***0.000***
**LVSI**	FIGO stage	2.208 (1.539 - 3.169)	***0.000***	2.278 (1.532 - 3.386)	***0.000***
	IVB	3.491 (1.709 - 7.130)	***0.001***	3.466 (1.576 - 7.623)	***0.002***
	Differentiation degree	1.952 (1.280 - 2.976)	***0.002***	1.759 (1.108 - 2.791)	***0.017***
	Pathologic type	3.652 (1.577 - 8.461)	***0.003***	/	/

MSI, microsatellite instability; Her-2, human epidermal growth factor receptor-2; DMI, deep myometrium invasion; LVSI, lympho-vascular space invasion; FIGO, Federation International of Gynecology and Obstetr; OR, odds ratio; CI, confidence interval.The meaning of bold and italic values are statistically significant data.

**Figure 5:**
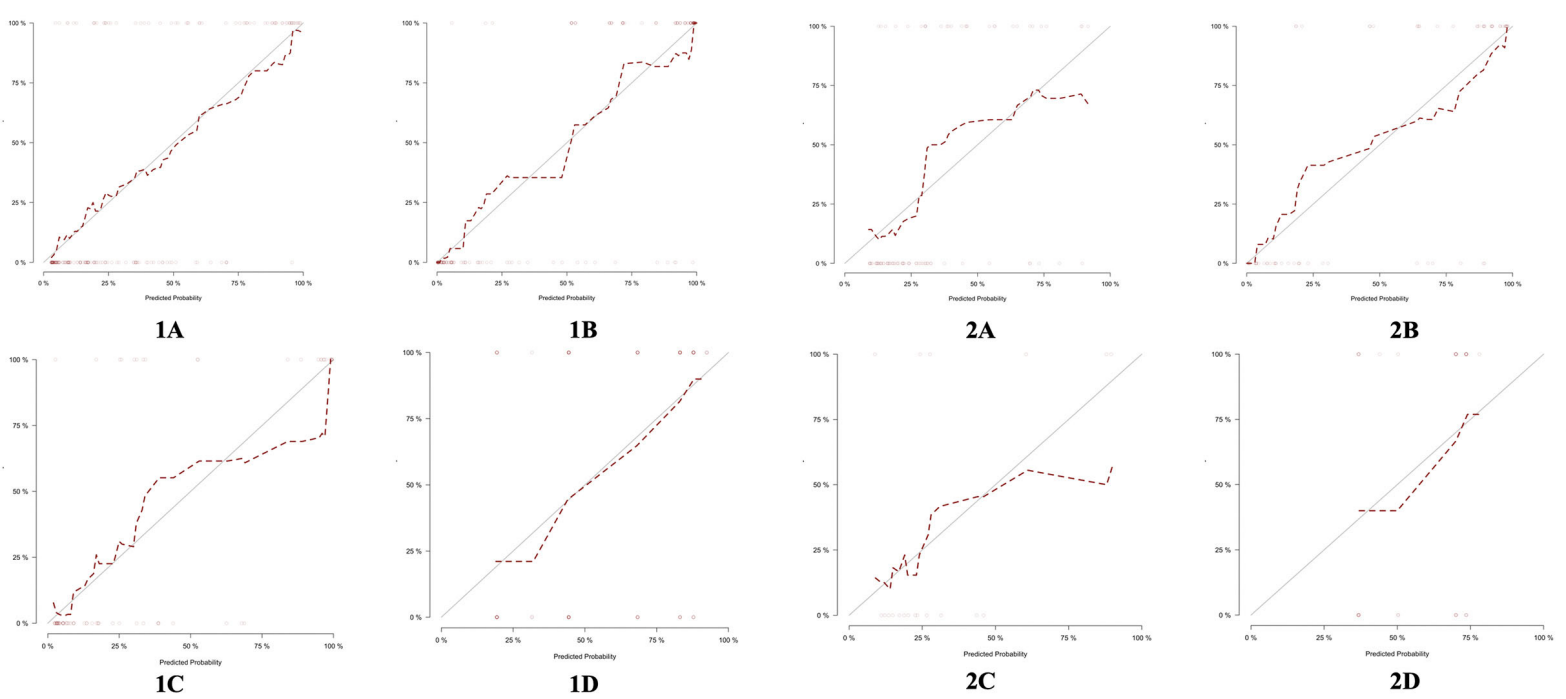
Calibration curves of combined models in the training cohort (1) and the validation cohort (2). Calibration curves showed the calibration of the predictive model for the risk of biological characteristics in EC: **(A)** DMI; **(B)** LVSI; **(C)** MSI; **(D)** Her-2. EC, endometrial cancer; MSI, microsatellite instability; Her-2, human epidermal growth factor receptor-2; DMI, deep myometrium invasion; LVSI, lympho-vascular space invasion.

**Figure 6:**
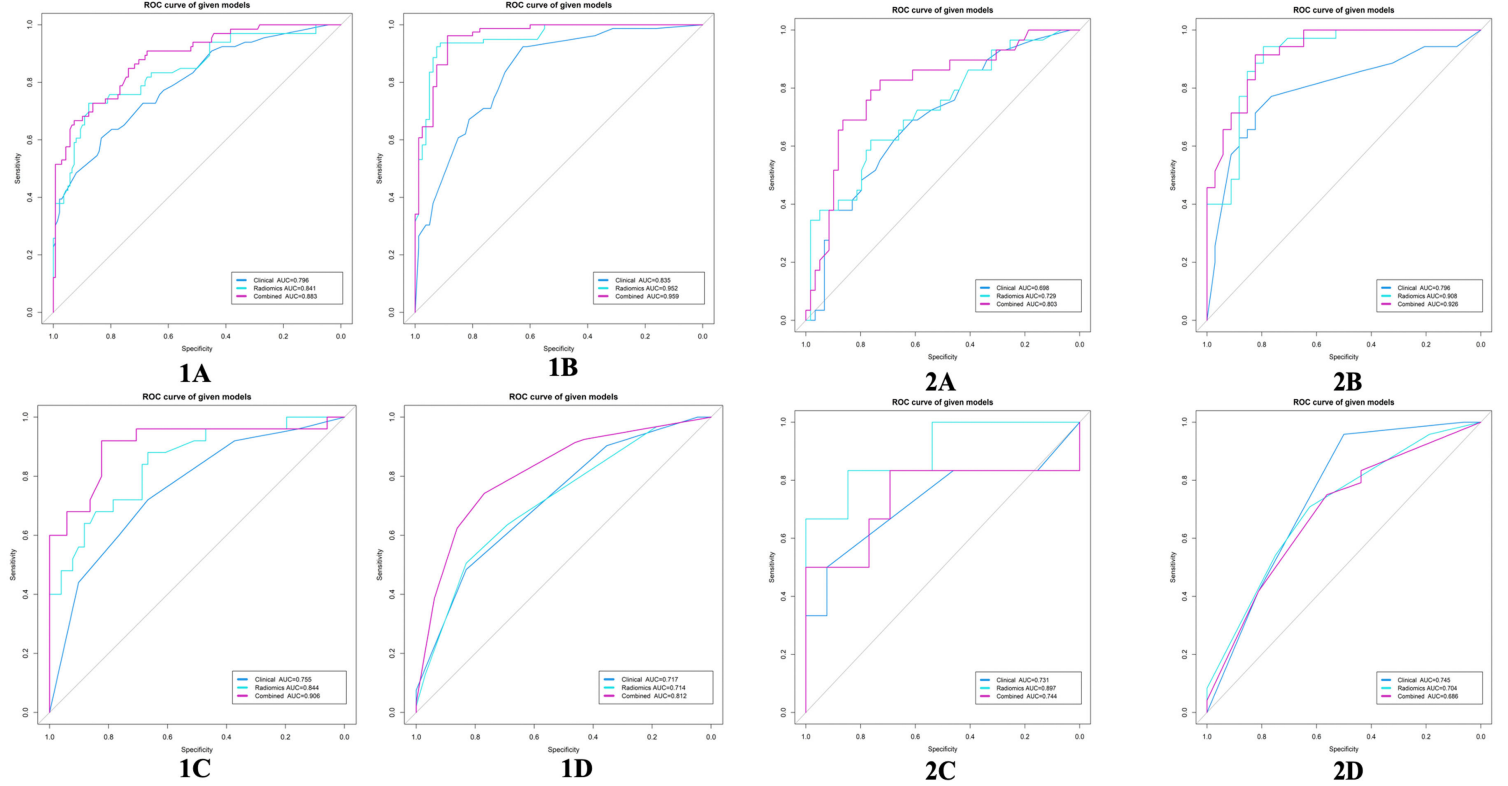
ROC curves for the clinical model, radiomics model, and combined model in the training cohort (1) and validation cohort (2). **(A)** DMI; **(B)** LVSI; **(C)** MSI; **(D)** Her-2. EC, endometrial cancer; MSI, microsatellite instability; Her-2, human epidermal growth factor receptor-2; DMI, deep myometrium invasion; LVSI, lympho-vascular space invasion; ROC, Receiver operating characteristic.

**Figure 7:**
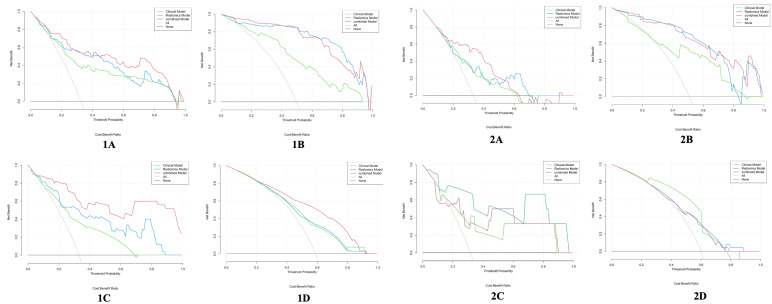
Decision curve analysis for the clinical model, radiomics model, and combined model in the training cohort (1) and the validation cohort (2). **(A)** DMI; **(B)** LVSI; **(C)** MSI; **(D)** Her-2. The y-axis represents the net benefits, and the x-axis represents the threshold probability. EC, endometrial cancer; MSI, microsatellite instability; Her-2, human epidermal growth factor receptor-2; DMI, deep myometrium invasion; LVSI, lympho-vascular space invasion.

**Figure 8:**
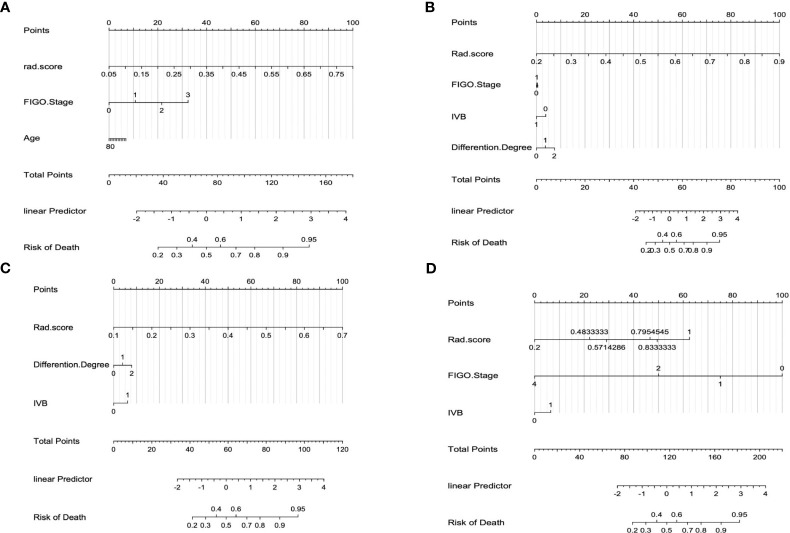
The interpretation of the radiomics nomogram. **(A)** Combined nomogram of DMI subset; **(B)** Combined nomogram of LVSI subset; **(C)** Combined nomogram of Her-2 subset; **(D)** Combined nomogram of MSI subset.

The calibration curve of the combined model of the four subsets demonstrated that the predicted values were in good agreements with the observed values (**Figure 5**). The Hosmer-Lemeshow H test showed that the statistical results in the training cohort (MSI: *P*=0.619, Her-2: *P*=1.000, DMI: *P*=0.549, LVSI: *P*=0.250) and validation cohort (MSI: *P*=0.125, Her-2: *P*=0.925, DMI: *P*=0.209, LVSI: *P*=0.102) were not significant. In the training cohort, the AUCs of the combined model were significantly higher than the clinical model for predicting MSI, Her-2, DMI, and LVSI (MSI, *AUC_combined_ = 0.907 vs. AUC_clinical_ = 0.755, P* = 0.002; Her-2, *AUC_combined_ = 0.812 vs. AUC_clinical_ = 0.717*, *P = 0.011*; DMI, *AUC_combined_ = 0.883 vs. AUC_clinical_ = 0.796, P = 0.004*; LVSI, *AUC_combined_ = 0.959 vs. AUC_clinical_ = 0.835, P<0.05*). In the validation cohort, there were significant differences in the AUCs of the two models only for the prediction of DMI and LVSI (DMI, *AUC_combined_ =0.803 vs. AUC_clinical_ =0.698, P = 0.033*; LVSI, *AUC_combined_ = 0.926 vs. AUC_clinical_ = 0.796, P = 0.002*) (**Table 4**, **Table 5**, and **Figure 6**). The DCA for the combined models is presented in **Figure 7**. The DCA indicated that the combined models have more benefits than the “treat-all strategy” and “treat-none strategy” when the range of threshold probability was >21%. Nomogram visualizes the combined model to show the likelihood of the occurrence of various biological characteristics of individual EC (**Figure 8**).

**Table 4:** Discriminative performance of optimal models for each biological characteristic.

Different models	Training cohort				Validation cohort			
	AUC (95%CI)	SEN	SPE	ACC	AUC (95%CI)	SEN	SPE	ACC
MSI								
Radiomics model	0.844 (0.756 – 0.918)	0.560	0.902	0.789	0.897 (0.731 – 1.000)	0.667	1.000	0.895
Clinical model	0.755 (0.654 – 0.848)	0.440	0.902	0.750	0.731 (0.469 – 0.949)	0.433	1.000	0.789
Combined model	0.907 (0.829 – 0.969)	0.600	1.000	0.868	0.744 (0.455 – 0.974)	0.500	1.000	0.842
Her-2								
Radiomics model	0.714 (0.650 – 0.777)	0.634	0.692	0.658	0.704 (0.567 – 0.834)	0.708	0.625	0.675
Clinical model	0.717 (0.655 – 0.777)	0.903	0.354	0.677	0.745 (0.611 – 0.871)	0.958	0.500	0.775
Combined model	0.812 (0.752 – 0.866)	0.742	0.769	0.753	0.686 (0.549 – 0.820)	0.750	0.562	0.675
DMI								
Radiomics model	0.840 (0.787– 0.890)	0.561	0.899	0.789	0.739 (0.629 – 0.823)	0.448	0.932	0.773
Clinical model	0.796 (0.737 – 0.848)	0.303	0.993	0.770	0.698 (0.595 – 0.796)	0.310	0.915	0.716
Combined model	0.883 (0.839 – 0.920)	0.530	0.957	0.819	0.803 (0.717 – 0.886)	0.414	0.898	0.739
LVSI								
Radiomics model	0.952 (0.924 – 0.977)	0.937	0.888	0.912	0.908 (0.844 – 0.963)	0.943	0.765	0.855
Clinical model	0.835 (0.782 – 0.885)	0.772	0.712	0.742	0.796 (0.702 – 0.884)	0.714	0.824	0.768
Combined model	0.959 (0.935 – 0.981)	0.962	0.862	0.912	0.926 (0.871 – 0.968)	0.914	0.824	0.870

MSI, microsatellite instability; Her-2, human epidermal growth factor receptor-2; DMI, deep myometrium invasion; LVSI, lympho-vascular space invasion; AUC, area under the curve; SEN, sensitivity; SPE, specificity; ACC, accuracy.The meaning of bold and italic values are different subsets.

**Table 5:** Comparison of different models for evaluating the biological characteristics of EC.

	Training cohort	Validation cohort
MSI		
Radiomics model VS Clinical model	0.202	0.346
Combined model VS Clinical model	***0.002***	0.843
Combined model VS Radiomics model	0.153	0.403
Her-2		
Radiomics model VS Clinical model	0.952	0.742
Combined model VS Clinical model	***0.011***	0.374
Combined model VS Radiomics model	***0.015***	0.851
DMI		
Radiomics model VS Clinical model	0.319	0.706
Combined model VS Clinical model	***0.004***	***0.033***
Combined model VS Radiomics model	0.104	0.148
LVSI		
Radiomics model VS Clinical model	***0.000***	0.086
Combined model VS Clinical model	***0.000***	***0.002***
Combined model VS Radiomics model	0.634	0.632

The data in the table represents the p-value; EC, endometrial cancer; MSI, microsatellite instability; Her-2, human epidermal growth factor receptor-2; DMI, deep myometrium invasion; LVSI, lympho-vascular space invasion.

## Discussion

In the present study, we constructed radiomics models based on MP-MRI (T_2_WI, DWI, and ADC) using five classifiers to predict four prognosis-related biological characteristics (DMI, LVSI, MSI, and Her-2) in EC patients. Our study showed that the optimal radiomics models obtained moderate to positive performances in the training cohort (AUC:0.714 - 0.952) and in the validation cohort (AUC:0.704 - 0.908). Furthermore, we added several clinical characteristics to the optimal radiomics models for the combined models building, and our combined models showed a satisfactory performance. To the best of our knowledge, among all the reported radiomics studies for predicting risk factors of EC, our study is the first study that assess the expression of Her-2 gene in EC based on MP-MRI radiomics approach.

The challenge in preoperative staging and surgical planning of EC is the assessment of biological risk factors such as DMI, LVSI, etc. Radiomics approaches can provide a comprehensive, non-invasive, and reproducible assessment of tumor biology. Recently, the research on the evaluation of multiple tumor biological characteristics based on MP-MRI radiomics had been carried out in rectal cancer (12) and prostate cancer (25). However, to the best of our knowledge, only a few studies reported on the comprehensive prediction of various biological characteristics of EC. Lefebvre et al. (26) recently evaluated the performance of MP-MRI (T_2_WI, DWI, and CE-T_1_WI sequences) radiomics models for differentiating low- from high-risk histopathologic markers-DMI, LVSI, and high-grade status and advanced-stage EC. Moreover, Otani et al. (27) evaluated radiomics classifiers based on MP-MRI (T_2_WI, DWI, and CE-T_1_WI sequences) in the pretreatment assessment of risk factors (DMI, LNM, LVSI, and histological grade) of EC patients. Yet, these studies did not comprehensively evaluate the aggressiveness of EC. In this study, we further assessed various immunohistochemical markers associated with tumor aggressiveness of EC. Therefore, our comprehensive radiomics models made it possible to predict more critical biological characteristics of EC and improve the prediction accuracy of some biological characteristics compared with previous radiomics models in published studies.

In this study, the selected radiomics features after the four-step dimensionality reduction method are dominated by first-order features, and the radiomics features of ADC images accounted for a large part, which could be due to the following reasons: (1) since the ADC map is derived from the DWI sequence, it mainly reflects the fluidity of water molecules around the lesion, which is affected by various factors, such as cell density, membrane integrity, and microstructural heterogeneity, and is more accurate in reflecting aggressive of tumor lesions; (2) the radiomics features of T_2_WI and DWI are extracted based on image signal intensity, and the advantage of image signal intensity is usually the ability to distinguish tumors from surrounding normal tissues, rather than describing the internal characteristics of tumors. Therefore, the ADC value is an objective quantitative indicator often used to reflect the internal characteristics of tumors, especially tumor aggressiveness (28). In additional, among the five classifiers (KNN, RF, decision tree, logistics regression, and Bayes) included in this study, the classification performance of RF was generally the best, this may be because that RF classifiers can reduce the model’s dependence on specific features and samples by combining multiple decision trees for prediction, and the problem of overfitting of a single decision tree can be reduced, at the same time, RF classifiers have strong robustness to deal with noisy data (29, 30).

The use of radiomics methods to evaluate Her-2 gene expression has been widely explored in breast cancer (31), gastric cancer (32), and other diseases (33). Our study explored the role of radiomics in predicting Her-2 status in EC patients and constructed a clinical model based on clinical-pathological information. Our study further integrated independent risk factors and radscore to establish a combined model with an AUC of 0.812 in the training cohort, which was higher than clinical model and the radiomics model. In our study, two clinical-pathological features, including IVB and differentiation degree, as independent risk factors for predicting Her-2 gene expression in EC, were highly correlated with Her-2 gene status, which was consistent with the finding of Morrison et al. (5). However, in the validation cohort, the clinical model obtained the highest AUC among the three models, which was owing to the small sample size of validation cohort and heterogeneity of tumors. In actual fact, the expression of Her-2 gene was mainly related to clinicopathological characteristics, such as LVSI, LNM, and differentiation degree. Furthermore, the differentiation degree of pathological indexes was included in the construction of clinical model in our study, which was attributed to their response to tumor heterogeneity, thus increased the evaluation effectiveness of clinical model to a certain extent.

Detection of MSI status in EC patients can help screen the Lynch syndrome and evaluate disease progress, thus provide personalized and precise treatments (8). For example, PD-1/PD-L1 are highly expressed in EC patients with MSI, both of which can inhibit the proliferation and differentiation of T cells, owing to inactivate the T cell function and diminish the inhibitory effect on tumor cells (34). PD-1/PD-L1 inhibitors can restore the inhibitory effect of T cells on tumor cells, and can be targeted to EC patients with MSI (35). At the same time, MSI as one of the molecular types of endometrial cancer (36), recent evidence suggests that the evaluation of molecular and genomic profiling provides an accurate method to assess the prognosis of endometrial cancer patients (37). Bogani et al. (38) linked radiomics features to molecular/genomic analyses to classify prognosis. In the present study, five different classifiers were used to predict the MSI status of EC based on MP-MRI. The RF classifier showed the best performance with AUC values of 0.844 and 0.897 in the training and validation cohorts, respectively. The RF is composed of many decision trees, and their prediction results are averaged by all the tree predictions, and thus effectively avoided overfitting. At the same time, we found that among the remaining features after dimensionality reduction, ADC_original_firstorder_Skewness and ADC_original_firstorder_Kurtosis of first-order features were significantly correlated with MSI status, which was consistent with the results of the studies by Fan et al. (39) and Pernicka et al. (40). The results of their studies showed that the MSI status was associated with kurtosis and intensity histograms. Our results showed the potential value of MP-MRI radiomics features for assessing genetic information of EC, although the underlying mechanism for radiomics reflecting MSI status remained unclear, we speculated that radiomics may represent tumor heterogeneity and thus predict genetic alterations (41, 42). Previous studies have reported that EC patients with MSI status are commonly associated with higher tumor grade, deeper myometrium invasion, and a higher incidence of LNM (43, 44). These pathological features suggested that the heterogeneity of MSI tumors may be higher than that of MSS tumors histologically, which could be captured on imaging using radiomics.

LVSI is the main histopathologic criterion for higher-risk EC, and radiomics method allows for a more comprehensive assessment of LVSI in EC patients. For detecting LVSI, our radiomics and combined models both obtained satisfactory performances. Compared to prior studies (18, 45, 46), our study had the highest AUC values in the training (AUC: radiomics, 0.952; combined, 0.959) and validation cohorts (AUC: radiomics, 0.908; combined, 0.926). Meanwhile, our study constructed radiomics models using five different classifiers (logistic regression, RF, decision tree, KNN, and Bayes) to choose the best classifier, which was more comprehensive and rational. About assessing the DMI of EC, we integrated the radscore and clinical independent risk factors into the combined model with the satisfactory performances (AUC=0.883 and 0.803, respectively) in the both cohorts. A previous study constructed the RF classifier for prediction of DMI (47), which achieved an AUC of 0.940 in the testing cohort. However, the RF classifier only obtained an AUC of 0.840 in our study. Because of the small number of samples and single sequence in the previous study, which made the radiomics model less generalizable. Other studies (26, 48, 49) obtained a DMI prediction performance with AUCs of 0.680, 0.810, and 0.790, respectively, from their testing datasets, which were lower than or closer to our results.

Comparison with non-radiomics-based efficacy prediction models, for example, one study (50) to investigate amide proton transfer weighting (APTw) imaging combined with intravoxel incoherent motion (IVIM) in the assessment of MSI in EC, obtaining a higher level of effectiveness(AUC = 0.973) than the present study. Meanwhile, another study (51) aimed to compare the value of DWI, diffusion kurtosis imaging (DKI), and APTw imaging in the assessment of risk stratification factors for stage I EC including histological subtype, grade, stage, and LVSI, accepting optimal predictive performance (AUC = 0.906). However, the small sample size of these study (n=34; n= 72) resulted in a low generalisability of the model and did not combine general clinical data to provide a comprehensive assessment of biological characteristics in EC.

There are several limitations in the current study. Firstly, owing to the relatively small number of cases in this single-center retrospective study, it is necessary to expand the sample size and include prospective data from multiple centers to test the generalizability of the prediction models. Secondly, our radiomics models are only based on plain MRI sequences (T_2_WI and DWI sequences, and ADC map). Incorporating more functional MRI sequences may improve the predictive performance of the model, which is worth future exploration. Finally, radiologists performed the tumor volume segmentation layer-by-layer, which is time-consuming and labor-intensive. Thus, a fully automatic and high-precision tumor segmentation method should be explored to replace manual segmentation.

## Conclusion

This study associated the radiomics features of MP-MRI with four biological characteristics (DMI, LVSI, MSI, and Her-2) related to the aggressiveness of EC. The established comprehensive models could predict more critical biological characteristics of EC and achieve promising prediction abilities. Therefore, they may be useful for the risk stratification of EC and provide valuable guidance for clinical decision-making.

## Data availability statement

The original contributions presented in the study are included in the article/**Supplementary Material**. Further inquiries can be directed to the corresponding author.

## Ethics statement

The studies involving humans were approved by The First Affiliated Hospital of Dalian Medical University. The studies were conducted in accordance with the local legislation and institutional requirements. Written informed consent for participation was not required from the participants or the participants’ legal guardians/next of kin in accordance with the national legislation and institutional requirements. Written informed consent was obtained from the individual(s) for the publication of any potentially identifiable images or data included in this article.

## Author contributions

CM: Data curation, Formal analysis, Investigation, Methodology, Writing – original draft, Writing – review & editing. YZ: Data curation, Investigation, Methodology, Writing – review & editing. QLS: Data curation, Investigation, Resources, Writing – review & editing. XM: Data curation, Investigation, Methodology, Writing – review & editing. QX: Data curation, Investigation, Writing – review & editing. ST: Data curation, Investigation, Methodology, Supervision, Writing – review & editing. LC: Data curation, Investigation, Writing – review & editing. NW: Investigation, Writing – review & editing. QWS: Data curation, Methodology, Writing – review & editing. LL: Data curation, Investigation, Writing – review & editing. JW: Data curation, Investigation, Writing – review & editing. AL: Conceptualization, Formal analysis, Investigation, Methodology, Supervision, Writing – review & editing.
