# Supplementary material for: Multi-parametric MRI-based radiomics for preoperative prediction of multiple biological characteristics in endometrial cancer
Source: Front Oncol. 2023 Dec 15;13:1280022. doi: 10.3389/fonc.2023.1280022 (PMC10768555; doi:10.3389/fonc.2023.1280022)
Supplement: Supplementary file 1 [file DataSheet_1.docx]

- **Supplementary Data S1. Main Imaging Parameters for MR Sequences**

|  | Sequences | TR  （ms） | TE  （ms） | Matrix  （mm） | NEX | FOV  （mm） | Thickness/  gap（mm） | Scanning  duration |
| --- | --- | --- | --- | --- | --- | --- | --- | --- |
| 1.5T MR GE | Sagittal T_2_WI | 3980 | 91.8 | 256×224 | 3.0 | 30×30 | 5.0/1.0 | 2min 55s |
|  | Axial T_2_WI | 5660 | 88.4 | 288×224 | 3.0 | 30×30 | 5.0/1.0 | 3min 13s |
|  | Axial DWI | 3725 | 71.1 | 128×128 | 6.0 | 30×30 | 5.0/1.0 | 1min 15s |
| 3.0T MR Philips | Sagittal T_2_WI | 3363 | 87.0 | 312×312 | 1.0 | 250×250 | 4.0/1.0 | 1min 27s |
|  | Axial T_2_WI | 3672 | 95.0 | 344×344 | 1.0 | 240×240 | 4.0/1.0 | 1min 02s |
|  | Axial DWI | 6000 | 55.0 | 80×78 | 1.0 | 240×240 | 4.0/1.0 | 1min 30s |

T_1_W= T1-weighted; T_2_W= T2-weighted；TR= time of repetition; TE=echo time; Fov=field of view; DWI=diffusion-weighted imaging.

- **Supplementary Data S2: Detailed name and description of radiomics features**

**2.1 Histogram features**

Histogram parameters are concerned with properties of individual pixels. They describe the distribution of voxel intensities within the MR image through commonly used and basic metrics. Let $X$ denote the three dimensional image matrix with $N$ voxels, and let $P$ denote the first order histogram divided by $N_{l}$ discrete intensity levels.

**1) Min Intensity:** The minimum intensity value of $X$.

**2) Max Intensity:** The maximum intensity value of $X$.

**3) Median Intensity:** The median intensity value of $X$.

## **4) Mean:**


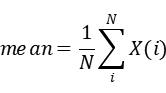


1. **Standard Deviation:**


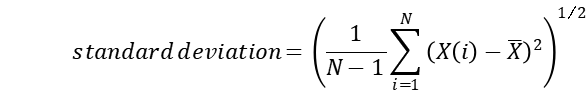


Where is the mean of .


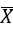

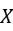


## **6) Mean Deviation:** The mean of the absolute deviations of all voxel intensities around the mean intensity value.

## **7) Relative Deviation:** Let $\overline{X}$ denote the mean of a set of quantities $X_{i}$, then the relative deviation is defined by:


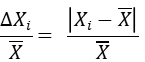


## **8) Variance:**

Where $\overline{X}$ is the mean of $X$.


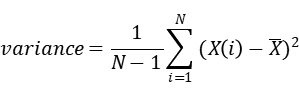


## **9) Range:** The range of intensity values of $X$.

## **10) Volume Count:** Describe the size of the ROI.

## **11) Voxel Value Sum:** Represents the Sum calculations for voxels in the ROI.

## **12) Root Mean Square (RMS):**


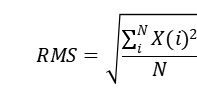


## **13) Skewness:**

Where is the mean of .


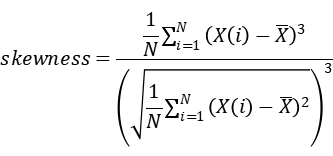

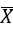

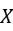


**14) Kurtosis:**

Where $\overline{X}$ is the mean of $X$.


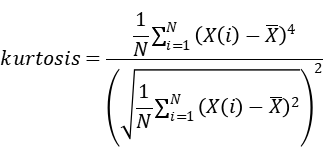


## **15) Uniformity:**


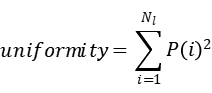


## **16) Energy:**


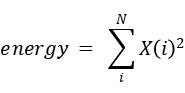


**17) Entropy:**


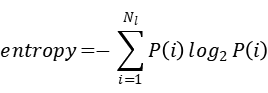


## **18) Frequency Size:** The ratio of the frequency of each object to the total frequency.

## **19) Percentile:** The percentile (p%) is defined as that value of the brightness $a$:


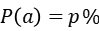


or equivalently:


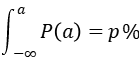


The P-th percentile of a list of N ordered values (sorted from least to greatest) is the smallest value in the list such that P percent of the data is less than or equal to that value. This is obtained by first calculating the ordinal rank and then taking the value from the ordered list that corresponds to that rank. The ordinal rank n is calculated using this formula:


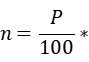


The 19 Percentiles are extracted: Percentile5, Percentile10, Percentile15, Percentile20, Percentile25, Percentile30, Percentile35, Percentile40, Percentile45, Percentile50, Percentile55, Percentile60, Percentile65, Percentile70, Percentile75, Percentile80, Percentile85, Percentile90, Percentile95.

**20) Quantile:** For a finite population of *N* equally probable values indexed 1, …, *N* from lowest to highest, the k-th q-quantile of this population can equivalently be computed via the value of:


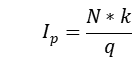


The 5 Quantiles are extracted: Quantile0.025, Quantile0.25, Quantile0.5, Quantile0.75, Quantile0.975.

**2.2 Grey level co-occurrence matrix (GLCM) features**

The grey level co-occurrence matrix (GLCM) represents the joint probability of certain sets of pixels having certain grey-level values. It calculates how many times a pixel with grey-level i occurs jointly with another pixel having a grey value j. By varying the displacement vector d between each pair of pixels.


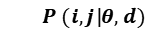


The rotation angle of an offset: 0°, 45°, 90°, 135° and displacement vectors (distance to the neighbor pixel: 1, 2, 3 ...), different co-occurrence distributions from the same image of reference. GLCM of an image is computed using displacement vector d defined by its radius, (distance or count to the next adjacent neighbor preferably is equal to one) and rotational angles.

### GLCM Energy:

### Formula:


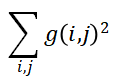


g is a GLCM

Where i,j are the spatial coordinates of g (i,j).

*Angle=All Direction, 0, 45, 90, 135, All_SD;

*Offset=1, 4, 7

The 18 features of GLCM Energy are extracted:

GLCMEnergy_AllDirection_offset1, GLCMEnergy_AllDirection_offset1_SD, GLCMEnergy_angle0_offset1, GLCMEnergy_angle45_offset1,

GLCMEnergy_angle90_offset1, GLCMEnergy_angle135_offset1,

GLCMEnergy_AllDirection_offset4, GLCMEnergy_AllDirection_offset4_SD, GLCMEnergy_angle0_offset4, GLCMEnergy_angle45_offset4,

GLCMEnergy_angle90_offset4, GLCMEnergy_angle135_offset4,

GLCMEnergy_AllDirection_offset7, GLCMEnergy_angle0_offset7, GLCMEnergy_angle45_offset7, GLCMEnergy_angle90_offset7, GLCMEnergy_angle135_offset7, GLCMEnergy_AllDirection_offset7_SD

### 2) GLCM Entropy:

Formula:


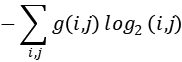


*Angle=All Direction, 0, 45, 90, 135, All_SD;

*Offset=1, 4, 7

The 18 features of GLCM Entropy are extracted:

GLCMEntropy_AllDirection_offset1, GLCMEntropy_AllDirection_offset1_SD, GLCMEntropy_angle0_offset1, GLCMEntropy_angle45_offset1, GLCMEntropy_angle90_offset1, GLCMEntropy_angle135_offset1,

GLCMEntropy_AllDirection_offset4, GLCMEntropy_AllDirection_offset4_SD, GLCMEntropy_angle0_offset4, GLCMEntropy_angle45_offset4, GLCMEntropy_angle90_offset4, GLCMEntropy_angle135_offset4,

GLCMEntropy_AllDirection_offset7, GLCMEntropy_AllDirection_offset7_SD, GLCMEntropy_angle0_offset7, GLCMEntropy_angle45_offset7, GLCMEntropy_angle90_offset7, GLCMEntropy_angle135_offset7

### 3) GLCM Inertia:

Formula:


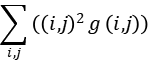


*Angle=All Direction, 0, 45, 90, 135, All_SD;

*Offset=1, 4, 7

The 18 features of GLCM Inertia are extracted:

Inertia_AllDirection_offset1, Inertia _AllDirection_offset1_SD,

Inertia _angle0_offset1, Inertia _angle45_offset1,

Inertia _angle90_offset1, Inertia _angle135_offset1,

Inertia _AllDirection_offset4, Inertia _AllDirection_offset4_SD,

Inertia _angle0_offset4, Inertia _angle45_offset4,

Inertia _angle90_offset4, Inertia _angle135_offset4,

Inertia _AllDirection_offset7, Inertia _AllDirection_offset7_SD,

Inertia _angle0_offset7, Inertia _angle45_offset7,

Inertia _angle90_offset7, Inertia _angle135_offset7

### 4) Correlation:

Formula:


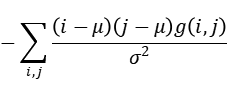


*Angle=All Direction, 0, 45, 90, 135, All_SD;

*Offset=1, 4, 7

The 18 features of Correlation are extracted:

Correlation_AllDirection_offset1, Correlation _AllDirection_offset1_SD,

Correlation _angle0_offset1, Correlation _angle45_offset1,

Correlation _angle90_offset1, Correlation _angle135_offset1,

Correlation _AllDirection_offset4, Correlation _AllDirection_offset4_SD,

Correlation _angle0_offset4, Correlation _angle45_offset4,

Correlation _angle90_offset4, Correlation _angle135_offset4,

Correlation _AllDirection_offset7, Correlation _AllDirection_offset7_SD,

Correlation _angle0_offset7, Correlation _angle45_offset7,

Correlation _angle90_offset7, Correlation _angle135_offset7

### 5) Inverse Difference Moment:

Formula:


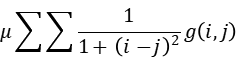


*Angle=All Direction, 0, 45, 90, 135, All_SD;

*Offset=1, 4, 7

The 18 features of Inverse Difference Moment are extracted:

InverseDifferenceMoment_AllDirection_offset1, InverseDifferenceMoment_AllDirection_offset1_SD,

InverseDifferenceMoment_angle0_offset1,

InverseDifferenceMoment_angle45_offset1,

InverseDifferenceMoment_angle90_offset1,

InverseDifferenceMoment_angle135_offset1,

InverseDifferenceMoment_AllDirection_offset4,

InverseDifferenceMoment_AllDirection_offset4_SD,

InverseDifferenceMoment_angle0_offset4,

InverseDifferenceMoment_angle45_offset4,

InverseDifferenceMoment_angle90_offset4,

InverseDifferenceMoment_angle135_offset4,

InverseDifferenceMoment_AllDirection_offset7,

InverseDifferenceMoment_AllDirection_offset7_SD,

InverseDifferenceMoment_angle0_offset7,

InverseDifferenceMoment_angle45_offset7,

InverseDifferenceMoment_angle90_offset7,

InverseDifferenceMoment_angle135_offset7

### 6) Cluster Shade:

Formula:


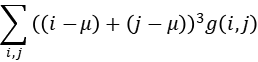


*Angle=All Direction, 0, 45, 90, 135, All_SD;

*Offset=1, 4, 7

The 18 features of Cluster Shade are extracted:

ClusterShade_AllDirection_offset1, ClusterShade_AllDirection_offset1_SD,

ClusterShade_angle0_offset1, ClusterShade_angle45_offset1,

ClusterShade_angle90_offset1, ClusterShade_angle135_offset1,

ClusterShade_AllDirection_offset4, ClusterShade_AllDirection_offset4_SD,

ClusterShade_angle0_offset4, ClusterShade_angle45_offset4,

ClusterShade_angle90_offset4, ClusterShade_angle135_offset4,

ClusterShade_AllDirection_offset7, ClusterShade_AllDirection_offset7_SD,

ClusterShade_angle0_offset7, ClusterShade_angle45_offset7,

ClusterShade_angle90_offset7, ClusterShade_angle135_offset7

### 7) Cluster Prominence:

Formula:


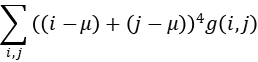


*Angle=All Direction, 0, 45, 90, 135, All_SD;

*Offset=1, 4, 7

The 18 features of Cluster Prominence are extracted:

ClusterProminence_AllDirection_offset1, ClusterProminence_AllDirection_offset1_SD,

ClusterProminence_angle0_offset1, ClusterProminence_angle45_offset1,

ClusterProminence_angle90_offset1, ClusterProminence_angle135_offset1,

ClusterProminence_AllDirection_offset4, ClusterProminence_AllDirection_offset4_SD,

ClusterProminence_angle0_offset4, ClusterProminence_angle45_offset4,

ClusterProminence_angle90_offset4, ClusterProminence_angle135_offset4,

ClusterProminence_AllDirection_offset7, ClusterProminence_AllDirection_offset7_SD,

ClusterProminence_angle0_offset7, ClusterProminence_angle45_offset7,

ClusterProminence_angle90_offset7, ClusterProminence_angle135_offset7

### 8) Haralick Correlation:

Formula:


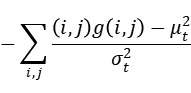


where $\mu_{t}$ and $\sigma_{t}$ are the mean and standard deviation of the row (or column, due to

symmetry) sums.

*Angle=All Direction, 0, 45, 90, 135, All_SD;

*Offset=1, 4, 7

The 18 features of Haralick Correlation are extracted:

HaralickCorrelation_AllDirection_offset1, HaralickCorrelation_AllDirection_offset1_SD,

HaralickCorrelation_angle0_offset1, HaralickCorrelation_angle45_offset1,

HaralickCorrelation_angle90_offset1, HaralickCorrelation_angle135_offset1,

HaralickCorrelation_AllDirection_offset4, HaralickCorrelation_AllDirection_offset4_SD,

HaralickCorrelation_angle0_offset4, HaralickCorrelation_angle45_offset4,

HaralickCorrelation_angle90_offset4, HaralickCorrelation_angle135_offset4,

HaralickCorrelation_AllDirection_offset7, HaralickCorrelation_AllDirection_offset7_SD,

HaralickCorrelation_angle0_offset7, HaralickCorrelation_angle45_offset7,

HaralickCorrelation_angle90_offset7, HaralickCorrelation_angle135_offset7

**2.3 Grey level run length matrix (GLRLM) features**

The grey level run-length matrix (RLM) **P_r_ (i**, **j | θ**) is defined as the numbers of runs with pixels of grey level *i* and run length *j* for a given direction θ. RLMs is generated for each sample image segment having directions (0°,45°,90° &135°), then the following ten statistical features were derived.

1. **Short Run Emphasis：**

$$SRE(\theta)=\frac{1}{n_{r}}\sum_{i=1}^{M} \sum_{j=1}^{N} \frac{p(i,j,\theta)}{j^{2}}$$

*Angle=All Direction, 0, 45, 90, 135, All_SD;

*Offset=1, 4, 7

The 18 features of Short Run Emphasis are extracted:

ShortRunEmphasis_AllDirection_offset1, ShortRunEmphasis_AllDirection_offset1_SD,

ShortRunEmphasis_angle0_offset1, ShortRunEmphasis_angle45_offset1,

ShortRunEmphasis_angle90_offset1, ShortRunEmphasis_angle135_offset1,

ShortRunEmphasis_AllDirection_offset4, ShortRunEmphasis_AllDirection_offset4_SD,

ShortRunEmphasis_angle0_offset4, ShortRunEmphasis_angle45_offset4,

ShortRunEmphasis_angle90_offset4, ShortRunEmphasis_angle135_offset4,

ShortRunEmphasis_AllDirection_offset7, ShortRunEmphasis_AllDirection_offset7_SD,

ShortRunEmphasis_angle0_offset7, ShortRunEmphasis_angle45_offset7,

ShortRunEmphasis_angle90_offset7, ShortRunEmphasis_angle135_offset7

1. **Long Run Emphasis：**

$$LRE(\theta)=\frac{1}{n_{r}}\sum_{i=1}^{M} \sum_{j=1}^{N} p(i,j,\theta)j^{2}$$

*Angle=All Direction, 0, 45, 90, 135, All_SD;

*Offset=1, 4, 7

The 18 features of Long Run Emphasis are extracted:

LongRunEmphasis_AllDirection_offset1, LongRunEmphasis_AllDirection_offset1_SD,

LongRunEmphasis_angle0_offset1, LongRunEmphasis_angle45_offset1,

LongRunEmphasis_angle90_offset1, LongRunEmphasis_angle135_offset1,

LongRunEmphasis_AllDirection_offset4, LongRunEmphasis_AllDirection_offset4_SD,

LongRunEmphasis_angle0_offset4, LongRunEmphasis_angle45_offset4,

LongRunEmphasis_angle90_offset4, LongRunEmphasis_angle135_offset4,

LongRunEmphasis_AllDirection_offset7, LongRunEmphasis_AllDirection_offset7_SD,

LongRunEmphasis_angle0_offset7, LongRunEmphasis_angle45_offset7,

LongRunEmphasis_angle90_offset7, LongRunEmphasis_angle135_offset7

1. **Grey Level Nonuniformity：**

$$GLN(\theta)=\frac{1}{n_{r}}\sum_{i=1}^{M} (\sum_{j=1}^{N} p(i,j,\theta))^{2}$$

*Angle=All Direction, 0, 45, 90, 135, All_SD;

*Offset=1, 4, 7

The 18 features of Grey Level Nonuniformity are extracted:

GreyLevelNonuniformity_AllDirection_offset1, GreyLevelNonuniformity_AllDirection_offset1_SD,

GreyLevelNonuniformity_angle0_offset1, GreyLevelNonuniformity_angle45_offset1,

GreyLevelNonuniformity_angle90_offset1, GreyLevelNonuniformity_angle135_offset1,

GreyLevelNonuniformity_AllDirection_offset4, GreyLevelNonuniformity_AllDirection_offset4_SD,

GreyLevelNonuniformity_angle0_offset4, GreyLevelNonuniformity_angle45_offset4,

GreyLevelNonuniformity_angle90_offset4, GreyLevelNonuniformity_angle135_offset4,

GreyLevelNonuniformity_AllDirection_offset7, GreyLevelNonuniformity_AllDirection_offset7_SD,

GreyLevelNonuniformity_angle0_offset7, GreyLevelNonuniformity_angle45_offset7,

GreyLevelNonuniformity_angle90_offset7, GreyLevelNonuniformity_angle135_offset7

1. **Run Length Nonuniformity：**

$$RLN(\theta)=\frac{1}{n_{r}}\sum_{j=i}^{N} (\sum_{i=1}^{M} p(i,j,\theta))^{2}$$

*Angle=All Direction, 0, 45, 90, 135, All_SD;

*Offset=1, 4, 7

The 18 features of Run Length Nonuniformity are extracted:

RunLengthNonuniformity_AllDirection_offset1, RunLengthNonuniformity_AllDirection_offset1_SD,

RunLengthNonuniformity_angle0_offset1, RunLengthNonuniformity_angle45_offset1,

RunLengthNonuniformity_angle90_offset1, RunLengthNonuniformity_angle135_offset1,

RunLengthNonuniformity_AllDirection_offset4, RunLengthNonuniformity_AllDirection_offset4_SD,

RunLengthNonuniformity_angle0_offset4, RunLengthNonuniformity_angle45_offset4,

RunLengthNonuniformity_angle90_offset4, RunLengthNonuniformity_angle135_offset4,

RunLengthNonuniformity_AllDirection_offset7, RunLengthNonuniformity_AllDirection_offset7_SD,

RunLengthNonuniformity_angle0_offset7, RunLengthNonuniformity_angle45_offset7,

RunLengthNonuniformity_angle90_offset7, RunLengthNonuniformity_angle135_offset7

1. **Low Grey Level Run Emphasis：**

$$LGRE(\theta)=\frac{1}{n_{r}}\sum_{j=i}^{N} \sum_{i=1}^{M} \frac{p(i,j,\theta)}{i^{2}}$$

*Angle=All Direction, 0, 45, 90, 135, All_SD;

*Offset=1, 4, 7

The 18 features of LowGreyLevelRunEmphasis are extracted:

LowGreyLevelRunEmphasis_AllDirection_offset1, LowGreyLevelRunEmphasis_AllDirection_offset1_SD,

LowGreyLevelRunEmphasis_angle0_offset1, LowGreyLevelRunEmphasis_angle45_offset1,

LowGreyLevelRunEmphasis_angle90_offset1, LowGreyLevelRunEmphasis_angle135_offset1,

LowGreyLevelRunEmphasis_AllDirection_offset4, LowGreyLevelRunEmphasis_AllDirection_offset4_SD,

LowGreyLevelRunEmphasis_angle0_offset4, LowGreyLevelRunEmphasis_angle45_offset4,

LowGreyLevelRunEmphasis_angle90_offset4, LowGreyLevelRunEmphasis_angle135_offset4,

LowGreyLevelRunEmphasis_AllDirection_offset7, LowGreyLevelRunEmphasis_AllDirection_offset7_SD,

LowGreyLevelRunEmphasis_angle0_offset7, LowGreyLevelRunEmphasis_angle45_offset7,

LowGreyLevelRunEmphasis_angle90_offset7, LowGreyLevelRunEmphasis_angle135_offset7

1. **High Grey Level Run Emphasis：**

$$HGRE(\theta)=\frac{1}{n_{r}}\sum_{j=i}^{N} \sum_{i=1}^{M} p(i,j,\theta)i^{2}$$

*Angle=All Direction, 0, 45, 90, 135, All_SD;

*Offset=1, 4, 7

The 18 features of HighGreyLevelRunEmphasis are extracted:

HighGreyLevelRunEmphasis_AllDirection_offset1, HighGreyLevelRunEmphasis_AllDirection_offset1_SD,

HighGreyLevelRunEmphasis_angle0_offset1, HighGreyLevelRunEmphasis_angle45_offset1,

HighGreyLevelRunEmphasis_angle90_offset1, HighGreyLevelRunEmphasis_angle135_offset1,

HighGreyLevelRunEmphasis_AllDirection_offset4, HighGreyLevelRunEmphasis_AllDirection_offset4_SD,

HighGreyLevelRunEmphasis_angle0_offset4, HighGreyLevelRunEmphasis_angle45_offset4,

HighGreyLevelRunEmphasis_angle90_offset4, HighGreyLevelRunEmphasis_angle135_offset4,

HighGreyLevelRunEmphasis_AllDirection_offset7, HighGreyLevelRunEmphasis_AllDirection_offset7_SD,

HighGreyLevelRunEmphasis_angle0_offset7, HighGreyLevelRunEmphasis_angle45_offset7,

HighGreyLevelRunEmphasis_angle90_offset7, HighGreyLevelRunEmphasis_angle135_offset7

1. **Short Run Low Grey Level Emphasis：**

$$SRLGE(\theta)=\frac{1}{n_{r}}\sum_{j=i}^{N} \sum_{i=1}^{M} \frac{p(i,j,\theta)}{i^{2}j^{2}}$$

*Angle=All Direction, 0, 45, 90, 135, All_SD;

*Offset=1, 4, 7

The 18 features of ShortRunLowGreyLevelEmphasis are extracted:

ShortRunLowGreyLevelEmphasis_AllDirection_offset1, ShortRunLowGreyLevelEmphasis_AllDirection_offset1_SD,

ShortRunLowGreyLevelEmphasis_angle0_offset1, ShortRunLowGreyLevelEmphasis_angle45_offset1,

ShortRunLowGreyLevelEmphasis_angle90_offset1, ShortRunLowGreyLevelEmphasis_angle135_offset1,

ShortRunLowGreyLevelEmphasis_AllDirection_offset4, ShortRunLowGreyLevelEmphasis_AllDirection_offset4_SD,

ShortRunLowGreyLevelEmphasis_angle0_offset4, ShortRunLowGreyLevelEmphasis_angle45_offset4,

ShortRunLowGreyLevelEmphasis_angle90_offset4, ShortRunLowGreyLevelEmphasis_angle135_offset4,

ShortRunLowGreyLevelEmphasis_AllDirection_offset7, ShortRunLowGreyLevelEmphasis_AllDirection_offset7_SD,

ShortRunLowGreyLevelEmphasis_angle0_offset7, ShortRunLowGreyLevelEmphasis_angle45_offset7,

ShortRunLowGreyLevelEmphasis_angle90_offset7, ShortRunLowGreyLevelEmphasis_angle135_offset7

1. **ShortRunHighGreyLevelEmphasis：**

$$SRHGE(\theta)=\frac{1}{n_{r}}\sum_{j=i}^{N} \sum_{i=1}^{M} \frac{p(i,j,\theta)i^{2}}{j^{2}}$$

*Angle=All Direction, 0, 45, 90, 135, All_SD;

*Offset=1, 4, 7

The 18 features of ShortRunHighGreyLevelEmphasis are extracted:

ShortRunHighGreyLevelEmphasis_AllDirection_offset1, ShortRunHighGreyLevelEmphasis_AllDirection_offset1_SD,

ShortRunHighGreyLevelEmphasis_angle0_offset1, ShortRunHighGreyLevelEmphasis_angle45_offset1,

ShortRunHighGreyLevelEmphasis_angle90_offset1, ShortRunHighGreyLevelEmphasis_angle135_offset1,

ShortRunHighGreyLevelEmphasis_AllDirection_offset4, ShortRunHighGreyLevelEmphasis_AllDirection_offset4_SD,

ShortRunHighGreyLevelEmphasis_angle0_offset4, ShortRunHighGreyLevelEmphasis_angle45_offset4,

ShortRunHighGreyLevelEmphasis_angle90_offset4, ShortRunHighGreyLevelEmphasis_angle135_offset4,

ShortRunHighGreyLevelEmphasis_AllDirection_offset7, ShortRunHighGreyLevelEmphasis_AllDirection_offset7_SD,

ShortRunHighGreyLevelEmphasis_angle0_offset7, ShortRunHighGreyLevelEmphasis_angle45_offset7,

ShortRunHighGreyLevelEmphasis_angle90_offset7, ShortRunHighGreyLevelEmphasis_angle135_offset7

1. **LongRunLowGreyLevelEmphasis:**

$$LRLGE(\theta)=\frac{1}{n_{r}}\sum_{j=i}^{N} \sum_{i=1}^{M} \frac{p(i,j,\theta)j^{2}}{i^{2}}$$

*Angle=All Direction, 0, 45, 90, 135, All_SD;

*Offset=1, 4, 7

The 18 features of LongRunLowGreyLevelEmphasis are extracted:

LongRunLowGreyLevelEmphasis_AllDirection_offset1, LongRunLowGreyLevelEmphasis_AllDirection_offset1_SD,

LongRunLowGreyLevelEmphasis_angle0_offset1, LongRunLowGreyLevelEmphasis_angle45_offset1,

LongRunLowGreyLevelEmphasis_angle90_offset1, LongRunLowGreyLevelEmphasis_angle135_offset1,

LongRunLowGreyLevelEmphasis_AllDirection_offset4, LongRunLowGreyLevelEmphasis_AllDirection_offset4_SD,

LongRunLowGreyLevelEmphasis_angle0_offset4, LongRunLowGreyLevelEmphasis_angle45_offset4,

LongRunLowGreyLevelEmphasis_angle90_offset4, LongRunLowGreyLevelEmphasis_angle135_offset4,

LongRunLowGreyLevelEmphasis_AllDirection_offset7, LongRunLowGreyLevelEmphasis_AllDirection_offset7_SD,

LongRunLowGreyLevelEmphasis_angle0_offset7, LongRunLowGreyLevelEmphasis_angle45_offset7,

LongRunLowGreyLevelEmphasis_angle90_offset7, LongRunLowGreyLevelEmphasis_angle135_offset7

1. **LongRunHighGreyLevelEmphasis:**

$$LRHGE(\theta)=\frac{1}{n_{r}}\sum_{j=i}^{N} \sum_{i=1}^{M} p(i,j,\theta)i^{2}j^{2}$$

where *n_r_* is the total number of runs and *n_p_* is the number of pixels in the image.

*Angle=All Direction, 0, 45, 90, 135, All_SD;

*Offset=1, 4, 7

The 18 features of LongRunHighGreyLevelEmphasis are extracted:

LongRunHighGreyLevelEmphasis_AllDirection_offset1, LongRunHighGreyLevelEmphasis_AllDirection_offset1_SD,

LongRunHighGreyLevelEmphasis_angle0_offset1, LongRunHighGreyLevelEmphasis_angle45_offset1,

LongRunHighGreyLevelEmphasis_angle90_offset1, LongRunHighGreyLevelEmphasis_angle135_offset1,

LongRunHighGreyLevelEmphasis_AllDirection_offset4, LongRunHighGreyLevelEmphasis_AllDirection_offset4_SD,

LongRunHighGreyLevelEmphasis_angle0_offset4, LongRunHighGreyLevelEmphasis_angle45_offset4,

LongRunHighGreyLevelEmphasis_angle90_offset4, LongRunHighGreyLevelEmphasis_angle135_offset4,

LongRunHighGreyLevelEmphasis_AllDirection_offset7, LongRunHighGreyLevelEmphasis_AllDirection_offset7_SD,

LongRunHighGreyLevelEmphasis_angle0_offset7, LongRunHighGreyLevelEmphasis_angle45_offset7,

LongRunHighGreyLevelEmphasis_angle90_offset7, LongRunHighGreyLevelEmphasis_angle135_offset7

**2.4 Grey-level zone size matrix (GLZSM) features**

The grey level Size Zone Matrix (SZM) is the starting point of Thibault matrices. For a texture image f with N grey levels, it is denoted GSf(s, g) and provides a statistical representation by the estimation of a bivariate conditional probability density function of the image distribution values. It is calculated according to the pioneering Run Length Matrix principle: the value of the matrix GSf(s, g) is equal to the number of zones of size s and of grey level g. The resulting matrix has a fixed number of lines equal to N, the number of grey levels, and a dynamic number of columns, determined by the size of the largest zone as well as the size quantization.

The more homogeneous the texture, the wider and flatter the matrix. SZM does not required computation in several directions. However, it has been empirically proved that the degree of grey level quantization still has an important impact on the texture classification performance.

Let P define the GLSZM of a quantized volume V (x,y,z) with isotropic voxel size. *P(i,j)* represents the number of 3D zones of grey-levels *i* and of size j in V , *Ng* represents the pre-defined number of quantized grey-levels set in V, and *Lz* represents the size of the largest zone (of any grey-level) in V. One GLSZM of size *Ng*× *Lz* is computed per volume V by adding up all possible largest zone-sizes, with zones constructed from 26-connected neighbours of the same grey-level in 3D space (one voxel can be part of only one zone). The entry *(i,j)* of the normalized GLSZM is then defined as:


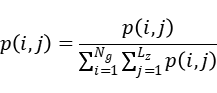


The following quantities are also defined:


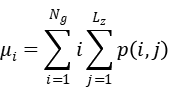

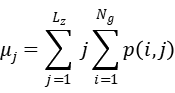


## **1) SmallAreaEmphasis:**


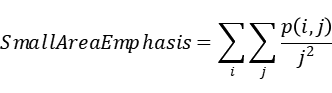


## **2) LargeAreaEmphasis:**


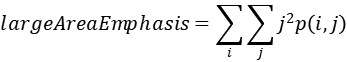


## **3) IntensityVariability:**


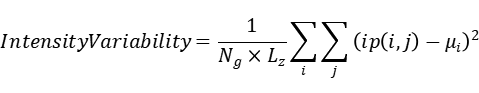


## **4) SizeZoneVariability:**


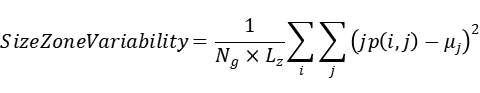


## **5) ZonePercentage**


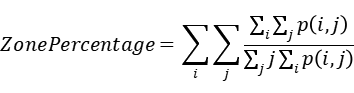


## **6) LowIntensityEmphasis:**


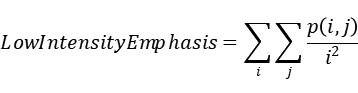


## **7) HighIntensityEmphasis:**


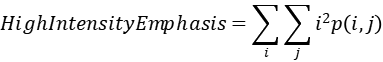


## **8) LowIntensitySmallAreaEmphasis:**


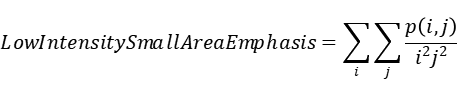


## **9) HighIntensitySmallAreaEmphasis:**


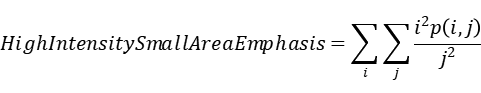


**10) LowIntensityLargeAreaEmphasis:**


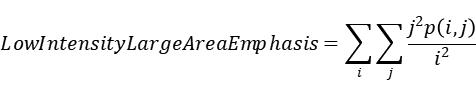


## **HighIntensityLargeAreaEmphasis:**


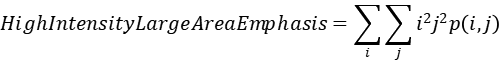


**2.5 Haralick features**

### 1) HaraEntropy:

Formula:


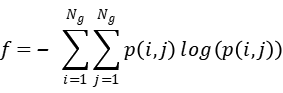


where $\mu_{t}$ and $\sigma_{t}$ are the mean and standard deviation of the row (or column, due to symmetry) sums.

### 2) Angular Second Moment:

Formula:


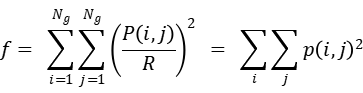


### 3) Contrast:

Formula:


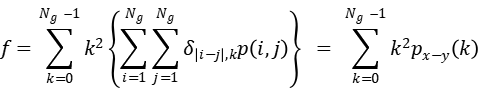


### 4) Haralick Variance:

Formula:


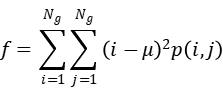


### 5) SumAverage:

Formula:


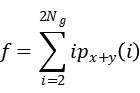


### 6) SumVariance:

Formula:


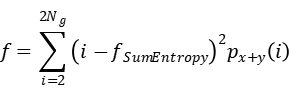


### 7) SumEntropy:

Formula:


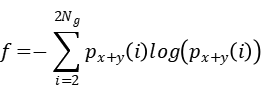


### 8) Difference Variance:

Formula:


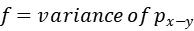


### 9) Difference Entropy:

Formula:


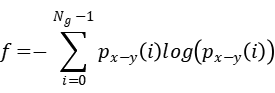


### 10) Inverse Difference Moment:

Formula:


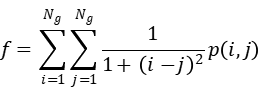


**2.6 Form factors**

These group of features includes descriptors of the three-dimensional size and shape of the tumor region. Let in the following definitions *V* denote the volume and *A* the surface area of the volume of interest. We determined the following shape and sizebased features:

**1) MeshVolume:**

Formula:

**
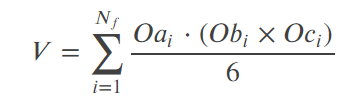
**

The volume of the ROI *V* is calculated from the triangle mesh of the ROI. For each face *i* in the mesh, defined by points *a_i_*, *b_i_* and *c_i_*, the (signed) volume *V_f_* of the tetrahedron defined by that face and the origin of the image (*O*) is calculated.

**2) OneVoxelVolume:** The volume of a single voxel.

**3) VoxelVolume:** The volume (*V*) of the tumor is determined by counting the number of pixels in the tumor region and multiplying this value by the voxel size.

**4) Surface Area:**


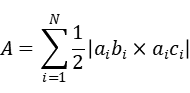


**5) Surface to Volume Ratio:**


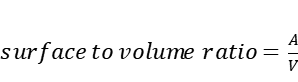


**6) Sphericity:**


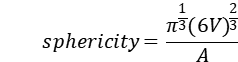


**7) Compactness 1:**


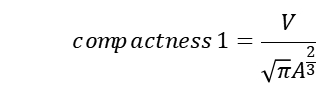


**8) Compactness 2:**


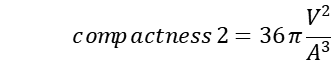


**9) Spherical Disproportion:**


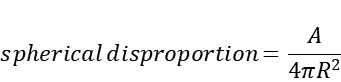


Where *R* is the radius of a sphere with the same volume as the tumor.

Where *N* is the total number of triangles covering the surface and *a*, *b* and *c* are edge vectors of the triangles.

**10) Maximum 3D diameter:** The maximum three-dimensional tumor diameter is measured as the largest pairwise Euclidean distance, between voxels on the surface of the tumor volume.

**11) MajorAxisLength:**

**
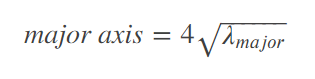
**

This feature yield the largest axis length of the ROI-enclosing ellipsoid and is calculated using the largest principal component *λ_major_*.

**12) MinorAxisLength:**

**
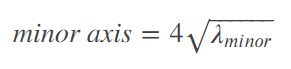
**

This feature yield the second-largest axis length of the ROI-enclosing ellipsoid and is calculated using the largest principal component *λ_minor_*.

**13) LeastAxisLength:**

**
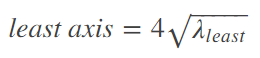
**

This feature yield the smallest axis length of the ROI-enclosing ellipsoid and is calculated using the largest principal component *λ_least_*.

**14) Elongation:**

**
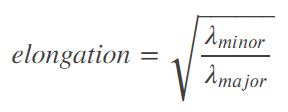
**

Here, *λ_major_* and *λ_minor_* are the lengths of the largest and second largest principal component axes.

**15) Flatness:**

**
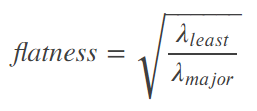
**

Here, *λ_major_* and *λ_least_* are the lengths of the largest and smallest principal component axes. The values range between 1 (non-flat, sphere-like) and 0 (a flat object, or single-slice segmentation).

**2.7 Gaussian transform features**

The method of image filter processing is Laplacian of Gaussian (LoG). LoG filter is an edge enhancement filter, which emphasizes areas of grey level change, where sigma defines how coarse the emphasized texture should be. A low sigma emphasis on fine textures (change over a short distance), where a high sigma value emphasizes coarse textures (grey level change over a large distance). One sigma value was used in this study (1 mm).

- **Supplement data S3:** The parameters used in the construction of MP-MRI models

| Classifiers | Parameters | MSI | Her-2 | DMI | LVSI |
| --- | --- | --- | --- | --- | --- |
| Logistic regression | 11_ratio | 0.05 | 0.05 | 0.05 | 0.05 |
| Random forest | n_estimators | 10 | 10 | 10 | 10 |
|  | max _ depth | 3 | 3 | 3 | 3 |
|  | min_ samples_split | 5 | 5 | 5 | 5 |
| Decision tree | max _ depth | 5 | 3 | 5 | 5 |
|  | min_ samples_split | 10 | 5 | 10 | 10 |
| K-nearest neighbor | n_neighbors | 10 | 10 | 5 | 10 |
| Bayes | Model | GaussianNB | GaussianNB | GaussianNB | GaussianNB |

**Supplement data S4：**Details of ICC values with intra- and intre-observer reproducibility.

1. **ICC_intra_T_2_WI**

| parameters | Icc_value |
| --- | --- |
| lbp-3D-k_firstorder_10Percentile | 0.98664227 |
| lbp-3D-k_firstorder_90Percentile | 0.96934014 |
| lbp-3D-k_firstorder_Energy | 0.99851822 |
| lbp-3D-k_firstorder_Entropy | 0.96670051 |
| lbp-3D-k_firstorder_InterquartileRange | 0.97436408 |
| lbp-3D-k_firstorder_Kurtosis | 0.98286681 |
| lbp-3D-k_firstorder_Maximum | 0.97756396 |
| lbp-3D-k_firstorder_Mean | 0.98171216 |
| lbp-3D-k_firstorder_MeanAbsoluteDeviation | 0.97967478 |
| lbp-3D-k_firstorder_Median | 0.98462183 |
| lbp-3D-k_firstorder_Minimum | 0.95649643 |
| lbp-3D-k_firstorder_Range | 0.97808869 |
| lbp-3D-k_firstorder_RobustMeanAbsoluteDeviation | 0.97654956 |
| lbp-3D-k_firstorder_RootMeanSquared | 0.98867448 |
| lbp-3D-k_firstorder_Skewness | 0.98182153 |
| lbp-3D-k_firstorder_TotalEnergy | 0.99759405 |
| lbp-3D-k_firstorder_Uniformity | 0.96905384 |
| lbp-3D-k_firstorder_Variance | 0.98789937 |
| lbp-3D-k_glcm_Autocorrelation | 0.96657592 |
| lbp-3D-k_glcm_ClusterProminence | 0.94385751 |
| lbp-3D-k_glcm_ClusterShade | 0.94641665 |
| lbp-3D-k_glcm_ClusterTendency | 0.96133872 |
| lbp-3D-k_glcm_Contrast | 0.97038786 |
| lbp-3D-k_glcm_Correlation | 0.78340421 |
| lbp-3D-k_glcm_DifferenceAverage | 0.97038786 |
| lbp-3D-k_glcm_DifferenceEntropy | 0.96706175 |
| lbp-3D-k_glcm_DifferenceVariance | 0.9677495 |
| lbp-3D-k_glcm_Id | 0.97038786 |
| lbp-3D-k_glcm_Idm | 0.97038786 |
| lbp-3D-k_glcm_Idmn | 0.97038785 |
| lbp-3D-k_glcm_Idn | 0.97038786 |
| lbp-3D-k_glcm_Imc1 | 0.86490709 |
| lbp-3D-k_glcm_Imc2 | 0.91664633 |
| lbp-3D-k_glcm_InverseVariance | 0.97038786 |
| lbp-3D-k_glcm_JointAverage | 0.96757494 |
| lbp-3D-k_glcm_JointEnergy | 0.96642433 |
| lbp-3D-k_glcm_JointEntropy | 0.96419067 |
| lbp-3D-k_glcm_MCC | 0.83634676 |
| lbp-3D-k_glcm_MaximumProbability | 0.96881981 |
| lbp-3D-k_glcm_SumAverage | 0.96757494 |
| lbp-3D-k_glcm_SumEntropy | 0.96248773 |
| lbp-3D-k_glcm_SumSquares | 0.96563326 |
| lbp-3D-k_gldm_DependenceEntropy | 0.94564251 |
| lbp-3D-k_gldm_DependenceNonUniformity | 0.99838656 |
| lbp-3D-k_gldm_DependenceNonUniformityNormalized | 0.94213873 |
| lbp-3D-k_gldm_DependenceVariance | 0.9425173 |
| lbp-3D-k_gldm_GrayLevelNonUniformity | 0.99865258 |
| lbp-3D-k_gldm_GrayLevelVariance | 0.96905384 |
| lbp-3D-k_gldm_HighGrayLevelEmphasis | 0.97053009 |
| lbp-3D-k_gldm_LargeDependenceEmphasis | 0.9784376 |
| lbp-3D-k_gldm_LargeDependenceHighGrayLevelEmphasis | 0.97924752 |
| lbp-3D-k_gldm_LargeDependenceLowGrayLevelEmphasis | 0.97823093 |
| lbp-3D-k_gldm_LowGrayLevelEmphasis | 0.97053009 |
| lbp-3D-k_gldm_SmallDependenceEmphasis | 0.94468202 |
| lbp-3D-k_gldm_SmallDependenceHighGrayLevelEmphasis | 0.94014727 |
| lbp-3D-k_gldm_SmallDependenceLowGrayLevelEmphasis | 0.96022979 |
| lbp-3D-k_glrlm_GrayLevelNonUniformity | 0.99737558 |
| lbp-3D-k_glrlm_GrayLevelNonUniformityNormalized | 0.97114903 |
| lbp-3D-k_glrlm_GrayLevelVariance | 0.97114903 |
| lbp-3D-k_glrlm_HighGrayLevelRunEmphasis | 0.9746052 |
| lbp-3D-k_glrlm_LongRunEmphasis | 0.97772697 |
| lbp-3D-k_glrlm_LongRunHighGrayLevelEmphasis | 0.97787486 |
| lbp-3D-k_glrlm_LongRunLowGrayLevelEmphasis | 0.97769211 |
| lbp-3D-k_glrlm_LowGrayLevelRunEmphasis | 0.9746052 |
| lbp-3D-k_glrlm_RunEntropy | 0.98416379 |
| lbp-3D-k_glrlm_RunLengthNonUniformity | 0.99158912 |
| lbp-3D-k_glrlm_RunLengthNonUniformityNormalized | 0.95157959 |
| lbp-3D-k_glrlm_RunPercentage | 0.97732926 |
| lbp-3D-k_glrlm_RunVariance | 0.98043833 |
| lbp-3D-k_glrlm_ShortRunEmphasis | 0.93705251 |
| lbp-3D-k_glrlm_ShortRunHighGrayLevelEmphasis | 0.96240755 |
| lbp-3D-k_glrlm_ShortRunLowGrayLevelEmphasis | 0.95459225 |
| lbp-3D-k_glszm_GrayLevelNonUniformity | 0.99812543 |
| lbp-3D-k_glszm_GrayLevelNonUniformityNormalized | 0.85174992 |
| lbp-3D-k_glszm_GrayLevelVariance | 0.85174992 |
| lbp-3D-k_glszm_HighGrayLevelZoneEmphasis | 0.8362155 |
| lbp-3D-k_glszm_LargeAreaEmphasis | 0.99653491 |
| lbp-3D-k_glszm_LargeAreaHighGrayLevelEmphasis | 0.99652343 |
| lbp-3D-k_glszm_LargeAreaLowGrayLevelEmphasis | 0.9965377 |
| lbp-3D-k_glszm_LowGrayLevelZoneEmphasis | 0.8362155 |
| lbp-3D-k_glszm_SizeZoneNonUniformity | 0.99549972 |
| lbp-3D-k_glszm_SizeZoneNonUniformityNormalized | 0.81294083 |
| lbp-3D-k_glszm_SmallAreaEmphasis | 0.76450806 |
| lbp-3D-k_glszm_SmallAreaHighGrayLevelEmphasis | 0.79225236 |
| lbp-3D-k_glszm_SmallAreaLowGrayLevelEmphasis | 0.62633613 |
| lbp-3D-k_glszm_ZoneEntropy | 0.88542463 |
| lbp-3D-k_glszm_ZonePercentage | 0.95121655 |
| lbp-3D-k_glszm_ZoneVariance | 0.9968824 |
| lbp-3D-k_ngtdm_Busyness | 0.9985051 |
| lbp-3D-k_ngtdm_Coarseness | 0.80345576 |
| lbp-3D-k_ngtdm_Complexity | 0.97532798 |
| lbp-3D-k_ngtdm_Contrast | 0.97905313 |
| lbp-3D-k_ngtdm_Strength | 0.80931017 |
| lbp-3D-m1_firstorder_10Percentile | 0.90707669 |
| lbp-3D-m1_firstorder_90Percentile | 0.96652221 |
| lbp-3D-m1_firstorder_Energy | 0.99865595 |
| lbp-3D-m1_firstorder_Entropy | -34.416667 |
| lbp-3D-m1_firstorder_InterquartileRange | 0.97276904 |
| lbp-3D-m1_firstorder_Kurtosis | 0.97762326 |
| lbp-3D-m1_firstorder_Maximum | 0.70038911 |
| lbp-3D-m1_firstorder_Mean | 0.97169509 |
| lbp-3D-m1_firstorder_MeanAbsoluteDeviation | 0.9870269 |
| lbp-3D-m1_firstorder_Median | 0.94724089 |
| lbp-3D-m1_firstorder_Range | 0.70038911 |
| lbp-3D-m1_firstorder_RobustMeanAbsoluteDeviation | 0.97717781 |
| lbp-3D-m1_firstorder_RootMeanSquared | 0.97491846 |
| lbp-3D-m1_firstorder_Skewness | 0.95084795 |
| lbp-3D-m1_firstorder_TotalEnergy | 0.99771234 |
| lbp-3D-m1_firstorder_Uniformity | #NAME? |
| lbp-3D-m1_firstorder_Variance | 0.9770554 |
| lbp-3D-m1_glcm_Autocorrelation | #NAME? |
| lbp-3D-m1_glcm_Correlation | #NAME? |
| lbp-3D-m1_glcm_Id | #NAME? |
| lbp-3D-m1_glcm_Idm | #NAME? |
| lbp-3D-m1_glcm_Idmn | #NAME? |
| lbp-3D-m1_glcm_Idn | #NAME? |
| lbp-3D-m1_glcm_JointAverage | #NAME? |
| lbp-3D-m1_glcm_JointEnergy | #NAME? |
| lbp-3D-m1_glcm_MCC | #NAME? |
| lbp-3D-m1_glcm_MaximumProbability | #NAME? |
| lbp-3D-m1_glcm_SumAverage | #NAME? |
| lbp-3D-m1_gldm_DependenceEntropy | 0.92904628 |
| lbp-3D-m1_gldm_DependenceNonUniformity | 0.99879432 |
| lbp-3D-m1_gldm_DependenceNonUniformityNormalized | 0.92512238 |
| lbp-3D-m1_gldm_DependenceVariance | 0.87626281 |
| lbp-3D-m1_gldm_GrayLevelNonUniformity | 0.99865595 |
| lbp-3D-m1_gldm_HighGrayLevelEmphasis | #NAME? |
| lbp-3D-m1_gldm_LargeDependenceEmphasis | 0.98419801 |
| lbp-3D-m1_gldm_LargeDependenceHighGrayLevelEmphasis | 0.98419801 |
| lbp-3D-m1_gldm_LargeDependenceLowGrayLevelEmphasis | 0.98419801 |
| lbp-3D-m1_gldm_LowGrayLevelEmphasis | #NAME? |
| lbp-3D-m1_gldm_SmallDependenceEmphasis | 0.97317142 |
| lbp-3D-m1_gldm_SmallDependenceHighGrayLevelEmphasis | 0.97317142 |
| lbp-3D-m1_gldm_SmallDependenceLowGrayLevelEmphasis | 0.97317142 |
| lbp-3D-m1_glrlm_GrayLevelNonUniformity | 0.99671898 |
| lbp-3D-m1_glrlm_GrayLevelNonUniformityNormalized | #NAME? |
| lbp-3D-m1_glrlm_HighGrayLevelRunEmphasis | #NAME? |
| lbp-3D-m1_glrlm_LongRunEmphasis | 0.99342275 |
| lbp-3D-m1_glrlm_LongRunHighGrayLevelEmphasis | 0.99342275 |
| lbp-3D-m1_glrlm_LongRunLowGrayLevelEmphasis | 0.99342275 |
| lbp-3D-m1_glrlm_LowGrayLevelRunEmphasis | #NAME? |
| lbp-3D-m1_glrlm_RunEntropy | 0.98655992 |
| lbp-3D-m1_glrlm_RunLengthNonUniformity | 0.97178256 |
| lbp-3D-m1_glrlm_RunLengthNonUniformityNormalized | 0.94993856 |
| lbp-3D-m1_glrlm_RunPercentage | 0.98353618 |
| lbp-3D-m1_glrlm_RunVariance | 0.99461833 |
| lbp-3D-m1_glrlm_ShortRunEmphasis | 0.96218344 |
| lbp-3D-m1_glrlm_ShortRunHighGrayLevelEmphasis | 0.96218344 |
| lbp-3D-m1_glrlm_ShortRunLowGrayLevelEmphasis | 0.96218344 |
| lbp-3D-m1_glszm_GrayLevelNonUniformity | #NAME? |
| lbp-3D-m1_glszm_GrayLevelNonUniformityNormalized | #NAME? |
| lbp-3D-m1_glszm_HighGrayLevelZoneEmphasis | #NAME? |
| lbp-3D-m1_glszm_LargeAreaEmphasis | 0.99921406 |
| lbp-3D-m1_glszm_LargeAreaHighGrayLevelEmphasis | 0.99921406 |
| lbp-3D-m1_glszm_LargeAreaLowGrayLevelEmphasis | 0.99921406 |
| lbp-3D-m1_glszm_LowGrayLevelZoneEmphasis | #NAME? |
| lbp-3D-m1_glszm_SizeZoneNonUniformity | #NAME? |
| lbp-3D-m1_glszm_SizeZoneNonUniformityNormalized | #NAME? |
| lbp-3D-m1_glszm_SmallAreaEmphasis | 0.95314294 |
| lbp-3D-m1_glszm_SmallAreaHighGrayLevelEmphasis | 0.95314294 |
| lbp-3D-m1_glszm_SmallAreaLowGrayLevelEmphasis | 0.95314294 |
| lbp-3D-m1_glszm_ZonePercentage | 0.97662952 |
| lbp-3D-m1_ngtdm_Coarseness | #NAME? |
| lbp-3D-m2_firstorder_10Percentile | 0.94565939 |
| lbp-3D-m2_firstorder_90Percentile | 0.98483639 |
| lbp-3D-m2_firstorder_Energy | 0.99850224 |
| lbp-3D-m2_firstorder_InterquartileRange | 0.95803461 |
| lbp-3D-m2_firstorder_Kurtosis | 0.94840373 |
| lbp-3D-m2_firstorder_Maximum | 0.85059581 |
| lbp-3D-m2_firstorder_Mean | 0.96480608 |
| lbp-3D-m2_firstorder_MeanAbsoluteDeviation | 0.96530649 |
| lbp-3D-m2_firstorder_Median | 0.95958599 |
| lbp-3D-m2_firstorder_Range | 0.85059581 |
| lbp-3D-m2_firstorder_RobustMeanAbsoluteDeviation | 0.96509033 |
| lbp-3D-m2_firstorder_RootMeanSquared | 0.96877354 |
| lbp-3D-m2_firstorder_Skewness | 0.95626344 |
| lbp-3D-m2_firstorder_TotalEnergy | 0.99762516 |
| lbp-3D-m2_firstorder_Uniformity | #NAME? |
| lbp-3D-m2_firstorder_Variance | 0.96741893 |
| lbp-3D-m2_glcm_Autocorrelation | #NAME? |
| lbp-3D-m2_glcm_Correlation | #NAME? |
| lbp-3D-m2_glcm_Id | #NAME? |
| lbp-3D-m2_glcm_Idm | #NAME? |
| lbp-3D-m2_glcm_Idmn | #NAME? |
| lbp-3D-m2_glcm_Idn | #NAME? |
| lbp-3D-m2_glcm_JointAverage | #NAME? |
| lbp-3D-m2_glcm_JointEnergy | #NAME? |
| lbp-3D-m2_glcm_MCC | #NAME? |
| lbp-3D-m2_glcm_MaximumProbability | #NAME? |
| lbp-3D-m2_glcm_SumAverage | #NAME? |
| lbp-3D-m2_gldm_DependenceEntropy | 0.92904628 |
| lbp-3D-m2_gldm_DependenceNonUniformity | 0.99879432 |
| lbp-3D-m2_gldm_DependenceNonUniformityNormalized | 0.92512238 |
| lbp-3D-m2_gldm_DependenceVariance | 0.87626281 |
| lbp-3D-m2_gldm_GrayLevelNonUniformity | 0.99865595 |
| lbp-3D-m2_gldm_HighGrayLevelEmphasis | #NAME? |
| lbp-3D-m2_gldm_LargeDependenceEmphasis | 0.98419801 |
| lbp-3D-m2_gldm_LargeDependenceHighGrayLevelEmphasis | 0.98419801 |
| lbp-3D-m2_gldm_LargeDependenceLowGrayLevelEmphasis | 0.98419801 |
| lbp-3D-m2_gldm_LowGrayLevelEmphasis | #NAME? |
| lbp-3D-m2_gldm_SmallDependenceEmphasis | 0.97317142 |
| lbp-3D-m2_gldm_SmallDependenceHighGrayLevelEmphasis | 0.97317142 |
| lbp-3D-m2_gldm_SmallDependenceLowGrayLevelEmphasis | 0.97317142 |
| lbp-3D-m2_glrlm_GrayLevelNonUniformity | 0.99671898 |
| lbp-3D-m2_glrlm_GrayLevelNonUniformityNormalized | #NAME? |
| lbp-3D-m2_glrlm_HighGrayLevelRunEmphasis | #NAME? |
| lbp-3D-m2_glrlm_LongRunEmphasis | 0.99342275 |
| lbp-3D-m2_glrlm_LongRunHighGrayLevelEmphasis | 0.99342275 |
| lbp-3D-m2_glrlm_LongRunLowGrayLevelEmphasis | 0.99342275 |
| lbp-3D-m2_glrlm_LowGrayLevelRunEmphasis | #NAME? |
| lbp-3D-m2_glrlm_RunEntropy | 0.98655992 |
| lbp-3D-m2_glrlm_RunLengthNonUniformity | 0.97178256 |
| lbp-3D-m2_glrlm_RunLengthNonUniformityNormalized | 0.94993856 |
| lbp-3D-m2_glrlm_RunPercentage | 0.98353618 |
| lbp-3D-m2_glrlm_RunVariance | 0.99461833 |
| lbp-3D-m2_glrlm_ShortRunEmphasis | 0.96218344 |
| lbp-3D-m2_glrlm_ShortRunHighGrayLevelEmphasis | 0.96218344 |
| lbp-3D-m2_glrlm_ShortRunLowGrayLevelEmphasis | 0.96218344 |
| lbp-3D-m2_glszm_GrayLevelNonUniformity | #NAME? |
| lbp-3D-m2_glszm_GrayLevelNonUniformityNormalized | #NAME? |
| lbp-3D-m2_glszm_HighGrayLevelZoneEmphasis | #NAME? |
| lbp-3D-m2_glszm_LargeAreaEmphasis | 0.99921406 |
| lbp-3D-m2_glszm_LargeAreaHighGrayLevelEmphasis | 0.99921406 |
| lbp-3D-m2_glszm_LargeAreaLowGrayLevelEmphasis | 0.99921406 |
| lbp-3D-m2_glszm_LowGrayLevelZoneEmphasis | #NAME? |
| lbp-3D-m2_glszm_SizeZoneNonUniformity | #NAME? |
| lbp-3D-m2_glszm_SizeZoneNonUniformityNormalized | #NAME? |
| lbp-3D-m2_glszm_SmallAreaEmphasis | 0.95314294 |
| lbp-3D-m2_glszm_SmallAreaHighGrayLevelEmphasis | 0.95314294 |
| lbp-3D-m2_glszm_SmallAreaLowGrayLevelEmphasis | 0.95314294 |
| lbp-3D-m2_glszm_ZonePercentage | 0.97662952 |
| lbp-3D-m2_ngtdm_Coarseness | #NAME? |
| original_firstorder_10Percentile | 0.99730554 |
| original_firstorder_90Percentile | 0.99789973 |
| original_firstorder_Energy | 0.99982192 |
| original_firstorder_Entropy | 0.99745038 |
| original_firstorder_InterquartileRange | 0.9874043 |
| original_firstorder_Kurtosis | 0.97057889 |
| original_firstorder_Maximum | 0.99954178 |
| original_firstorder_Mean | 0.99908019 |
| original_firstorder_MeanAbsoluteDeviation | 0.99468338 |
| original_firstorder_Median | 0.99925233 |
| original_firstorder_Minimum | 0.9919392 |
| original_firstorder_Range | 0.99638136 |
| original_firstorder_RobustMeanAbsoluteDeviation | 0.98727987 |
| original_firstorder_RootMeanSquared | 0.99906848 |
| original_firstorder_Skewness | 0.986216 |
| original_firstorder_TotalEnergy | 0.99972533 |
| original_firstorder_Uniformity | 0.99768574 |
| original_firstorder_Variance | 0.99448349 |
| original_glcm_Autocorrelation | 0.97466669 |
| original_glcm_ClusterProminence | 0.99945592 |
| original_glcm_ClusterShade | 0.99982283 |
| original_glcm_ClusterTendency | 0.99552325 |
| original_glcm_Contrast | 0.98449308 |
| original_glcm_Correlation | 0.97070897 |
| original_glcm_DifferenceAverage | 0.99014783 |
| original_glcm_DifferenceEntropy | 0.99599877 |
| original_glcm_DifferenceVariance | 0.99275116 |
| original_glcm_Id | 0.99639155 |
| original_glcm_Idm | 0.99656336 |
| original_glcm_Idmn | 0.92855156 |
| original_glcm_Idn | 0.9606553 |
| original_glcm_Imc1 | 0.98562624 |
| original_glcm_Imc2 | 0.99272831 |
| original_glcm_InverseVariance | 0.99403513 |
| original_glcm_JointAverage | 0.97700709 |
| original_glcm_JointEnergy | 0.99821202 |
| original_glcm_JointEntropy | 0.996773 |
| original_glcm_MCC | 0.96162559 |
| original_glcm_MaximumProbability | 0.99650378 |
| original_glcm_SumAverage | 0.97700709 |
| original_glcm_SumEntropy | 0.99674816 |
| original_glcm_SumSquares | 0.99401345 |
| original_gldm_DependenceEntropy | 0.99623971 |
| original_gldm_DependenceNonUniformity | 0.99836574 |
| original_gldm_DependenceNonUniformityNormalized | 0.99507842 |
| original_gldm_DependenceVariance | 0.99591425 |
| original_gldm_GrayLevelNonUniformity | 0.99874982 |
| original_gldm_GrayLevelVariance | 0.99445986 |
| original_gldm_HighGrayLevelEmphasis | 0.97683725 |
| original_gldm_LargeDependenceEmphasis | 0.99764413 |
| original_gldm_LargeDependenceHighGrayLevelEmphasis | 0.98029399 |
| original_gldm_LargeDependenceLowGrayLevelEmphasis | 0.99923743 |
| original_gldm_LowGrayLevelEmphasis | 0.9752216 |
| original_gldm_SmallDependenceEmphasis | 0.99477299 |
| original_gldm_SmallDependenceHighGrayLevelEmphasis | 0.98375925 |
| original_gldm_SmallDependenceLowGrayLevelEmphasis | 0.84264858 |
| original_glrlm_GrayLevelNonUniformity | 0.99858342 |
| original_glrlm_GrayLevelNonUniformityNormalized | 0.99768541 |
| original_glrlm_GrayLevelVariance | 0.99503456 |
| original_glrlm_HighGrayLevelRunEmphasis | 0.97726816 |
| original_glrlm_LongRunEmphasis | 0.99855601 |
| original_glrlm_LongRunHighGrayLevelEmphasis | 0.97267181 |
| original_glrlm_LongRunLowGrayLevelEmphasis | 0.99863797 |
| original_glrlm_LowGrayLevelRunEmphasis | 0.97680898 |
| original_glrlm_RunEntropy | 0.99698448 |
| original_glrlm_RunLengthNonUniformity | 0.99840384 |
| original_glrlm_RunLengthNonUniformityNormalized | 0.99620719 |
| original_glrlm_RunPercentage | 0.99709751 |
| original_glrlm_RunVariance | 0.99881977 |
| original_glrlm_ShortRunEmphasis | 0.99676057 |
| original_glrlm_ShortRunHighGrayLevelEmphasis | 0.97806817 |
| original_glrlm_ShortRunLowGrayLevelEmphasis | 0.95791399 |
| original_glszm_GrayLevelNonUniformity | 0.9975782 |
| original_glszm_GrayLevelNonUniformityNormalized | 0.99743627 |
| original_glszm_GrayLevelVariance | 0.99817346 |
| original_glszm_HighGrayLevelZoneEmphasis | 0.98382584 |
| original_glszm_LargeAreaEmphasis | 0.99737283 |
| original_glszm_LargeAreaHighGrayLevelEmphasis | 0.99338578 |
| original_glszm_LargeAreaLowGrayLevelEmphasis | 0.9978156 |
| original_glszm_LowGrayLevelZoneEmphasis | 0.98600202 |
| original_glszm_SizeZoneNonUniformity | 0.99663047 |
| original_glszm_SizeZoneNonUniformityNormalized | 0.99051457 |
| original_glszm_SmallAreaEmphasis | 0.98871786 |
| original_glszm_SmallAreaHighGrayLevelEmphasis | 0.98691964 |
| original_glszm_SmallAreaLowGrayLevelEmphasis | 0.95727628 |
| original_glszm_ZoneEntropy | 0.98998546 |
| original_glszm_ZonePercentage | 0.99399614 |
| original_glszm_ZoneVariance | 0.9973711 |
| original_ngtdm_Busyness | 0.99775692 |
| original_ngtdm_Coarseness | 0.94670888 |
| original_ngtdm_Complexity | 0.99679619 |
| original_ngtdm_Contrast | 0.9715927 |
| original_ngtdm_Strength | 0.99347729 |
| original_shape_Elongation | 0.97601787 |
| original_shape_Flatness | 0.94084549 |
| original_shape_LeastAxisLength | 0.99216191 |
| original_shape_MajorAxisLength | 0.99503161 |
| original_shape_Maximum2DDiameterColumn | 0.98812313 |
| original_shape_Maximum2DDiameterRow | 0.99550668 |
| original_shape_Maximum2DDiameterSlice | 0.98996961 |
| original_shape_Maximum3DDiameter | 0.99565708 |
| original_shape_MeshVolume | 0.99777762 |
| original_shape_MinorAxisLength | 0.99595194 |
| original_shape_Sphericity | 0.88059921 |
| original_shape_SurfaceArea | 0.99714523 |
| original_shape_SurfaceVolumeRatio | 0.97442963 |
| original_shape_VoxelVolume | 0.99777767 |

1. **ICC_intra_DWI**

| parameters | Icc_value |
| --- | --- |
| lbp-3D-k_firstorder_10Percentile | 0.98397369 |
| lbp-3D-k_firstorder_90Percentile | 0.93951526 |
| lbp-3D-k_firstorder_Energy | 0.98832365 |
| lbp-3D-k_firstorder_Entropy | 0.9199487 |
| lbp-3D-k_firstorder_InterquartileRange | 0.95699929 |
| lbp-3D-k_firstorder_Kurtosis | 0.98705148 |
| lbp-3D-k_firstorder_Maximum | 0.99169709 |
| lbp-3D-k_firstorder_Mean | 0.98218433 |
| lbp-3D-k_firstorder_MeanAbsoluteDeviation | 0.94653969 |
| lbp-3D-k_firstorder_Median | 0.98468324 |
| lbp-3D-k_firstorder_Minimum | 0.86129325 |
| lbp-3D-k_firstorder_Range | 0.9922472 |
| lbp-3D-k_firstorder_RobustMeanAbsoluteDeviation | 0.95534243 |
| lbp-3D-k_firstorder_RootMeanSquared | 0.98940785 |
| lbp-3D-k_firstorder_Skewness | 0.9757373 |
| lbp-3D-k_firstorder_TotalEnergy | 0.98900784 |
| lbp-3D-k_firstorder_Uniformity | 0.91483154 |
| lbp-3D-k_firstorder_Variance | 0.94791966 |
| lbp-3D-k_glcm_Autocorrelation | 0.87826575 |
| lbp-3D-k_glcm_ClusterProminence | 0.80326802 |
| lbp-3D-k_glcm_ClusterShade | 0.82855025 |
| lbp-3D-k_glcm_ClusterTendency | 0.86878417 |
| lbp-3D-k_glcm_Contrast | 0.91539429 |
| lbp-3D-k_glcm_Correlation | 0.67108595 |
| lbp-3D-k_glcm_DifferenceAverage | 0.91539429 |
| lbp-3D-k_glcm_DifferenceEntropy | 0.91102219 |
| lbp-3D-k_glcm_DifferenceVariance | 0.91486379 |
| lbp-3D-k_glcm_Id | 0.91539429 |
| lbp-3D-k_glcm_Idm | 0.91539429 |
| lbp-3D-k_glcm_Idmn | 0.9153943 |
| lbp-3D-k_glcm_Idn | 0.91539429 |
| lbp-3D-k_glcm_Imc1 | 0.86955936 |
| lbp-3D-k_glcm_Imc2 | 0.78290028 |
| lbp-3D-k_glcm_InverseVariance | 0.91539429 |
| lbp-3D-k_glcm_JointAverage | 0.8889715 |
| lbp-3D-k_glcm_JointEnergy | 0.90206656 |
| lbp-3D-k_glcm_JointEntropy | 0.89611459 |
| lbp-3D-k_glcm_MCC | 0.78208857 |
| lbp-3D-k_glcm_MaximumProbability | 0.90137446 |
| lbp-3D-k_glcm_SumAverage | 0.8889715 |
| lbp-3D-k_glcm_SumEntropy | 0.89043191 |
| lbp-3D-k_glcm_SumSquares | 0.89184417 |
| lbp-3D-k_gldm_DependenceEntropy | 0.97792585 |
| lbp-3D-k_gldm_DependenceNonUniformity | 0.98814591 |
| lbp-3D-k_gldm_DependenceNonUniformityNormalized | 0.97346129 |
| lbp-3D-k_gldm_DependenceVariance | 0.95803644 |
| lbp-3D-k_gldm_GrayLevelNonUniformity | 0.98586127 |
| lbp-3D-k_gldm_GrayLevelVariance | 0.91483154 |
| lbp-3D-k_gldm_HighGrayLevelEmphasis | 0.90978881 |
| lbp-3D-k_gldm_LargeDependenceEmphasis | 0.97620798 |
| lbp-3D-k_gldm_LargeDependenceHighGrayLevelEmphasis | 0.97892823 |
| lbp-3D-k_gldm_LargeDependenceLowGrayLevelEmphasis | 0.97546393 |
| lbp-3D-k_gldm_LowGrayLevelEmphasis | 0.90978881 |
| lbp-3D-k_gldm_SmallDependenceEmphasis | 0.9329249 |
| lbp-3D-k_gldm_SmallDependenceHighGrayLevelEmphasis | 0.91002996 |
| lbp-3D-k_gldm_SmallDependenceLowGrayLevelEmphasis | 0.95873731 |
| lbp-3D-k_glrlm_GrayLevelNonUniformity | 0.98804014 |
| lbp-3D-k_glrlm_GrayLevelNonUniformityNormalized | 0.95618376 |
| lbp-3D-k_glrlm_GrayLevelVariance | 0.95618376 |
| lbp-3D-k_glrlm_HighGrayLevelRunEmphasis | 0.95947237 |
| lbp-3D-k_glrlm_LongRunEmphasis | 0.99093061 |
| lbp-3D-k_glrlm_LongRunHighGrayLevelEmphasis | 0.99131518 |
| lbp-3D-k_glrlm_LongRunLowGrayLevelEmphasis | 0.99082766 |
| lbp-3D-k_glrlm_LowGrayLevelRunEmphasis | 0.95947237 |
| lbp-3D-k_glrlm_RunEntropy | 0.99192601 |
| lbp-3D-k_glrlm_RunLengthNonUniformity | 0.98646042 |
| lbp-3D-k_glrlm_RunLengthNonUniformityNormalized | 0.95122084 |
| lbp-3D-k_glrlm_RunPercentage | 0.96780474 |
| lbp-3D-k_glrlm_RunVariance | 0.99125854 |
| lbp-3D-k_glrlm_ShortRunEmphasis | 0.93775058 |
| lbp-3D-k_glrlm_ShortRunHighGrayLevelEmphasis | 0.94449315 |
| lbp-3D-k_glrlm_ShortRunLowGrayLevelEmphasis | 0.95530362 |
| lbp-3D-k_glszm_GrayLevelNonUniformity | 0.98410971 |
| lbp-3D-k_glszm_GrayLevelNonUniformityNormalized | 0.89172567 |
| lbp-3D-k_glszm_GrayLevelVariance | 0.89172567 |
| lbp-3D-k_glszm_HighGrayLevelZoneEmphasis | 0.87285599 |
| lbp-3D-k_glszm_LargeAreaEmphasis | 0.97953378 |
| lbp-3D-k_glszm_LargeAreaHighGrayLevelEmphasis | 0.97984067 |
| lbp-3D-k_glszm_LargeAreaLowGrayLevelEmphasis | 0.979457 |
| lbp-3D-k_glszm_LowGrayLevelZoneEmphasis | 0.87285599 |
| lbp-3D-k_glszm_SizeZoneNonUniformity | 0.96733463 |
| lbp-3D-k_glszm_SizeZoneNonUniformityNormalized | 0.799918 |
| lbp-3D-k_glszm_SmallAreaEmphasis | 0.73843586 |
| lbp-3D-k_glszm_SmallAreaHighGrayLevelEmphasis | 0.73856454 |
| lbp-3D-k_glszm_SmallAreaLowGrayLevelEmphasis | 0.71685323 |
| lbp-3D-k_glszm_ZoneEntropy | 0.83667234 |
| lbp-3D-k_glszm_ZonePercentage | 0.94008776 |
| lbp-3D-k_glszm_ZoneVariance | 0.97909901 |
| lbp-3D-k_ngtdm_Busyness | 0.98911381 |
| lbp-3D-k_ngtdm_Coarseness | 0.86284297 |
| lbp-3D-k_ngtdm_Complexity | 0.9472467 |
| lbp-3D-k_ngtdm_Contrast | 0.9209923 |
| lbp-3D-k_ngtdm_Strength | 0.86815958 |
| lbp-3D-m1_firstorder_10Percentile | 0.69889761 |
| lbp-3D-m1_firstorder_90Percentile | 0.94274999 |
| lbp-3D-m1_firstorder_Energy | 0.98689272 |
| lbp-3D-m1_firstorder_InterquartileRange | 0.96374303 |
| lbp-3D-m1_firstorder_Kurtosis | 0.94798155 |
| lbp-3D-m1_firstorder_Maximum | 0.06020123 |
| lbp-3D-m1_firstorder_Mean | 0.95365085 |
| lbp-3D-m1_firstorder_MeanAbsoluteDeviation | 0.95959555 |
| lbp-3D-m1_firstorder_Median | 0.92167134 |
| lbp-3D-m1_firstorder_Range | 0.06020123 |
| lbp-3D-m1_firstorder_RobustMeanAbsoluteDeviation | 0.96513636 |
| lbp-3D-m1_firstorder_RootMeanSquared | 0.96092693 |
| lbp-3D-m1_firstorder_Skewness | 0.92541291 |
| lbp-3D-m1_firstorder_TotalEnergy | 0.98762857 |
| lbp-3D-m1_firstorder_Uniformity | #NAME? |
| lbp-3D-m1_firstorder_Variance | 0.94359253 |
| lbp-3D-m1_glcm_Autocorrelation | #NAME? |
| lbp-3D-m1_glcm_Correlation | #NAME? |
| lbp-3D-m1_glcm_Id | #NAME? |
| lbp-3D-m1_glcm_Idm | #NAME? |
| lbp-3D-m1_glcm_Idmn | #NAME? |
| lbp-3D-m1_glcm_Idn | #NAME? |
| lbp-3D-m1_glcm_JointAverage | #NAME? |
| lbp-3D-m1_glcm_JointEnergy | #NAME? |
| lbp-3D-m1_glcm_MCC | #NAME? |
| lbp-3D-m1_glcm_MaximumProbability | #NAME? |
| lbp-3D-m1_glcm_SumAverage | #NAME? |
| lbp-3D-m1_gldm_DependenceEntropy | 0.92658835 |
| lbp-3D-m1_gldm_DependenceNonUniformity | 0.98133386 |
| lbp-3D-m1_gldm_DependenceNonUniformityNormalized | 0.9392569 |
| lbp-3D-m1_gldm_DependenceVariance | 0.94536875 |
| lbp-3D-m1_gldm_GrayLevelNonUniformity | 0.98652295 |
| lbp-3D-m1_gldm_HighGrayLevelEmphasis | #NAME? |
| lbp-3D-m1_gldm_LargeDependenceEmphasis | 0.98452133 |
| lbp-3D-m1_gldm_LargeDependenceHighGrayLevelEmphasis | 0.98452133 |
| lbp-3D-m1_gldm_LargeDependenceLowGrayLevelEmphasis | 0.98452133 |
| lbp-3D-m1_gldm_LowGrayLevelEmphasis | #NAME? |
| lbp-3D-m1_gldm_SmallDependenceEmphasis | 0.95307307 |
| lbp-3D-m1_gldm_SmallDependenceHighGrayLevelEmphasis | 0.95307307 |
| lbp-3D-m1_gldm_SmallDependenceLowGrayLevelEmphasis | 0.95307307 |
| lbp-3D-m1_glrlm_GrayLevelNonUniformity | 0.99010031 |
| lbp-3D-m1_glrlm_GrayLevelNonUniformityNormalized | #NAME? |
| lbp-3D-m1_glrlm_HighGrayLevelRunEmphasis | #NAME? |
| lbp-3D-m1_glrlm_LongRunEmphasis | 0.98320851 |
| lbp-3D-m1_glrlm_LongRunHighGrayLevelEmphasis | 0.98320851 |
| lbp-3D-m1_glrlm_LongRunLowGrayLevelEmphasis | 0.98320851 |
| lbp-3D-m1_glrlm_LowGrayLevelRunEmphasis | #NAME? |
| lbp-3D-m1_glrlm_RunEntropy | 0.99305285 |
| lbp-3D-m1_glrlm_RunLengthNonUniformity | 0.98410975 |
| lbp-3D-m1_glrlm_RunLengthNonUniformityNormalized | 0.97209315 |
| lbp-3D-m1_glrlm_RunPercentage | 0.98012294 |
| lbp-3D-m1_glrlm_RunVariance | 0.97350107 |
| lbp-3D-m1_glrlm_ShortRunEmphasis | 0.95456885 |
| lbp-3D-m1_glrlm_ShortRunHighGrayLevelEmphasis | 0.95456885 |
| lbp-3D-m1_glrlm_ShortRunLowGrayLevelEmphasis | 0.95456885 |
| lbp-3D-m1_glszm_GrayLevelNonUniformity | 0.95206612 |
| lbp-3D-m1_glszm_GrayLevelNonUniformityNormalized | #NAME? |
| lbp-3D-m1_glszm_HighGrayLevelZoneEmphasis | #NAME? |
| lbp-3D-m1_glszm_LargeAreaEmphasis | 0.93662402 |
| lbp-3D-m1_glszm_LargeAreaHighGrayLevelEmphasis | 0.93662402 |
| lbp-3D-m1_glszm_LargeAreaLowGrayLevelEmphasis | 0.93662402 |
| lbp-3D-m1_glszm_LowGrayLevelZoneEmphasis | #NAME? |
| lbp-3D-m1_glszm_SizeZoneNonUniformity | #NAME? |
| lbp-3D-m1_glszm_SizeZoneNonUniformityNormalized | 0.99325111 |
| lbp-3D-m1_glszm_SmallAreaEmphasis | 0.77578763 |
| lbp-3D-m1_glszm_SmallAreaHighGrayLevelEmphasis | 0.77578763 |
| lbp-3D-m1_glszm_SmallAreaLowGrayLevelEmphasis | 0.77578763 |
| lbp-3D-m1_glszm_ZonePercentage | 0.90379519 |
| lbp-3D-m1_ngtdm_Coarseness | #NAME? |
| lbp-3D-m2_firstorder_10Percentile | 0.8131932 |
| lbp-3D-m2_firstorder_90Percentile | 0.84349535 |
| lbp-3D-m2_firstorder_Energy | 0.98779021 |
| lbp-3D-m2_firstorder_InterquartileRange | 0.9303448 |
| lbp-3D-m2_firstorder_Kurtosis | 0.93292044 |
| lbp-3D-m2_firstorder_Maximum | 0.79757962 |
| lbp-3D-m2_firstorder_Mean | 0.8949734 |
| lbp-3D-m2_firstorder_MeanAbsoluteDeviation | 0.91658045 |
| lbp-3D-m2_firstorder_Median | 0.87594941 |
| lbp-3D-m2_firstorder_Range | 0.79757962 |
| lbp-3D-m2_firstorder_RobustMeanAbsoluteDeviation | 0.94000196 |
| lbp-3D-m2_firstorder_RootMeanSquared | 0.89111907 |
| lbp-3D-m2_firstorder_Skewness | 0.94371116 |
| lbp-3D-m2_firstorder_TotalEnergy | 0.98842736 |
| lbp-3D-m2_firstorder_Uniformity | #NAME? |
| lbp-3D-m2_firstorder_Variance | 0.89667034 |
| lbp-3D-m2_glcm_Autocorrelation | #NAME? |
| lbp-3D-m2_glcm_Correlation | #NAME? |
| lbp-3D-m2_glcm_Id | #NAME? |
| lbp-3D-m2_glcm_Idm | #NAME? |
| lbp-3D-m2_glcm_Idmn | #NAME? |
| lbp-3D-m2_glcm_Idn | #NAME? |
| lbp-3D-m2_glcm_JointAverage | #NAME? |
| lbp-3D-m2_glcm_JointEnergy | #NAME? |
| lbp-3D-m2_glcm_MCC | #NAME? |
| lbp-3D-m2_glcm_MaximumProbability | #NAME? |
| lbp-3D-m2_glcm_SumAverage | #NAME? |
| lbp-3D-m2_gldm_DependenceEntropy | 0.92658835 |
| lbp-3D-m2_gldm_DependenceNonUniformity | 0.98133386 |
| lbp-3D-m2_gldm_DependenceNonUniformityNormalized | 0.9392569 |
| lbp-3D-m2_gldm_DependenceVariance | 0.94536875 |
| lbp-3D-m2_gldm_GrayLevelNonUniformity | 0.98652295 |
| lbp-3D-m2_gldm_HighGrayLevelEmphasis | #NAME? |
| lbp-3D-m2_gldm_LargeDependenceEmphasis | 0.98452133 |
| lbp-3D-m2_gldm_LargeDependenceHighGrayLevelEmphasis | 0.98452133 |
| lbp-3D-m2_gldm_LargeDependenceLowGrayLevelEmphasis | 0.98452133 |
| lbp-3D-m2_gldm_LowGrayLevelEmphasis | #NAME? |
| lbp-3D-m2_gldm_SmallDependenceEmphasis | 0.95307307 |
| lbp-3D-m2_gldm_SmallDependenceHighGrayLevelEmphasis | 0.95307307 |
| lbp-3D-m2_gldm_SmallDependenceLowGrayLevelEmphasis | 0.95307307 |
| lbp-3D-m2_glrlm_GrayLevelNonUniformity | 0.99010031 |
| lbp-3D-m2_glrlm_GrayLevelNonUniformityNormalized | #NAME? |
| lbp-3D-m2_glrlm_HighGrayLevelRunEmphasis | #NAME? |
| lbp-3D-m2_glrlm_LongRunEmphasis | 0.98320851 |
| lbp-3D-m2_glrlm_LongRunHighGrayLevelEmphasis | 0.98320851 |
| lbp-3D-m2_glrlm_LongRunLowGrayLevelEmphasis | 0.98320851 |
| lbp-3D-m2_glrlm_LowGrayLevelRunEmphasis | #NAME? |
| lbp-3D-m2_glrlm_RunEntropy | 0.99305285 |
| lbp-3D-m2_glrlm_RunLengthNonUniformity | 0.98410975 |
| lbp-3D-m2_glrlm_RunLengthNonUniformityNormalized | 0.97209315 |
| lbp-3D-m2_glrlm_RunPercentage | 0.98012294 |
| lbp-3D-m2_glrlm_RunVariance | 0.97350107 |
| lbp-3D-m2_glrlm_ShortRunEmphasis | 0.95456885 |
| lbp-3D-m2_glrlm_ShortRunHighGrayLevelEmphasis | 0.95456885 |
| lbp-3D-m2_glrlm_ShortRunLowGrayLevelEmphasis | 0.95456885 |
| lbp-3D-m2_glszm_GrayLevelNonUniformity | 0.95206612 |
| lbp-3D-m2_glszm_GrayLevelNonUniformityNormalized | #NAME? |
| lbp-3D-m2_glszm_HighGrayLevelZoneEmphasis | #NAME? |
| lbp-3D-m2_glszm_LargeAreaEmphasis | 0.93662402 |
| lbp-3D-m2_glszm_LargeAreaHighGrayLevelEmphasis | 0.93662402 |
| lbp-3D-m2_glszm_LargeAreaLowGrayLevelEmphasis | 0.93662402 |
| lbp-3D-m2_glszm_LowGrayLevelZoneEmphasis | #NAME? |
| lbp-3D-m2_glszm_SizeZoneNonUniformity | #NAME? |
| lbp-3D-m2_glszm_SizeZoneNonUniformityNormalized | 0.99325111 |
| lbp-3D-m2_glszm_SmallAreaEmphasis | 0.77578763 |
| lbp-3D-m2_glszm_SmallAreaHighGrayLevelEmphasis | 0.77578763 |
| lbp-3D-m2_glszm_SmallAreaLowGrayLevelEmphasis | 0.77578763 |
| lbp-3D-m2_glszm_ZonePercentage | 0.90379519 |
| lbp-3D-m2_ngtdm_Coarseness | #NAME? |
| original_firstorder_10Percentile | 0.99550332 |
| original_firstorder_90Percentile | 0.99387004 |
| original_firstorder_Energy | 0.98992124 |
| original_firstorder_Entropy | 0.98263692 |
| original_firstorder_InterquartileRange | 0.97016582 |
| original_firstorder_Kurtosis | 0.90137403 |
| original_firstorder_Maximum | 0.98881213 |
| original_firstorder_Mean | 0.99629825 |
| original_firstorder_MeanAbsoluteDeviation | 0.98088143 |
| original_firstorder_Median | 0.99671256 |
| original_firstorder_Minimum | 0.98259905 |
| original_firstorder_Range | 0.96987657 |
| original_firstorder_RobustMeanAbsoluteDeviation | 0.97184986 |
| original_firstorder_RootMeanSquared | 0.99574774 |
| original_firstorder_Skewness | 0.97077892 |
| original_firstorder_TotalEnergy | 0.99034623 |
| original_firstorder_Uniformity | 0.97404012 |
| original_firstorder_Variance | 0.9883709 |
| original_glcm_Autocorrelation | 0.96325948 |
| original_glcm_ClusterProminence | 0.98774897 |
| original_glcm_ClusterShade | 0.98056762 |
| original_glcm_ClusterTendency | 0.99053058 |
| original_glcm_Contrast | 0.96732572 |
| original_glcm_Correlation | 0.97182707 |
| original_glcm_DifferenceAverage | 0.97030261 |
| original_glcm_DifferenceEntropy | 0.98633219 |
| original_glcm_DifferenceVariance | 0.98634657 |
| original_glcm_Id | 0.97304152 |
| original_glcm_Idm | 0.97283503 |
| original_glcm_Idmn | 0.94704409 |
| original_glcm_Idn | 0.95088508 |
| original_glcm_Imc1 | 0.91594408 |
| original_glcm_Imc2 | 0.9813171 |
| original_glcm_InverseVariance | 0.97579676 |
| original_glcm_JointAverage | 0.96670099 |
| original_glcm_JointEnergy | 0.97898986 |
| original_glcm_JointEntropy | 0.98836326 |
| original_glcm_MCC | 0.94071398 |
| original_glcm_MaximumProbability | 0.96529428 |
| original_glcm_SumAverage | 0.96670099 |
| original_glcm_SumEntropy | 0.98833969 |
| original_glcm_SumSquares | 0.98682177 |
| original_gldm_DependenceEntropy | 0.9888223 |
| original_gldm_DependenceNonUniformity | 0.97746114 |
| original_gldm_DependenceNonUniformityNormalized | 0.9577549 |
| original_gldm_DependenceVariance | 0.9895274 |
| original_gldm_GrayLevelNonUniformity | 0.99852748 |
| original_gldm_GrayLevelVariance | 0.98841212 |
| original_gldm_HighGrayLevelEmphasis | 0.96503371 |
| original_gldm_LargeDependenceEmphasis | 0.98985422 |
| original_gldm_LargeDependenceHighGrayLevelEmphasis | 0.86549286 |
| original_gldm_LargeDependenceLowGrayLevelEmphasis | 0.99676219 |
| original_gldm_LowGrayLevelEmphasis | 0.9824868 |
| original_gldm_SmallDependenceEmphasis | 0.94485105 |
| original_gldm_SmallDependenceHighGrayLevelEmphasis | 0.98292288 |
| original_gldm_SmallDependenceLowGrayLevelEmphasis | 0.89855793 |
| original_glrlm_GrayLevelNonUniformity | 0.99729314 |
| original_glrlm_GrayLevelNonUniformityNormalized | 0.97309058 |
| original_glrlm_GrayLevelVariance | 0.98892589 |
| original_glrlm_HighGrayLevelRunEmphasis | 0.96479452 |
| original_glrlm_LongRunEmphasis | 0.98677792 |
| original_glrlm_LongRunHighGrayLevelEmphasis | 0.91619724 |
| original_glrlm_LongRunLowGrayLevelEmphasis | 0.99422641 |
| original_glrlm_LowGrayLevelRunEmphasis | 0.97957189 |
| original_glrlm_RunEntropy | 0.98466181 |
| original_glrlm_RunLengthNonUniformity | 0.97652962 |
| original_glrlm_RunLengthNonUniformityNormalized | 0.97637342 |
| original_glrlm_RunPercentage | 0.98135745 |
| original_glrlm_RunVariance | 0.98857871 |
| original_glrlm_ShortRunEmphasis | 0.978783 |
| original_glrlm_ShortRunHighGrayLevelEmphasis | 0.96834799 |
| original_glrlm_ShortRunLowGrayLevelEmphasis | 0.96713853 |
| original_glszm_GrayLevelNonUniformity | 0.96789145 |
| original_glszm_GrayLevelNonUniformityNormalized | 0.92352126 |
| original_glszm_GrayLevelVariance | 0.99029624 |
| original_glszm_HighGrayLevelZoneEmphasis | 0.96419415 |
| original_glszm_LargeAreaEmphasis | 0.99397915 |
| original_glszm_LargeAreaHighGrayLevelEmphasis | 0.86806802 |
| original_glszm_LargeAreaLowGrayLevelEmphasis | 0.9979347 |
| original_glszm_LowGrayLevelZoneEmphasis | 0.90650881 |
| original_glszm_SizeZoneNonUniformity | 0.89959519 |
| original_glszm_SizeZoneNonUniformityNormalized | 0.91987922 |
| original_glszm_SmallAreaEmphasis | 0.88910177 |
| original_glszm_SmallAreaHighGrayLevelEmphasis | 0.97428522 |
| original_glszm_SmallAreaLowGrayLevelEmphasis | 0.8730761 |
| original_glszm_ZoneEntropy | 0.95119011 |
| original_glszm_ZonePercentage | 0.94361649 |
| original_glszm_ZoneVariance | 0.99398409 |
| original_ngtdm_Busyness | 0.99296526 |
| original_ngtdm_Coarseness | 0.91376647 |
| original_ngtdm_Complexity | 0.96975131 |
| original_ngtdm_Contrast | 0.93225865 |
| original_ngtdm_Strength | 0.97168725 |
| original_shape_Elongation | 0.96396929 |
| original_shape_Flatness | 0.98052474 |
| original_shape_LeastAxisLength | 0.99399099 |
| original_shape_MajorAxisLength | 0.99556485 |
| original_shape_Maximum2DDiameterColumn | 0.98176385 |
| original_shape_Maximum2DDiameterRow | 0.99349459 |
| original_shape_Maximum2DDiameterSlice | 0.96115703 |
| original_shape_Maximum3DDiameter | 0.9944424 |
| original_shape_MeshVolume | 0.98709911 |
| original_shape_MinorAxisLength | 0.9802601 |
| original_shape_Sphericity | 0.98232949 |
| original_shape_SurfaceArea | 0.99250747 |
| original_shape_SurfaceVolumeRatio | 0.97208901 |
| original_shape_VoxelVolume | 0.9871762 |

1. **ICC_intra_ADC**

| parameters | Icc_value |
| --- | --- |
| lbp-3D-k_firstorder_10Percentile | 0.99515893 |
| lbp-3D-k_firstorder_90Percentile | 0.98698011 |
| lbp-3D-k_firstorder_Energy | 0.99640578 |
| lbp-3D-k_firstorder_Entropy | 0.98903472 |
| lbp-3D-k_firstorder_InterquartileRange | 0.97712055 |
| lbp-3D-k_firstorder_Kurtosis | 0.87564336 |
| lbp-3D-k_firstorder_Maximum | 0.96883143 |
| lbp-3D-k_firstorder_Mean | 0.99302881 |
| lbp-3D-k_firstorder_MeanAbsoluteDeviation | 0.98253094 |
| lbp-3D-k_firstorder_Median | 0.99273357 |
| lbp-3D-k_firstorder_Minimum | 0.95357573 |
| lbp-3D-k_firstorder_Range | 0.97032694 |
| lbp-3D-k_firstorder_RobustMeanAbsoluteDeviation | 0.97946774 |
| lbp-3D-k_firstorder_RootMeanSquared | 0.9947833 |
| lbp-3D-k_firstorder_Skewness | 0.92401627 |
| lbp-3D-k_firstorder_TotalEnergy | 0.99587512 |
| lbp-3D-k_firstorder_Uniformity | 0.98931936 |
| lbp-3D-k_firstorder_Variance | 0.98039231 |
| lbp-3D-k_glcm_Autocorrelation | 0.98671184 |
| lbp-3D-k_glcm_ClusterProminence | 0.97565836 |
| lbp-3D-k_glcm_ClusterShade | 0.97759766 |
| lbp-3D-k_glcm_ClusterTendency | 0.98515238 |
| lbp-3D-k_glcm_Contrast | 0.98765706 |
| lbp-3D-k_glcm_Correlation | 0.90520672 |
| lbp-3D-k_glcm_DifferenceAverage | 0.98765706 |
| lbp-3D-k_glcm_DifferenceEntropy | 0.98692966 |
| lbp-3D-k_glcm_DifferenceVariance | 0.98720505 |
| lbp-3D-k_glcm_Id | 0.98765706 |
| lbp-3D-k_glcm_Idm | 0.98765706 |
| lbp-3D-k_glcm_Idmn | 0.98765706 |
| lbp-3D-k_glcm_Idn | 0.98765706 |
| lbp-3D-k_glcm_Imc1 | 0.96489786 |
| lbp-3D-k_glcm_Imc2 | 0.88459111 |
| lbp-3D-k_glcm_InverseVariance | 0.98765706 |
| lbp-3D-k_glcm_JointAverage | 0.98712813 |
| lbp-3D-k_glcm_JointEnergy | 0.98705486 |
| lbp-3D-k_glcm_JointEntropy | 0.98653291 |
| lbp-3D-k_glcm_MCC | 0.91205168 |
| lbp-3D-k_glcm_MaximumProbability | 0.98748965 |
| lbp-3D-k_glcm_SumAverage | 0.98712813 |
| lbp-3D-k_glcm_SumEntropy | 0.98601361 |
| lbp-3D-k_glcm_SumSquares | 0.98676757 |
| lbp-3D-k_gldm_DependenceEntropy | 0.92801889 |
| lbp-3D-k_gldm_DependenceNonUniformity | 0.99598681 |
| lbp-3D-k_gldm_DependenceNonUniformityNormalized | 0.885588 |
| lbp-3D-k_gldm_DependenceVariance | 0.93003225 |
| lbp-3D-k_gldm_GrayLevelNonUniformity | 0.99644414 |
| lbp-3D-k_gldm_GrayLevelVariance | 0.98931936 |
| lbp-3D-k_gldm_HighGrayLevelEmphasis | 0.98952633 |
| lbp-3D-k_gldm_LargeDependenceEmphasis | 0.94460337 |
| lbp-3D-k_gldm_LargeDependenceHighGrayLevelEmphasis | 0.94718277 |
| lbp-3D-k_gldm_LargeDependenceLowGrayLevelEmphasis | 0.94398603 |
| lbp-3D-k_gldm_LowGrayLevelEmphasis | 0.98952633 |
| lbp-3D-k_gldm_SmallDependenceEmphasis | 0.94507037 |
| lbp-3D-k_gldm_SmallDependenceHighGrayLevelEmphasis | 0.9535792 |
| lbp-3D-k_gldm_SmallDependenceLowGrayLevelEmphasis | 0.91105897 |
| lbp-3D-k_glrlm_GrayLevelNonUniformity | 0.9956816 |
| lbp-3D-k_glrlm_GrayLevelNonUniformityNormalized | 0.985897 |
| lbp-3D-k_glrlm_GrayLevelVariance | 0.985897 |
| lbp-3D-k_glrlm_HighGrayLevelRunEmphasis | 0.98788522 |
| lbp-3D-k_glrlm_LongRunEmphasis | 0.9858476 |
| lbp-3D-k_glrlm_LongRunHighGrayLevelEmphasis | 0.98616188 |
| lbp-3D-k_glrlm_LongRunLowGrayLevelEmphasis | 0.98577128 |
| lbp-3D-k_glrlm_LowGrayLevelRunEmphasis | 0.98788522 |
| lbp-3D-k_glrlm_RunEntropy | 0.96267656 |
| lbp-3D-k_glrlm_RunLengthNonUniformity | 0.99183906 |
| lbp-3D-k_glrlm_RunLengthNonUniformityNormalized | 0.856152 |
| lbp-3D-k_glrlm_RunPercentage | 0.92905947 |
| lbp-3D-k_glrlm_RunVariance | 0.98893341 |
| lbp-3D-k_glrlm_ShortRunEmphasis | 0.8465152 |
| lbp-3D-k_glrlm_ShortRunHighGrayLevelEmphasis | 0.98588492 |
| lbp-3D-k_glrlm_ShortRunLowGrayLevelEmphasis | 0.85432763 |
| lbp-3D-k_glszm_GrayLevelNonUniformity | 0.99380687 |
| lbp-3D-k_glszm_GrayLevelNonUniformityNormalized | 0.86782483 |
| lbp-3D-k_glszm_GrayLevelVariance | 0.86782483 |
| lbp-3D-k_glszm_HighGrayLevelZoneEmphasis | 0.85230029 |
| lbp-3D-k_glszm_LargeAreaEmphasis | 0.99671271 |
| lbp-3D-k_glszm_LargeAreaHighGrayLevelEmphasis | 0.99669523 |
| lbp-3D-k_glszm_LargeAreaLowGrayLevelEmphasis | 0.99671585 |
| lbp-3D-k_glszm_LowGrayLevelZoneEmphasis | 0.85230029 |
| lbp-3D-k_glszm_SizeZoneNonUniformity | 0.98241257 |
| lbp-3D-k_glszm_SizeZoneNonUniformityNormalized | 0.79989335 |
| lbp-3D-k_glszm_SmallAreaEmphasis | 0.81710179 |
| lbp-3D-k_glszm_SmallAreaHighGrayLevelEmphasis | 0.82522949 |
| lbp-3D-k_glszm_SmallAreaLowGrayLevelEmphasis | 0.71270177 |
| lbp-3D-k_glszm_ZoneEntropy | 0.87977869 |
| lbp-3D-k_glszm_ZonePercentage | 0.95884545 |
| lbp-3D-k_glszm_ZoneVariance | 0.99673008 |
| lbp-3D-k_ngtdm_Busyness | 0.99502893 |
| lbp-3D-k_ngtdm_Coarseness | 0.95792345 |
| lbp-3D-k_ngtdm_Complexity | 0.99015507 |
| lbp-3D-k_ngtdm_Contrast | 0.99048676 |
| lbp-3D-k_ngtdm_Strength | 0.958057 |
| lbp-3D-m1_firstorder_10Percentile | 0.981461 |
| lbp-3D-m1_firstorder_90Percentile | 0.92139448 |
| lbp-3D-m1_firstorder_Energy | 0.99646157 |
| lbp-3D-m1_firstorder_Entropy | -3.68E-15 |
| lbp-3D-m1_firstorder_InterquartileRange | -0.3931084 |
| lbp-3D-m1_firstorder_Kurtosis | -9.50E-13 |
| lbp-3D-m1_firstorder_Maximum | 5.75E-15 |
| lbp-3D-m1_firstorder_Mean | -0.4263326 |
| lbp-3D-m1_firstorder_MeanAbsoluteDeviation | -0.2418195 |
| lbp-3D-m1_firstorder_Median | -0.0095569 |
| lbp-3D-m1_firstorder_Range | 0.02785429 |
| lbp-3D-m1_firstorder_RobustMeanAbsoluteDeviation | -0.2261666 |
| lbp-3D-m1_firstorder_RootMeanSquared | -0.3786096 |
| lbp-3D-m1_firstorder_Skewness | 4.19E-10 |
| lbp-3D-m1_firstorder_TotalEnergy | 8.30E-16 |
| lbp-3D-m1_firstorder_Uniformity | 6.89E-15 |
| lbp-3D-m1_firstorder_Variance | -3.51E-16 |
| lbp-3D-m1_glcm_Autocorrelation | #NAME? |
| lbp-3D-m1_glcm_Correlation | #NAME? |
| lbp-3D-m1_glcm_Id | #NAME? |
| lbp-3D-m1_glcm_Idm | #NAME? |
| lbp-3D-m1_glcm_Idmn | #NAME? |
| lbp-3D-m1_glcm_Idn | #NAME? |
| lbp-3D-m1_glcm_JointAverage | #NAME? |
| lbp-3D-m1_glcm_JointEnergy | #NAME? |
| lbp-3D-m1_glcm_MCC | #NAME? |
| lbp-3D-m1_glcm_MaximumProbability | #NAME? |
| lbp-3D-m1_glcm_SumAverage | -2.08E-15 |
| lbp-3D-m1_gldm_DependenceEntropy | -0.0003306 |
| lbp-3D-m1_gldm_DependenceNonUniformity | 6.23E-05 |
| lbp-3D-m1_gldm_DependenceNonUniformityNormalized | -0.0097612 |
| lbp-3D-m1_gldm_DependenceVariance | -0.0003911 |
| lbp-3D-m1_gldm_GrayLevelNonUniformity | 4.40E-16 |
| lbp-3D-m1_gldm_HighGrayLevelEmphasis | 3.99E-16 |
| lbp-3D-m1_gldm_LargeDependenceEmphasis | 0.95706733 |
| lbp-3D-m1_gldm_LargeDependenceHighGrayLevelEmphasis | 0.95706733 |
| lbp-3D-m1_gldm_LargeDependenceLowGrayLevelEmphasis | 2.80E-15 |
| lbp-3D-m1_gldm_LowGrayLevelEmphasis | 1.34E-11 |
| lbp-3D-m1_gldm_SmallDependenceEmphasis | 0.89085541 |
| lbp-3D-m1_gldm_SmallDependenceHighGrayLevelEmphasis | 0.89085541 |
| lbp-3D-m1_gldm_SmallDependenceLowGrayLevelEmphasis | -1.99E-06 |
| lbp-3D-m1_glrlm_GrayLevelNonUniformity | -2.27E-15 |
| lbp-3D-m1_glrlm_GrayLevelNonUniformityNormalized | #NAME? |
| lbp-3D-m1_glrlm_HighGrayLevelRunEmphasis | 2.92E-16 |
| lbp-3D-m1_glrlm_LongRunEmphasis | 0.99325952 |
| lbp-3D-m1_glrlm_LongRunHighGrayLevelEmphasis | 0.99325952 |
| lbp-3D-m1_glrlm_LongRunLowGrayLevelEmphasis | 8.09E-16 |
| lbp-3D-m1_glrlm_LowGrayLevelRunEmphasis | 3.48E-15 |
| lbp-3D-m1_glrlm_RunEntropy | 0.0051646 |
| lbp-3D-m1_glrlm_RunLengthNonUniformity | -0.0005534 |
| lbp-3D-m1_glrlm_RunLengthNonUniformityNormalized | 0.89242306 |
| lbp-3D-m1_glrlm_RunPercentage | -0.0061256 |
| lbp-3D-m1_glrlm_RunVariance | -0.0066934 |
| lbp-3D-m1_glrlm_ShortRunEmphasis | 0.85441725 |
| lbp-3D-m1_glrlm_ShortRunHighGrayLevelEmphasis | 0.85441725 |
| lbp-3D-m1_glrlm_ShortRunLowGrayLevelEmphasis | -0.122989 |
| lbp-3D-m1_glszm_GrayLevelNonUniformity | #NAME? |
| lbp-3D-m1_glszm_GrayLevelNonUniformityNormalized | #NAME? |
| lbp-3D-m1_glszm_HighGrayLevelZoneEmphasis | 9.93E-16 |
| lbp-3D-m1_glszm_LargeAreaEmphasis | 0.99226957 |
| lbp-3D-m1_glszm_LargeAreaHighGrayLevelEmphasis | 0.99226957 |
| lbp-3D-m1_glszm_LargeAreaLowGrayLevelEmphasis | 0 |
| lbp-3D-m1_glszm_LowGrayLevelZoneEmphasis | #NAME? |
| lbp-3D-m1_glszm_SizeZoneNonUniformity | 3.18E-16 |
| lbp-3D-m1_glszm_SizeZoneNonUniformityNormalized | -2.35E-05 |
| lbp-3D-m1_glszm_SmallAreaEmphasis | 0.16363673 |
| lbp-3D-m1_glszm_SmallAreaHighGrayLevelEmphasis | 0.16363673 |
| lbp-3D-m1_glszm_SmallAreaLowGrayLevelEmphasis | 0.00297346 |
| lbp-3D-m1_glszm_ZonePercentage | #NAME? |
| lbp-3D-m1_ngtdm_Coarseness | -1.14E-06 |
| lbp-3D-m2_firstorder_10Percentile | 0.35614877 |
| lbp-3D-m2_firstorder_90Percentile | 2.40E-07 |
| lbp-3D-m2_firstorder_Energy | -2.82E-07 |
| lbp-3D-m2_firstorder_InterquartileRange | -0.7863705 |
| lbp-3D-m2_firstorder_Kurtosis | -0.0646326 |
| lbp-3D-m2_firstorder_Maximum | 0.02584543 |
| lbp-3D-m2_firstorder_Mean | -0.6872272 |
| lbp-3D-m2_firstorder_MeanAbsoluteDeviation | -0.6110896 |
| lbp-3D-m2_firstorder_Median | 0.01078917 |
| lbp-3D-m2_firstorder_Range | 0.06615597 |
| lbp-3D-m2_firstorder_RobustMeanAbsoluteDeviation | -0.8224262 |
| lbp-3D-m2_firstorder_RootMeanSquared | -0.6473152 |
| lbp-3D-m2_firstorder_Skewness | -1.38E-08 |
| lbp-3D-m2_firstorder_TotalEnergy | 5.20E-16 |
| lbp-3D-m2_firstorder_Uniformity | 5.48E-16 |
| lbp-3D-m2_firstorder_Variance | 6.84E-15 |
| lbp-3D-m2_glcm_Autocorrelation | #NAME? |
| lbp-3D-m2_glcm_Correlation | #NAME? |
| lbp-3D-m2_glcm_Id | #NAME? |
| lbp-3D-m2_glcm_Idm | #NAME? |
| lbp-3D-m2_glcm_Idmn | #NAME? |
| lbp-3D-m2_glcm_Idn | #NAME? |
| lbp-3D-m2_glcm_JointAverage | #NAME? |
| lbp-3D-m2_glcm_JointEnergy | #NAME? |
| lbp-3D-m2_glcm_MCC | #NAME? |
| lbp-3D-m2_glcm_MaximumProbability | #NAME? |
| lbp-3D-m2_glcm_SumAverage | -2.08E-15 |
| lbp-3D-m2_gldm_DependenceEntropy | -0.0003306 |
| lbp-3D-m2_gldm_DependenceNonUniformity | 6.23E-05 |
| lbp-3D-m2_gldm_DependenceNonUniformityNormalized | -0.0097612 |
| lbp-3D-m2_gldm_DependenceVariance | -0.0003911 |
| lbp-3D-m2_gldm_GrayLevelNonUniformity | 4.40E-16 |
| lbp-3D-m2_gldm_HighGrayLevelEmphasis | 3.99E-16 |
| lbp-3D-m2_gldm_LargeDependenceEmphasis | 0.95706733 |
| lbp-3D-m2_gldm_LargeDependenceHighGrayLevelEmphasis | 0.95706733 |
| lbp-3D-m2_gldm_LargeDependenceLowGrayLevelEmphasis | 2.80E-15 |
| lbp-3D-m2_gldm_LowGrayLevelEmphasis | 1.34E-11 |
| lbp-3D-m2_gldm_SmallDependenceEmphasis | 0.89085541 |
| lbp-3D-m2_gldm_SmallDependenceHighGrayLevelEmphasis | 0.89085541 |
| lbp-3D-m2_gldm_SmallDependenceLowGrayLevelEmphasis | -1.99E-06 |
| lbp-3D-m2_glrlm_GrayLevelNonUniformity | -2.27E-15 |
| lbp-3D-m2_glrlm_GrayLevelNonUniformityNormalized | #NAME? |
| lbp-3D-m2_glrlm_HighGrayLevelRunEmphasis | #NAME? |
| lbp-3D-m2_glrlm_LongRunEmphasis | 0.99325952 |
| lbp-3D-m2_glrlm_LongRunHighGrayLevelEmphasis | 0.99325952 |
| lbp-3D-m2_glrlm_LongRunLowGrayLevelEmphasis | 0.99325952 |
| lbp-3D-m2_glrlm_LowGrayLevelRunEmphasis | #NAME? |
| lbp-3D-m2_glrlm_RunEntropy | 0.97327968 |
| lbp-3D-m2_glrlm_RunLengthNonUniformity | 0.95860765 |
| lbp-3D-m2_glrlm_RunLengthNonUniformityNormalized | 0.92410015 |
| lbp-3D-m2_glrlm_RunPercentage | 0.94725213 |
| lbp-3D-m2_glrlm_RunVariance | 0.99145117 |
| lbp-3D-m2_glrlm_ShortRunEmphasis | 0.85441725 |
| lbp-3D-m2_glrlm_ShortRunHighGrayLevelEmphasis | 0.85441725 |
| lbp-3D-m2_glrlm_ShortRunLowGrayLevelEmphasis | 0.85441725 |
| lbp-3D-m2_glszm_GrayLevelNonUniformity | -1.06E-16 |
| lbp-3D-m2_glszm_GrayLevelNonUniformityNormalized | #NAME? |
| lbp-3D-m2_glszm_HighGrayLevelZoneEmphasis | #NAME? |
| lbp-3D-m2_glszm_LargeAreaEmphasis | 0.99226957 |
| lbp-3D-m2_glszm_LargeAreaHighGrayLevelEmphasis | 0.99226957 |
| lbp-3D-m2_glszm_LargeAreaLowGrayLevelEmphasis | 0.99226957 |
| lbp-3D-m2_glszm_LowGrayLevelZoneEmphasis | #NAME? |
| lbp-3D-m2_glszm_SizeZoneNonUniformity | #NAME? |
| lbp-3D-m2_glszm_SizeZoneNonUniformityNormalized | 3.18E-16 |
| lbp-3D-m2_glszm_SmallAreaEmphasis | 0.16363673 |
| lbp-3D-m2_glszm_SmallAreaHighGrayLevelEmphasis | 0.16363673 |
| lbp-3D-m2_glszm_SmallAreaLowGrayLevelEmphasis | 0.16363673 |
| lbp-3D-m2_glszm_ZonePercentage | 0.92665349 |
| lbp-3D-m2_ngtdm_Coarseness | #NAME? |
| original_firstorder_10Percentile | 0.99909283 |
| original_firstorder_90Percentile | 0.99751072 |
| original_firstorder_Energy | 0.99411208 |
| original_firstorder_Entropy | 0.99472232 |
| original_firstorder_InterquartileRange | 0.98692372 |
| original_firstorder_Kurtosis | 0.91284807 |
| original_firstorder_Maximum | 0.97128841 |
| original_firstorder_Mean | 0.99917781 |
| original_firstorder_MeanAbsoluteDeviation | 0.9868531 |
| original_firstorder_Median | 0.99941806 |
| original_firstorder_Minimum | 0.99071834 |
| original_firstorder_Range | 0.94839751 |
| original_firstorder_RobustMeanAbsoluteDeviation | 0.98537323 |
| original_firstorder_RootMeanSquared | 0.99894584 |
| original_firstorder_Skewness | 0.96492872 |
| original_firstorder_TotalEnergy | 0.99347963 |
| original_firstorder_Uniformity | 0.99662515 |
| original_firstorder_Variance | 0.97559903 |
| original_glcm_Autocorrelation | 0.97890149 |
| original_glcm_ClusterProminence | 0.96944407 |
| original_glcm_ClusterShade | 0.97353018 |
| original_glcm_ClusterTendency | 0.9806752 |
| original_glcm_Contrast | 0.97569164 |
| original_glcm_Correlation | 0.96906948 |
| original_glcm_DifferenceAverage | 0.99028213 |
| original_glcm_DifferenceEntropy | 0.99349405 |
| original_glcm_DifferenceVariance | 0.96735299 |
| original_glcm_Id | 0.99600392 |
| original_glcm_Idm | 0.99611191 |
| original_glcm_Idmn | 0.95394725 |
| original_glcm_Idn | 0.9496098 |
| original_glcm_Imc1 | 0.96975991 |
| original_glcm_Imc2 | 0.99192747 |
| original_glcm_InverseVariance | 0.99636101 |
| original_glcm_JointAverage | 0.97447134 |
| original_glcm_JointEnergy | 0.9921949 |
| original_glcm_JointEntropy | 0.99083891 |
| original_glcm_MCC | 0.8430473 |
| original_glcm_MaximumProbability | 0.99244939 |
| original_glcm_SumAverage | 0.97447134 |
| original_glcm_SumEntropy | 0.99474306 |
| original_glcm_SumSquares | 0.98005546 |
| original_gldm_DependenceEntropy | 0.98925665 |
| original_gldm_DependenceNonUniformity | 0.99472263 |
| original_gldm_DependenceNonUniformityNormalized | 0.99462476 |
| original_gldm_DependenceVariance | 0.99895631 |
| original_gldm_GrayLevelNonUniformity | 0.99885693 |
| original_gldm_GrayLevelVariance | 0.97560659 |
| original_gldm_HighGrayLevelEmphasis | 0.9804992 |
| original_gldm_LargeDependenceEmphasis | 0.99852448 |
| original_gldm_LargeDependenceHighGrayLevelEmphasis | 0.93066654 |
| original_gldm_LargeDependenceLowGrayLevelEmphasis | 0.85786271 |
| original_gldm_LowGrayLevelEmphasis | 0.76986813 |
| original_gldm_SmallDependenceEmphasis | 0.99374644 |
| original_gldm_SmallDependenceHighGrayLevelEmphasis | 0.98077463 |
| original_gldm_SmallDependenceLowGrayLevelEmphasis | 0.84117321 |
| original_glrlm_GrayLevelNonUniformity | 0.99864044 |
| original_glrlm_GrayLevelNonUniformityNormalized | 0.99638839 |
| original_glrlm_GrayLevelVariance | 0.97605658 |
| original_glrlm_HighGrayLevelRunEmphasis | 0.98065596 |
| original_glrlm_LongRunEmphasis | 0.99815434 |
| original_glrlm_LongRunHighGrayLevelEmphasis | 0.9780875 |
| original_glrlm_LongRunLowGrayLevelEmphasis | 0.78216633 |
| original_glrlm_LowGrayLevelRunEmphasis | 0.7758531 |
| original_glrlm_RunEntropy | 0.99324953 |
| original_glrlm_RunLengthNonUniformity | 0.99581462 |
| original_glrlm_RunLengthNonUniformityNormalized | 0.99764191 |
| original_glrlm_RunPercentage | 0.99794348 |
| original_glrlm_RunVariance | 0.99834332 |
| original_glrlm_ShortRunEmphasis | 0.99774183 |
| original_glrlm_ShortRunHighGrayLevelEmphasis | 0.98105352 |
| original_glrlm_ShortRunLowGrayLevelEmphasis | 0.77741015 |
| original_glszm_GrayLevelNonUniformity | 0.99549123 |
| original_glszm_GrayLevelNonUniformityNormalized | 0.99208996 |
| original_glszm_GrayLevelVariance | 0.97851074 |
| original_glszm_HighGrayLevelZoneEmphasis | 0.98236245 |
| original_glszm_LargeAreaEmphasis | 0.99880914 |
| original_glszm_LargeAreaHighGrayLevelEmphasis | 0.88140368 |
| original_glszm_LargeAreaLowGrayLevelEmphasis | 0.98944588 |
| original_glszm_LowGrayLevelZoneEmphasis | 0.79833791 |
| original_glszm_SizeZoneNonUniformity | 0.99227366 |
| original_glszm_SizeZoneNonUniformityNormalized | 0.98050742 |
| original_glszm_SmallAreaEmphasis | 0.9798969 |
| original_glszm_SmallAreaHighGrayLevelEmphasis | 0.98185933 |
| original_glszm_SmallAreaLowGrayLevelEmphasis | 0.82090605 |
| original_glszm_ZoneEntropy | 0.98689044 |
| original_glszm_ZonePercentage | 0.99505905 |
| original_glszm_ZoneVariance | 0.99882742 |
| original_ngtdm_Busyness | 0.96881973 |
| original_ngtdm_Coarseness | 0.9437946 |
| original_ngtdm_Complexity | 0.9739369 |
| original_ngtdm_Contrast | 0.9748993 |
| original_ngtdm_Strength | 0.93156008 |
| original_shape_Elongation | 0.96231175 |
| original_shape_Flatness | 0.93739085 |
| original_shape_LeastAxisLength | 0.99046354 |
| original_shape_MajorAxisLength | 0.99444703 |
| original_shape_Maximum2DDiameterColumn | 0.98660794 |
| original_shape_Maximum2DDiameterRow | 0.99708645 |
| original_shape_Maximum2DDiameterSlice | 0.97986254 |
| original_shape_Maximum3DDiameter | 0.99640573 |
| original_shape_MeshVolume | 0.99581487 |
| original_shape_MinorAxisLength | 0.99532727 |
| original_shape_Sphericity | 0.78103196 |
| original_shape_SurfaceArea | 0.9967778 |
| original_shape_SurfaceVolumeRatio | 0.97075477 |
| original_shape_VoxelVolume | 0.9958492 |

1. **ICC_tre_T_2_WI**

| parameters | Icc_value |
| --- | --- |
| lbp-3D-k_firstorder_10Percentile | 0.99258041 |
| lbp-3D-k_firstorder_90Percentile | 0.98327972 |
| lbp-3D-k_firstorder_Energy | 0.99603746 |
| lbp-3D-k_firstorder_Entropy | 0.98316709 |
| lbp-3D-k_firstorder_InterquartileRange | 0.97718237 |
| lbp-3D-k_firstorder_Kurtosis | 0.98982683 |
| lbp-3D-k_firstorder_Maximum | 0.98464265 |
| lbp-3D-k_firstorder_Mean | 0.99190654 |
| lbp-3D-k_firstorder_MeanAbsoluteDeviation | 0.9841097 |
| lbp-3D-k_firstorder_Median | 0.99117589 |
| lbp-3D-k_firstorder_Minimum | 0.96018701 |
| lbp-3D-k_firstorder_Range | 0.98500834 |
| lbp-3D-k_firstorder_RobustMeanAbsoluteDeviation | 0.97738762 |
| lbp-3D-k_firstorder_RootMeanSquared | 0.99390525 |
| lbp-3D-k_firstorder_Skewness | 0.98626756 |
| lbp-3D-k_firstorder_TotalEnergy | 0.99536924 |
| lbp-3D-k_firstorder_Uniformity | 0.98501 |
| lbp-3D-k_firstorder_Variance | 0.992566 |
| lbp-3D-k_glcm_Autocorrelation | 0.98277595 |
| lbp-3D-k_glcm_ClusterProminence | 0.97811038 |
| lbp-3D-k_glcm_ClusterShade | 0.9777425 |
| lbp-3D-k_glcm_ClusterTendency | 0.98133804 |
| lbp-3D-k_glcm_Contrast | 0.98280916 |
| lbp-3D-k_glcm_DifferenceAverage | 0.98280916 |
| lbp-3D-k_glcm_DifferenceEntropy | 0.98005892 |
| lbp-3D-k_glcm_DifferenceVariance | 0.98145755 |
| lbp-3D-k_glcm_Id | 0.98280916 |
| lbp-3D-k_glcm_Idm | 0.98280916 |
| lbp-3D-k_glcm_Idmn | 0.98280916 |
| lbp-3D-k_glcm_Idn | 0.98280916 |
| lbp-3D-k_glcm_Imc2 | 0.93012363 |
| lbp-3D-k_glcm_InverseVariance | 0.98280916 |
| lbp-3D-k_glcm_JointAverage | 0.98279626 |
| lbp-3D-k_glcm_JointEnergy | 0.98181724 |
| lbp-3D-k_glcm_JointEntropy | 0.98102472 |
| lbp-3D-k_glcm_MaximumProbability | 0.98281058 |
| lbp-3D-k_glcm_SumAverage | 0.98279626 |
| lbp-3D-k_glcm_SumEntropy | 0.98047555 |
| lbp-3D-k_glcm_SumSquares | 0.98206739 |
| lbp-3D-k_gldm_DependenceEntropy | 0.96645796 |
| lbp-3D-k_gldm_DependenceNonUniformity | 0.99395833 |
| lbp-3D-k_gldm_DependenceNonUniformityNormalized | 0.94822275 |
| lbp-3D-k_gldm_DependenceVariance | 0.95048247 |
| lbp-3D-k_gldm_GrayLevelNonUniformity | 0.99615228 |
| lbp-3D-k_gldm_GrayLevelVariance | 0.98501 |
| lbp-3D-k_gldm_HighGrayLevelEmphasis | 0.98567341 |
| lbp-3D-k_gldm_LargeDependenceEmphasis | 0.98341453 |
| lbp-3D-k_gldm_LargeDependenceHighGrayLevelEmphasis | 0.98401811 |
| lbp-3D-k_gldm_LargeDependenceLowGrayLevelEmphasis | 0.98326714 |
| lbp-3D-k_gldm_LowGrayLevelEmphasis | 0.98567341 |
| lbp-3D-k_gldm_SmallDependenceEmphasis | 0.95426452 |
| lbp-3D-k_gldm_SmallDependenceHighGrayLevelEmphasis | 0.95364389 |
| lbp-3D-k_gldm_SmallDependenceLowGrayLevelEmphasis | 0.95621241 |
| lbp-3D-k_glrlm_GrayLevelNonUniformity | 0.99633885 |
| lbp-3D-k_glrlm_GrayLevelNonUniformityNormalized | 0.98551484 |
| lbp-3D-k_glrlm_GrayLevelVariance | 0.98551484 |
| lbp-3D-k_glrlm_HighGrayLevelRunEmphasis | 0.98866333 |
| lbp-3D-k_glrlm_LongRunEmphasis | 0.9728373 |
| lbp-3D-k_glrlm_LongRunHighGrayLevelEmphasis | 0.97336588 |
| lbp-3D-k_glrlm_LongRunLowGrayLevelEmphasis | 0.97270694 |
| lbp-3D-k_glrlm_LowGrayLevelRunEmphasis | 0.98866333 |
| lbp-3D-k_glrlm_RunEntropy | 0.99487058 |
| lbp-3D-k_glrlm_RunLengthNonUniformity | 0.99409923 |
| lbp-3D-k_glrlm_RunLengthNonUniformityNormalized | 0.97995413 |
| lbp-3D-k_glrlm_RunPercentage | 0.97917301 |
| lbp-3D-k_glrlm_RunVariance | 0.98157394 |
| lbp-3D-k_glrlm_ShortRunEmphasis | 0.9014614 |
| lbp-3D-k_glrlm_ShortRunHighGrayLevelEmphasis | 0.96836311 |
| lbp-3D-k_glrlm_ShortRunLowGrayLevelEmphasis | 0.93225152 |
| lbp-3D-k_glszm_GrayLevelNonUniformity | 0.99626079 |
| lbp-3D-k_glszm_LargeAreaEmphasis | 0.99306644 |
| lbp-3D-k_glszm_LargeAreaHighGrayLevelEmphasis | 0.99306539 |
| lbp-3D-k_glszm_LargeAreaLowGrayLevelEmphasis | 0.99306672 |
| lbp-3D-k_glszm_SizeZoneNonUniformity | 0.99393547 |
| lbp-3D-k_glszm_ZonePercentage | 0.95886133 |
| lbp-3D-k_glszm_ZoneVariance | 0.9938952 |
| lbp-3D-k_ngtdm_Busyness | 0.99689754 |
| lbp-3D-k_ngtdm_Complexity | 0.98578205 |
| lbp-3D-k_ngtdm_Contrast | 0.98827292 |
| lbp-3D-m1_firstorder_10Percentile | 0.82124539 |
| lbp-3D-m1_firstorder_90Percentile | 0.97277484 |
| lbp-3D-m1_firstorder_Energy | 0.99629337 |
| lbp-3D-m1_firstorder_InterquartileRange | 0.96816346 |
| lbp-3D-m1_firstorder_Kurtosis | 0.96272315 |
| lbp-3D-m1_firstorder_Mean | 0.96049716 |
| lbp-3D-m1_firstorder_MeanAbsoluteDeviation | 0.98993547 |
| lbp-3D-m1_firstorder_Median | 0.85877649 |
| lbp-3D-m1_firstorder_RobustMeanAbsoluteDeviation | 0.9847162 |
| lbp-3D-m1_firstorder_RootMeanSquared | 0.9679168 |
| lbp-3D-m1_firstorder_Skewness | 0.91913884 |
| lbp-3D-m1_firstorder_TotalEnergy | 0.99555834 |
| lbp-3D-m1_firstorder_Variance | 0.98194399 |
| lbp-3D-m1_gldm_DependenceEntropy | 0.95562023 |
| lbp-3D-m1_gldm_DependenceNonUniformity | 0.99401984 |
| lbp-3D-m1_gldm_DependenceNonUniformityNormalized | 0.93227934 |
| lbp-3D-m1_gldm_GrayLevelNonUniformity | 0.99624109 |
| lbp-3D-m1_gldm_LargeDependenceEmphasis | 0.98792917 |
| lbp-3D-m1_gldm_LargeDependenceHighGrayLevelEmphasis | 0.98792917 |
| lbp-3D-m1_gldm_LargeDependenceLowGrayLevelEmphasis | 0.98792917 |
| lbp-3D-m1_gldm_SmallDependenceEmphasis | 0.96203608 |
| lbp-3D-m1_gldm_SmallDependenceHighGrayLevelEmphasis | 0.96203608 |
| lbp-3D-m1_gldm_SmallDependenceLowGrayLevelEmphasis | 0.96203608 |
| lbp-3D-m1_glrlm_GrayLevelNonUniformity | 0.99597724 |
| lbp-3D-m1_glrlm_LongRunEmphasis | 0.99270906 |
| lbp-3D-m1_glrlm_LongRunHighGrayLevelEmphasis | 0.99270906 |
| lbp-3D-m1_glrlm_LongRunLowGrayLevelEmphasis | 0.99270906 |
| lbp-3D-m1_glrlm_RunEntropy | 0.99610245 |
| lbp-3D-m1_glrlm_RunLengthNonUniformity | 0.98030714 |
| lbp-3D-m1_glrlm_RunLengthNonUniformityNormalized | 0.98311216 |
| lbp-3D-m1_glrlm_RunPercentage | 0.98475782 |
| lbp-3D-m1_glrlm_RunVariance | 0.9923389 |
| lbp-3D-m1_glrlm_ShortRunEmphasis | 0.94621467 |
| lbp-3D-m1_glrlm_ShortRunHighGrayLevelEmphasis | 0.94621467 |
| lbp-3D-m1_glrlm_ShortRunLowGrayLevelEmphasis | 0.94621467 |
| lbp-3D-m1_glszm_LargeAreaEmphasis | 0.98669694 |
| lbp-3D-m1_glszm_LargeAreaHighGrayLevelEmphasis | 0.98669694 |
| lbp-3D-m1_glszm_LargeAreaLowGrayLevelEmphasis | 0.98669694 |
| lbp-3D-m1_glszm_SmallAreaEmphasis | 0.98043908 |
| lbp-3D-m1_glszm_SmallAreaHighGrayLevelEmphasis | 0.98043908 |
| lbp-3D-m1_glszm_SmallAreaLowGrayLevelEmphasis | 0.98043908 |
| lbp-3D-m1_glszm_ZonePercentage | 0.99077468 |
| lbp-3D-m2_firstorder_10Percentile | 0.87554495 |
| lbp-3D-m2_firstorder_90Percentile | 0.96744154 |
| lbp-3D-m2_firstorder_Energy | 0.9963763 |
| lbp-3D-m2_firstorder_InterquartileRange | 0.95623844 |
| lbp-3D-m2_firstorder_Kurtosis | 0.85714371 |
| lbp-3D-m2_firstorder_Mean | 0.92393784 |
| lbp-3D-m2_firstorder_MeanAbsoluteDeviation | 0.95378561 |
| lbp-3D-m2_firstorder_Median | 0.88053227 |
| lbp-3D-m2_firstorder_RobustMeanAbsoluteDeviation | 0.95718213 |
| lbp-3D-m2_firstorder_RootMeanSquared | 0.92092974 |
| lbp-3D-m2_firstorder_Skewness | 0.91536226 |
| lbp-3D-m2_firstorder_TotalEnergy | 0.99568526 |
| lbp-3D-m2_firstorder_Variance | 0.96222191 |
| lbp-3D-m2_gldm_DependenceEntropy | 0.95562023 |
| lbp-3D-m2_gldm_DependenceNonUniformity | 0.99401984 |
| lbp-3D-m2_gldm_DependenceNonUniformityNormalized | 0.93227934 |
| lbp-3D-m2_gldm_GrayLevelNonUniformity | 0.99624109 |
| lbp-3D-m2_gldm_LargeDependenceEmphasis | 0.98792917 |
| lbp-3D-m2_gldm_LargeDependenceHighGrayLevelEmphasis | 0.98792917 |
| lbp-3D-m2_gldm_LargeDependenceLowGrayLevelEmphasis | 0.98792917 |
| lbp-3D-m2_gldm_SmallDependenceEmphasis | 0.96203608 |
| lbp-3D-m2_gldm_SmallDependenceHighGrayLevelEmphasis | 0.96203608 |
| lbp-3D-m2_gldm_SmallDependenceLowGrayLevelEmphasis | 0.96203608 |
| lbp-3D-m2_glrlm_GrayLevelNonUniformity | 0.99597724 |
| lbp-3D-m2_glrlm_LongRunEmphasis | 0.99270906 |
| lbp-3D-m2_glrlm_LongRunHighGrayLevelEmphasis | 0.99270906 |
| lbp-3D-m2_glrlm_LongRunLowGrayLevelEmphasis | 0.99270906 |
| lbp-3D-m2_glrlm_RunEntropy | 0.99610245 |
| lbp-3D-m2_glrlm_RunLengthNonUniformity | 0.98030714 |
| lbp-3D-m2_glrlm_RunLengthNonUniformityNormalized | 0.98311216 |
| lbp-3D-m2_glrlm_RunPercentage | 0.98475782 |
| lbp-3D-m2_glrlm_RunVariance | 0.9923389 |
| lbp-3D-m2_glrlm_ShortRunEmphasis | 0.94621467 |
| lbp-3D-m2_glrlm_ShortRunHighGrayLevelEmphasis | 0.94621467 |
| lbp-3D-m2_glrlm_ShortRunLowGrayLevelEmphasis | 0.94621467 |
| lbp-3D-m2_glszm_LargeAreaEmphasis | 0.98669694 |
| lbp-3D-m2_glszm_LargeAreaHighGrayLevelEmphasis | 0.98669694 |
| lbp-3D-m2_glszm_LargeAreaLowGrayLevelEmphasis | 0.98669694 |
| lbp-3D-m2_glszm_SmallAreaEmphasis | 0.98043908 |
| lbp-3D-m2_glszm_SmallAreaHighGrayLevelEmphasis | 0.98043908 |
| lbp-3D-m2_glszm_SmallAreaLowGrayLevelEmphasis | 0.98043908 |
| lbp-3D-m2_glszm_ZonePercentage | 0.99077468 |
| original_firstorder_10Percentile | 0.99660737 |
| original_firstorder_90Percentile | 0.99691936 |
| original_firstorder_Energy | 0.99688711 |
| original_firstorder_Entropy | 0.99767831 |
| original_firstorder_InterquartileRange | 0.98375853 |
| original_firstorder_Kurtosis | 0.93534967 |
| original_firstorder_Maximum | 0.99711775 |
| original_firstorder_Mean | 0.99843208 |
| original_firstorder_MeanAbsoluteDeviation | 0.99267323 |
| original_firstorder_Median | 0.99827974 |
| original_firstorder_Minimum | 0.9865632 |
| original_firstorder_Range | 0.99036711 |
| original_firstorder_RobustMeanAbsoluteDeviation | 0.98230823 |
| original_firstorder_RootMeanSquared | 0.99854507 |
| original_firstorder_Skewness | 0.97396347 |
| original_firstorder_TotalEnergy | 0.99679795 |
| original_firstorder_Uniformity | 0.9980838 |
| original_firstorder_Variance | 0.99208813 |
| original_glcm_Autocorrelation | 0.96727913 |
| original_glcm_ClusterProminence | 0.99756186 |
| original_glcm_ClusterShade | 0.99814754 |
| original_glcm_ClusterTendency | 0.99143631 |
| original_glcm_Contrast | 0.9633526 |
| original_glcm_Correlation | 0.98254713 |
| original_glcm_DifferenceAverage | 0.98188563 |
| original_glcm_DifferenceEntropy | 0.99509434 |
| original_glcm_DifferenceVariance | 0.97540205 |
| original_glcm_Id | 0.99640966 |
| original_glcm_Idm | 0.99697075 |
| original_glcm_Idmn | 0.85245639 |
| original_glcm_Idn | 0.9217159 |
| original_glcm_Imc1 | 0.99186108 |
| original_glcm_Imc2 | 0.99492961 |
| original_glcm_InverseVariance | 0.99375349 |
| original_glcm_JointAverage | 0.95936355 |
| original_glcm_JointEnergy | 0.99859009 |
| original_glcm_JointEntropy | 0.99737912 |
| original_glcm_MCC | 0.94609477 |
| original_glcm_MaximumProbability | 0.99591562 |
| original_glcm_SumAverage | 0.95936355 |
| original_glcm_SumEntropy | 0.9972806 |
| original_glcm_SumSquares | 0.98676238 |
| original_gldm_DependenceEntropy | 0.9970115 |
| original_gldm_DependenceNonUniformity | 0.99482702 |
| original_gldm_DependenceNonUniformityNormalized | 0.99547789 |
| original_gldm_DependenceVariance | 0.99606125 |
| original_gldm_GrayLevelNonUniformity | 0.99824155 |
| original_gldm_GrayLevelVariance | 0.99207958 |
| original_gldm_HighGrayLevelEmphasis | 0.9689471 |
| original_gldm_LargeDependenceEmphasis | 0.9977703 |
| original_gldm_LargeDependenceHighGrayLevelEmphasis | 0.9086131 |
| original_gldm_LargeDependenceLowGrayLevelEmphasis | 0.9940495 |
| original_gldm_LowGrayLevelEmphasis | 0.97513535 |
| original_gldm_SmallDependenceEmphasis | 0.99492384 |
| original_gldm_SmallDependenceHighGrayLevelEmphasis | 0.9894843 |
| original_glrlm_GrayLevelNonUniformity | 0.99781145 |
| original_glrlm_GrayLevelNonUniformityNormalized | 0.99792837 |
| original_glrlm_GrayLevelVariance | 0.99302999 |
| original_glrlm_HighGrayLevelRunEmphasis | 0.96969587 |
| original_glrlm_LongRunEmphasis | 0.99631805 |
| original_glrlm_LongRunHighGrayLevelEmphasis | 0.92334639 |
| original_glrlm_LongRunLowGrayLevelEmphasis | 0.99445178 |
| original_glrlm_LowGrayLevelRunEmphasis | 0.9748453 |
| original_glrlm_RunEntropy | 0.99728548 |
| original_glrlm_RunLengthNonUniformity | 0.99500994 |
| original_glrlm_RunLengthNonUniformityNormalized | 0.99721529 |
| original_glrlm_RunPercentage | 0.99742303 |
| original_glrlm_RunVariance | 0.99568158 |
| original_glrlm_ShortRunEmphasis | 0.9972185 |
| original_glrlm_ShortRunHighGrayLevelEmphasis | 0.9734911 |
| original_glrlm_ShortRunLowGrayLevelEmphasis | 0.96281032 |
| original_glszm_GrayLevelNonUniformity | 0.98957408 |
| original_glszm_GrayLevelNonUniformityNormalized | 0.99619023 |
| original_glszm_GrayLevelVariance | 0.99716311 |
| original_glszm_HighGrayLevelZoneEmphasis | 0.97746656 |
| original_glszm_LargeAreaEmphasis | 0.99627057 |
| original_glszm_LargeAreaHighGrayLevelEmphasis | 0.94835107 |
| original_glszm_LargeAreaLowGrayLevelEmphasis | 0.99171596 |
| original_glszm_LowGrayLevelZoneEmphasis | 0.96346452 |
| original_glszm_SizeZoneNonUniformity | 0.98729731 |
| original_glszm_SizeZoneNonUniformityNormalized | 0.98620852 |
| original_glszm_SmallAreaEmphasis | 0.98309154 |
| original_glszm_SmallAreaHighGrayLevelEmphasis | 0.98413721 |
| original_glszm_SmallAreaLowGrayLevelEmphasis | 0.90432923 |
| original_glszm_ZoneEntropy | 0.99061651 |
| original_glszm_ZonePercentage | 0.99479965 |
| original_glszm_ZoneVariance | 0.99623861 |
| original_ngtdm_Busyness | 0.97069616 |
| original_ngtdm_Coarseness | 0.98756172 |
| original_ngtdm_Complexity | 0.99320087 |
| original_ngtdm_Contrast | 0.90884777 |
| original_ngtdm_Strength | 0.98787572 |
| original_shape_Elongation | 0.94216589 |
| original_shape_Flatness | 0.96402766 |
| original_shape_LeastAxisLength | 0.99422632 |
| original_shape_MajorAxisLength | 0.99500373 |
| original_shape_Maximum2DDiameterColumn | 0.99190852 |
| original_shape_Maximum2DDiameterRow | 0.99735232 |
| original_shape_Maximum2DDiameterSlice | 0.98997289 |
| original_shape_Maximum3DDiameter | 0.99700454 |
| original_shape_MeshVolume | 0.99549153 |
| original_shape_MinorAxisLength | 0.99058991 |
| original_shape_SurfaceArea | 0.99667828 |
| original_shape_SurfaceVolumeRatio | 0.99039393 |
| original_shape_VoxelVolume | 0.99549089 |

1. **ICC_tre_DWI**

| parameters | Icc_value |
| --- | --- |
| lbp-3D-k_firstorder_10Percentile | 0.98481243 |
| lbp-3D-k_firstorder_90Percentile | 0.91676658 |
| lbp-3D-k_firstorder_Energy | 0.99658669 |
| lbp-3D-k_firstorder_Entropy | 0.89338156 |
| lbp-3D-k_firstorder_InterquartileRange | 0.97728514 |
| lbp-3D-k_firstorder_Kurtosis | 0.9861269 |
| lbp-3D-k_firstorder_Maximum | 0.98083044 |
| lbp-3D-k_firstorder_Mean | 0.9647239 |
| lbp-3D-k_firstorder_MeanAbsoluteDeviation | 0.93178344 |
| lbp-3D-k_firstorder_Median | 0.97944473 |
| lbp-3D-k_firstorder_Range | 0.98225158 |
| lbp-3D-k_firstorder_RobustMeanAbsoluteDeviation | 0.95847433 |
| lbp-3D-k_firstorder_RootMeanSquared | 0.9922507 |
| lbp-3D-k_firstorder_Skewness | 0.9814753 |
| lbp-3D-k_firstorder_TotalEnergy | 0.99658227 |
| lbp-3D-k_firstorder_Uniformity | 0.87636698 |
| lbp-3D-k_firstorder_Variance | 0.93646497 |
| lbp-3D-k_glcm_Contrast | 0.8563097 |
| lbp-3D-k_glcm_DifferenceAverage | 0.8563097 |
| lbp-3D-k_glcm_DifferenceEntropy | 0.8929747 |
| lbp-3D-k_glcm_DifferenceVariance | 0.88395509 |
| lbp-3D-k_glcm_Id | 0.85630971 |
| lbp-3D-k_glcm_Idm | 0.85630971 |
| lbp-3D-k_glcm_Idmn | 0.85630971 |
| lbp-3D-k_glcm_Idn | 0.8563097 |
| lbp-3D-k_glcm_InverseVariance | 0.8563097 |
| lbp-3D-k_glcm_JointEnergy | 0.85450499 |
| lbp-3D-k_glcm_MaximumProbability | 0.83002538 |
| lbp-3D-k_gldm_DependenceEntropy | 0.98143121 |
| lbp-3D-k_gldm_DependenceNonUniformity | 0.99705399 |
| lbp-3D-k_gldm_DependenceNonUniformityNormalized | 0.98743349 |
| lbp-3D-k_gldm_DependenceVariance | 0.94689051 |
| lbp-3D-k_gldm_GrayLevelNonUniformity | 0.99617025 |
| lbp-3D-k_gldm_GrayLevelVariance | 0.87636698 |
| lbp-3D-k_gldm_HighGrayLevelEmphasis | 0.85747917 |
| lbp-3D-k_gldm_LargeDependenceEmphasis | 0.9818219 |
| lbp-3D-k_gldm_LargeDependenceHighGrayLevelEmphasis | 0.98486415 |
| lbp-3D-k_gldm_LargeDependenceLowGrayLevelEmphasis | 0.98089531 |
| lbp-3D-k_gldm_LowGrayLevelEmphasis | 0.85747917 |
| lbp-3D-k_gldm_SmallDependenceEmphasis | 0.93456563 |
| lbp-3D-k_gldm_SmallDependenceHighGrayLevelEmphasis | 0.92074537 |
| lbp-3D-k_gldm_SmallDependenceLowGrayLevelEmphasis | 0.96343122 |
| lbp-3D-k_glrlm_GrayLevelNonUniformity | 0.99545209 |
| lbp-3D-k_glrlm_GrayLevelNonUniformityNormalized | 0.94650568 |
| lbp-3D-k_glrlm_GrayLevelVariance | 0.94650568 |
| lbp-3D-k_glrlm_HighGrayLevelRunEmphasis | 0.95077868 |
| lbp-3D-k_glrlm_LongRunEmphasis | 0.99262348 |
| lbp-3D-k_glrlm_LongRunHighGrayLevelEmphasis | 0.99332318 |
| lbp-3D-k_glrlm_LongRunLowGrayLevelEmphasis | 0.99243391 |
| lbp-3D-k_glrlm_LowGrayLevelRunEmphasis | 0.95077868 |
| lbp-3D-k_glrlm_RunEntropy | 0.99330588 |
| lbp-3D-k_glrlm_RunLengthNonUniformity | 0.99231429 |
| lbp-3D-k_glrlm_RunLengthNonUniformityNormalized | 0.97509748 |
| lbp-3D-k_glrlm_RunPercentage | 0.98141341 |
| lbp-3D-k_glrlm_RunVariance | 0.99521099 |
| lbp-3D-k_glrlm_ShortRunEmphasis | 0.96726888 |
| lbp-3D-k_glrlm_ShortRunHighGrayLevelEmphasis | 0.9455315 |
| lbp-3D-k_glrlm_ShortRunLowGrayLevelEmphasis | 0.97959277 |
| lbp-3D-k_glszm_GrayLevelNonUniformity | 0.99076196 |
| lbp-3D-k_glszm_LargeAreaEmphasis | 0.99740454 |
| lbp-3D-k_glszm_LargeAreaHighGrayLevelEmphasis | 0.99742177 |
| lbp-3D-k_glszm_LargeAreaLowGrayLevelEmphasis | 0.99740011 |
| lbp-3D-k_glszm_SizeZoneNonUniformity | 0.96893686 |
| lbp-3D-k_glszm_ZonePercentage | 0.89470464 |
| lbp-3D-k_glszm_ZoneVariance | 0.99752196 |
| lbp-3D-k_ngtdm_Busyness | 0.99719041 |
| lbp-3D-k_ngtdm_Complexity | 0.90346851 |
| lbp-3D-k_ngtdm_Contrast | 0.80932698 |
| lbp-3D-m1_firstorder_90Percentile | 0.92394185 |
| lbp-3D-m1_firstorder_Energy | 0.99644322 |
| lbp-3D-m1_firstorder_InterquartileRange | 0.9671837 |
| lbp-3D-m1_firstorder_Kurtosis | 0.95339259 |
| lbp-3D-m1_firstorder_Mean | 0.94380088 |
| lbp-3D-m1_firstorder_MeanAbsoluteDeviation | 0.96252064 |
| lbp-3D-m1_firstorder_Median | 0.91766483 |
| lbp-3D-m1_firstorder_RobustMeanAbsoluteDeviation | 0.96619356 |
| lbp-3D-m1_firstorder_RootMeanSquared | 0.94926541 |
| lbp-3D-m1_firstorder_Skewness | 0.94532968 |
| lbp-3D-m1_firstorder_TotalEnergy | 0.99649294 |
| lbp-3D-m1_firstorder_Variance | 0.94632847 |
| lbp-3D-m1_gldm_DependenceEntropy | 0.943007 |
| lbp-3D-m1_gldm_DependenceNonUniformity | 0.9965489 |
| lbp-3D-m1_gldm_DependenceNonUniformityNormalized | 0.96085434 |
| lbp-3D-m1_gldm_DependenceVariance | 0.95504414 |
| lbp-3D-m1_gldm_GrayLevelNonUniformity | 0.99649561 |
| lbp-3D-m1_gldm_LargeDependenceEmphasis | 0.99103946 |
| lbp-3D-m1_gldm_LargeDependenceHighGrayLevelEmphasis | 0.99103946 |
| lbp-3D-m1_gldm_LargeDependenceLowGrayLevelEmphasis | 0.99103946 |
| lbp-3D-m1_gldm_SmallDependenceEmphasis | 0.97279558 |
| lbp-3D-m1_gldm_SmallDependenceHighGrayLevelEmphasis | 0.97279558 |
| lbp-3D-m1_gldm_SmallDependenceLowGrayLevelEmphasis | 0.97279558 |
| lbp-3D-m1_glrlm_GrayLevelNonUniformity | 0.99579151 |
| lbp-3D-m1_glrlm_LongRunEmphasis | 0.99609456 |
| lbp-3D-m1_glrlm_LongRunHighGrayLevelEmphasis | 0.99609456 |
| lbp-3D-m1_glrlm_LongRunLowGrayLevelEmphasis | 0.99609456 |
| lbp-3D-m1_glrlm_RunEntropy | 0.99393728 |
| lbp-3D-m1_glrlm_RunLengthNonUniformity | 0.98986042 |
| lbp-3D-m1_glrlm_RunLengthNonUniformityNormalized | 0.98591477 |
| lbp-3D-m1_glrlm_RunPercentage | 0.98967612 |
| lbp-3D-m1_glrlm_RunVariance | 0.99209615 |
| lbp-3D-m1_glrlm_ShortRunEmphasis | 0.97554696 |
| lbp-3D-m1_glrlm_ShortRunHighGrayLevelEmphasis | 0.97554696 |
| lbp-3D-m1_glrlm_ShortRunLowGrayLevelEmphasis | 0.97554696 |
| lbp-3D-m1_glszm_GrayLevelNonUniformity | 1 |
| lbp-3D-m1_glszm_LargeAreaEmphasis | 0.98341722 |
| lbp-3D-m1_glszm_LargeAreaHighGrayLevelEmphasis | 0.98341722 |
| lbp-3D-m1_glszm_LargeAreaLowGrayLevelEmphasis | 0.98341722 |
| lbp-3D-m1_glszm_SizeZoneNonUniformityNormalized | 1 |
| lbp-3D-m1_glszm_ZonePercentage | 0.94996982 |
| lbp-3D-m2_firstorder_Energy | 0.99649469 |
| lbp-3D-m2_firstorder_InterquartileRange | 0.93378238 |
| lbp-3D-m2_firstorder_Kurtosis | 0.92319146 |
| lbp-3D-m2_firstorder_MeanAbsoluteDeviation | 0.92309887 |
| lbp-3D-m2_firstorder_RobustMeanAbsoluteDeviation | 0.93937709 |
| lbp-3D-m2_firstorder_Skewness | 0.92466404 |
| lbp-3D-m2_firstorder_TotalEnergy | 0.99649175 |
| lbp-3D-m2_gldm_DependenceEntropy | 0.943007 |
| lbp-3D-m2_gldm_DependenceNonUniformity | 0.9965489 |
| lbp-3D-m2_gldm_DependenceNonUniformityNormalized | 0.96085434 |
| lbp-3D-m2_gldm_DependenceVariance | 0.95504414 |
| lbp-3D-m2_gldm_GrayLevelNonUniformity | 0.99649561 |
| lbp-3D-m2_gldm_LargeDependenceEmphasis | 0.99103946 |
| lbp-3D-m2_gldm_LargeDependenceHighGrayLevelEmphasis | 0.99103946 |
| lbp-3D-m2_gldm_LargeDependenceLowGrayLevelEmphasis | 0.99103946 |
| lbp-3D-m2_gldm_SmallDependenceEmphasis | 0.97279558 |
| lbp-3D-m2_gldm_SmallDependenceHighGrayLevelEmphasis | 0.97279558 |
| lbp-3D-m2_gldm_SmallDependenceLowGrayLevelEmphasis | 0.97279558 |
| lbp-3D-m2_glrlm_GrayLevelNonUniformity | 0.99579151 |
| lbp-3D-m2_glrlm_LongRunEmphasis | 0.99609456 |
| lbp-3D-m2_glrlm_LongRunHighGrayLevelEmphasis | 0.99609456 |
| lbp-3D-m2_glrlm_LongRunLowGrayLevelEmphasis | 0.99609456 |
| lbp-3D-m2_glrlm_RunEntropy | 0.99393728 |
| lbp-3D-m2_glrlm_RunLengthNonUniformity | 0.98986042 |
| lbp-3D-m2_glrlm_RunLengthNonUniformityNormalized | 0.98591477 |
| lbp-3D-m2_glrlm_RunPercentage | 0.98967612 |
| lbp-3D-m2_glrlm_RunVariance | 0.99209615 |
| lbp-3D-m2_glrlm_ShortRunEmphasis | 0.97554696 |
| lbp-3D-m2_glrlm_ShortRunHighGrayLevelEmphasis | 0.97554696 |
| lbp-3D-m2_glrlm_ShortRunLowGrayLevelEmphasis | 0.97554696 |
| lbp-3D-m2_glszm_GrayLevelNonUniformity | 1 |
| lbp-3D-m2_glszm_LargeAreaEmphasis | 0.98341722 |
| lbp-3D-m2_glszm_LargeAreaHighGrayLevelEmphasis | 0.98341722 |
| lbp-3D-m2_glszm_LargeAreaLowGrayLevelEmphasis | 0.98341722 |
| lbp-3D-m2_glszm_SizeZoneNonUniformityNormalized | 1 |
| lbp-3D-m2_glszm_ZonePercentage | 0.94996982 |
| original_firstorder_10Percentile | 0.99588081 |
| original_firstorder_90Percentile | 0.99438718 |
| original_firstorder_Energy | 0.99739677 |
| original_firstorder_Entropy | 0.98767592 |
| original_firstorder_InterquartileRange | 0.97209621 |
| original_firstorder_Kurtosis | 0.81439839 |
| original_firstorder_Maximum | 0.98692364 |
| original_firstorder_Mean | 0.99686195 |
| original_firstorder_MeanAbsoluteDeviation | 0.98261736 |
| original_firstorder_Median | 0.99767141 |
| original_firstorder_Minimum | 0.98355922 |
| original_firstorder_Range | 0.96743636 |
| original_firstorder_RobustMeanAbsoluteDeviation | 0.97373797 |
| original_firstorder_RootMeanSquared | 0.99645569 |
| original_firstorder_Skewness | 0.97471157 |
| original_firstorder_TotalEnergy | 0.99751679 |
| original_firstorder_Uniformity | 0.9811177 |
| original_firstorder_Variance | 0.98492722 |
| original_glcm_Autocorrelation | 0.94812136 |
| original_glcm_ClusterProminence | 0.96290355 |
| original_glcm_ClusterShade | 0.97488057 |
| original_glcm_ClusterTendency | 0.98140877 |
| original_glcm_Contrast | 0.97190673 |
| original_glcm_Correlation | 0.98046686 |
| original_glcm_DifferenceAverage | 0.98044529 |
| original_glcm_DifferenceEntropy | 0.98653463 |
| original_glcm_DifferenceVariance | 0.98201466 |
| original_glcm_Id | 0.98460414 |
| original_glcm_Idm | 0.98444898 |
| original_glcm_Idmn | 0.9737734 |
| original_glcm_Idn | 0.96960599 |
| original_glcm_Imc1 | 0.96444536 |
| original_glcm_Imc2 | 0.98989745 |
| original_glcm_InverseVariance | 0.98353187 |
| original_glcm_JointAverage | 0.96322314 |
| original_glcm_JointEnergy | 0.98215637 |
| original_glcm_JointEntropy | 0.98895316 |
| original_glcm_MCC | 0.9646924 |
| original_glcm_MaximumProbability | 0.97639543 |
| original_glcm_SumAverage | 0.96322314 |
| original_glcm_SumEntropy | 0.98886962 |
| original_glcm_SumSquares | 0.98167166 |
| original_gldm_DependenceEntropy | 0.98822487 |
| original_gldm_DependenceNonUniformity | 0.99363535 |
| original_gldm_DependenceNonUniformityNormalized | 0.99362822 |
| original_gldm_DependenceVariance | 0.99384018 |
| original_gldm_GrayLevelNonUniformity | 0.99921551 |
| original_gldm_GrayLevelVariance | 0.9846728 |
| original_gldm_HighGrayLevelEmphasis | 0.94558011 |
| original_gldm_LargeDependenceEmphasis | 0.99410406 |
| original_gldm_LargeDependenceLowGrayLevelEmphasis | 0.99656699 |
| original_gldm_LowGrayLevelEmphasis | 0.98132467 |
| original_gldm_SmallDependenceEmphasis | 0.981831 |
| original_gldm_SmallDependenceHighGrayLevelEmphasis | 0.96994793 |
| original_glrlm_GrayLevelNonUniformity | 0.99871294 |
| original_glrlm_GrayLevelNonUniformityNormalized | 0.9794039 |
| original_glrlm_GrayLevelVariance | 0.98543258 |
| original_glrlm_HighGrayLevelRunEmphasis | 0.94528528 |
| original_glrlm_LongRunEmphasis | 0.99041264 |
| original_glrlm_LongRunHighGrayLevelEmphasis | 0.85694201 |
| original_glrlm_LongRunLowGrayLevelEmphasis | 0.99448272 |
| original_glrlm_LowGrayLevelRunEmphasis | 0.97846975 |
| original_glrlm_RunEntropy | 0.98533043 |
| original_glrlm_RunLengthNonUniformity | 0.9937388 |
| original_glrlm_RunLengthNonUniformityNormalized | 0.9901578 |
| original_glrlm_RunPercentage | 0.99109271 |
| original_glrlm_RunVariance | 0.99123943 |
| original_glrlm_ShortRunEmphasis | 0.98963042 |
| original_glrlm_ShortRunHighGrayLevelEmphasis | 0.95228575 |
| original_glrlm_ShortRunLowGrayLevelEmphasis | 0.96386639 |
| original_glszm_GrayLevelNonUniformity | 0.99073772 |
| original_glszm_GrayLevelNonUniformityNormalized | 0.9216586 |
| original_glszm_GrayLevelVariance | 0.99105162 |
| original_glszm_HighGrayLevelZoneEmphasis | 0.944292 |
| original_glszm_LargeAreaEmphasis | 0.97579303 |
| original_glszm_LargeAreaLowGrayLevelEmphasis | 0.97011305 |
| original_glszm_LowGrayLevelZoneEmphasis | 0.91936594 |
| original_glszm_SizeZoneNonUniformityNormalized | 0.96125593 |
| original_glszm_SmallAreaHighGrayLevelEmphasis | 0.95532065 |
| original_glszm_ZoneEntropy | 0.96667785 |
| original_glszm_ZonePercentage | 0.98025734 |
| original_glszm_ZoneVariance | 0.97923776 |
| original_ngtdm_Busyness | 0.98660516 |
| original_ngtdm_Coarseness | 0.96551177 |
| original_ngtdm_Complexity | 0.97404287 |
| original_ngtdm_Contrast | 0.94257342 |
| original_ngtdm_Strength | 0.97764518 |
| original_shape_Elongation | 0.9633761 |
| original_shape_Flatness | 0.95661764 |
| original_shape_LeastAxisLength | 0.99365656 |
| original_shape_MajorAxisLength | 0.99581015 |
| original_shape_Maximum2DDiameterColumn | 0.98408044 |
| original_shape_Maximum2DDiameterRow | 0.99579517 |
| original_shape_Maximum2DDiameterSlice | 0.97427439 |
| original_shape_Maximum3DDiameter | 0.99668374 |
| original_shape_MeshVolume | 0.99650323 |
| original_shape_MinorAxisLength | 0.99017846 |
| original_shape_Sphericity | 0.97253648 |
| original_shape_SurfaceArea | 0.99685429 |
| original_shape_SurfaceVolumeRatio | 0.9811868 |
| original_shape_VoxelVolume | 0.99652878 |

1. **ICC_tre_ADC**

| parameters | Icc_value |
| --- | --- |
| lbp-3D-k_firstorder_10Percentile | 0.99745041 |
| lbp-3D-k_firstorder_90Percentile | 0.98981502 |
| lbp-3D-k_firstorder_Energy | 0.99715977 |
| lbp-3D-k_firstorder_Entropy | 0.99144695 |
| lbp-3D-k_firstorder_InterquartileRange | 0.97888367 |
| lbp-3D-k_firstorder_Maximum | 0.95387821 |
| lbp-3D-k_firstorder_Mean | 0.99489178 |
| lbp-3D-k_firstorder_MeanAbsoluteDeviation | 0.98874916 |
| lbp-3D-k_firstorder_Median | 0.99409844 |
| lbp-3D-k_firstorder_Minimum | 0.96509742 |
| lbp-3D-k_firstorder_Range | 0.95435553 |
| lbp-3D-k_firstorder_RobustMeanAbsoluteDeviation | 0.98340808 |
| lbp-3D-k_firstorder_RootMeanSquared | 0.99579055 |
| lbp-3D-k_firstorder_Skewness | 0.88247421 |
| lbp-3D-k_firstorder_TotalEnergy | 0.99731198 |
| lbp-3D-k_firstorder_Uniformity | 0.99160491 |
| lbp-3D-k_firstorder_Variance | 0.98752953 |
| lbp-3D-k_glcm_Autocorrelation | 0.99314066 |
| lbp-3D-k_glcm_ClusterProminence | 0.9879686 |
| lbp-3D-k_glcm_ClusterShade | 0.98912521 |
| lbp-3D-k_glcm_ClusterTendency | 0.99267968 |
| lbp-3D-k_glcm_Contrast | 0.99324872 |
| lbp-3D-k_glcm_Correlation | 0.92772487 |
| lbp-3D-k_glcm_DifferenceAverage | 0.99324872 |
| lbp-3D-k_glcm_DifferenceEntropy | 0.99326481 |
| lbp-3D-k_glcm_DifferenceVariance | 0.99332222 |
| lbp-3D-k_glcm_Id | 0.99324872 |
| lbp-3D-k_glcm_Idm | 0.99324872 |
| lbp-3D-k_glcm_Idmn | 0.99324872 |
| lbp-3D-k_glcm_Idn | 0.99324872 |
| lbp-3D-k_glcm_Imc1 | 0.96490953 |
| lbp-3D-k_glcm_InverseVariance | 0.99324872 |
| lbp-3D-k_glcm_JointAverage | 0.9932925 |
| lbp-3D-k_glcm_JointEnergy | 0.99335823 |
| lbp-3D-k_glcm_JointEntropy | 0.99323205 |
| lbp-3D-k_glcm_MCC | 0.92436632 |
| lbp-3D-k_glcm_MaximumProbability | 0.99336641 |
| lbp-3D-k_glcm_SumAverage | 0.9932925 |
| lbp-3D-k_glcm_SumEntropy | 0.99308083 |
| lbp-3D-k_glcm_SumSquares | 0.99326966 |
| lbp-3D-k_gldm_DependenceEntropy | 0.96148761 |
| lbp-3D-k_gldm_DependenceNonUniformity | 0.99507841 |
| lbp-3D-k_gldm_DependenceVariance | 0.94793152 |
| lbp-3D-k_gldm_GrayLevelNonUniformity | 0.99700732 |
| lbp-3D-k_gldm_GrayLevelVariance | 0.99160491 |
| lbp-3D-k_gldm_HighGrayLevelEmphasis | 0.99161822 |
| lbp-3D-k_gldm_LargeDependenceEmphasis | 0.98365731 |
| lbp-3D-k_gldm_LargeDependenceHighGrayLevelEmphasis | 0.98520268 |
| lbp-3D-k_gldm_LargeDependenceLowGrayLevelEmphasis | 0.98325752 |
| lbp-3D-k_gldm_LowGrayLevelEmphasis | 0.99161822 |
| lbp-3D-k_gldm_SmallDependenceEmphasis | 0.96576882 |
| lbp-3D-k_gldm_SmallDependenceHighGrayLevelEmphasis | 0.96951964 |
| lbp-3D-k_gldm_SmallDependenceLowGrayLevelEmphasis | 0.94917912 |
| lbp-3D-k_glrlm_GrayLevelNonUniformity | 0.99738505 |
| lbp-3D-k_glrlm_GrayLevelNonUniformityNormalized | 0.99491876 |
| lbp-3D-k_glrlm_GrayLevelVariance | 0.99491876 |
| lbp-3D-k_glrlm_HighGrayLevelRunEmphasis | 0.99492616 |
| lbp-3D-k_glrlm_LongRunEmphasis | 0.99121439 |
| lbp-3D-k_glrlm_LongRunHighGrayLevelEmphasis | 0.99169941 |
| lbp-3D-k_glrlm_LongRunLowGrayLevelEmphasis | 0.99109317 |
| lbp-3D-k_glrlm_LowGrayLevelRunEmphasis | 0.99492616 |
| lbp-3D-k_glrlm_RunEntropy | 0.99120082 |
| lbp-3D-k_glrlm_RunLengthNonUniformity | 0.99566617 |
| lbp-3D-k_glrlm_RunPercentage | 0.97520473 |
| lbp-3D-k_glrlm_RunVariance | 0.99375458 |
| lbp-3D-k_glrlm_ShortRunHighGrayLevelEmphasis | 0.98710046 |
| lbp-3D-k_glszm_GrayLevelNonUniformity | 0.99334511 |
| lbp-3D-k_glszm_LargeAreaEmphasis | 0.99493734 |
| lbp-3D-k_glszm_LargeAreaHighGrayLevelEmphasis | 0.99452272 |
| lbp-3D-k_glszm_LargeAreaLowGrayLevelEmphasis | 0.99503818 |
| lbp-3D-k_glszm_SizeZoneNonUniformity | 0.98676813 |
| lbp-3D-k_glszm_ZonePercentage | 0.97112888 |
| lbp-3D-k_glszm_ZoneVariance | 0.99496465 |
| lbp-3D-k_ngtdm_Busyness | 0.99413377 |
| lbp-3D-k_ngtdm_Coarseness | 0.98826397 |
| lbp-3D-k_ngtdm_Complexity | 0.99099582 |
| lbp-3D-k_ngtdm_Contrast | 0.99172431 |
| lbp-3D-k_ngtdm_Strength | 0.98835375 |
| lbp-3D-m1_firstorder_10Percentile | 0.97550694 |
| lbp-3D-m1_firstorder_90Percentile | 0.93369453 |
| lbp-3D-m1_firstorder_Energy | 0.99696011 |
| lbp-3D-m1_gldm_LargeDependenceEmphasis | 0.99047983 |
| lbp-3D-m1_gldm_LargeDependenceHighGrayLevelEmphasis | 0.99047983 |
| lbp-3D-m1_glrlm_LongRunEmphasis | 0.99438656 |
| lbp-3D-m1_glrlm_LongRunHighGrayLevelEmphasis | 0.99438656 |
| lbp-3D-m1_glszm_LargeAreaEmphasis | 0.98884643 |
| lbp-3D-m1_glszm_LargeAreaHighGrayLevelEmphasis | 0.98884643 |
| lbp-3D-m2_gldm_LargeDependenceEmphasis | 0.99047983 |
| lbp-3D-m2_gldm_LargeDependenceHighGrayLevelEmphasis | 0.99047983 |
| lbp-3D-m2_glrlm_LongRunEmphasis | 0.99438656 |
| lbp-3D-m2_glrlm_LongRunHighGrayLevelEmphasis | 0.99438656 |
| lbp-3D-m2_glrlm_LongRunLowGrayLevelEmphasis | 0.99438656 |
| lbp-3D-m2_glrlm_RunEntropy | 0.99502545 |
| lbp-3D-m2_glrlm_RunLengthNonUniformity | 0.97816671 |
| lbp-3D-m2_glrlm_RunLengthNonUniformityNormalized | 0.97388002 |
| lbp-3D-m2_glrlm_RunPercentage | 0.98463853 |
| lbp-3D-m2_glrlm_RunVariance | 0.99380283 |
| lbp-3D-m2_glszm_LargeAreaEmphasis | 0.98884643 |
| lbp-3D-m2_glszm_LargeAreaHighGrayLevelEmphasis | 0.98884643 |
| lbp-3D-m2_glszm_LargeAreaLowGrayLevelEmphasis | 0.98884643 |
| lbp-3D-m2_glszm_ZonePercentage | 0.99332092 |
| original_firstorder_10Percentile | 0.99950784 |
| original_firstorder_90Percentile | 0.99860257 |
| original_firstorder_Energy | 0.99230835 |
| original_firstorder_Entropy | 0.99647435 |
| original_firstorder_InterquartileRange | 0.99377731 |
| original_firstorder_Kurtosis | 0.90821982 |
| original_firstorder_Maximum | 0.96488386 |
| original_firstorder_Mean | 0.9994635 |
| original_firstorder_MeanAbsoluteDeviation | 0.99151636 |
| original_firstorder_Median | 0.99962622 |
| original_firstorder_Minimum | 0.98952474 |
| original_firstorder_Range | 0.93483342 |
| original_firstorder_RobustMeanAbsoluteDeviation | 0.99351347 |
| original_firstorder_RootMeanSquared | 0.99927463 |
| original_firstorder_Skewness | 0.9618252 |
| original_firstorder_TotalEnergy | 0.99260986 |
| original_firstorder_Uniformity | 0.99812002 |
| original_firstorder_Variance | 0.98074006 |
| original_glcm_Autocorrelation | 0.97954229 |
| original_glcm_ClusterProminence | 0.98010788 |
| original_glcm_ClusterShade | 0.9813569 |
| original_glcm_ClusterTendency | 0.98741386 |
| original_glcm_Contrast | 0.98352438 |
| original_glcm_Correlation | 0.96955407 |
| original_glcm_DifferenceAverage | 0.99384795 |
| original_glcm_DifferenceEntropy | 0.99647194 |
| original_glcm_DifferenceVariance | 0.97558061 |
| original_glcm_Id | 0.99822636 |
| original_glcm_Idm | 0.99840494 |
| original_glcm_Idmn | 0.95825403 |
| original_glcm_Idn | 0.95014618 |
| original_glcm_Imc1 | 0.98753299 |
| original_glcm_Imc2 | 0.99413791 |
| original_glcm_InverseVariance | 0.99832153 |
| original_glcm_JointAverage | 0.97163283 |
| original_glcm_JointEnergy | 0.99695247 |
| original_glcm_JointEntropy | 0.99699006 |
| original_glcm_MaximumProbability | 0.99771002 |
| original_glcm_SumAverage | 0.97163283 |
| original_glcm_SumEntropy | 0.99693977 |
| original_glcm_SumSquares | 0.98811372 |
| original_gldm_DependenceEntropy | 0.99508156 |
| original_gldm_DependenceNonUniformity | 0.99529247 |
| original_gldm_DependenceNonUniformityNormalized | 0.99646154 |
| original_gldm_DependenceVariance | 0.99922345 |
| original_gldm_GrayLevelNonUniformity | 0.99885411 |
| original_gldm_GrayLevelVariance | 0.98071652 |
| original_gldm_HighGrayLevelEmphasis | 0.98166419 |
| original_gldm_LargeDependenceEmphasis | 0.99897004 |
| original_gldm_LargeDependenceHighGrayLevelEmphasis | 0.92712338 |
| original_gldm_SmallDependenceEmphasis | 0.99636839 |
| original_gldm_SmallDependenceHighGrayLevelEmphasis | 0.98405146 |
| original_glrlm_GrayLevelNonUniformity | 0.99869722 |
| original_glrlm_GrayLevelNonUniformityNormalized | 0.99798029 |
| original_glrlm_GrayLevelVariance | 0.98059 |
| original_glrlm_HighGrayLevelRunEmphasis | 0.9818351 |
| original_glrlm_LongRunEmphasis | 0.99878904 |
| original_glrlm_LongRunHighGrayLevelEmphasis | 0.97860132 |
| original_glrlm_RunEntropy | 0.99559779 |
| original_glrlm_RunLengthNonUniformity | 0.99637531 |
| original_glrlm_RunLengthNonUniformityNormalized | 0.99850936 |
| original_glrlm_RunPercentage | 0.99872735 |
| original_glrlm_RunVariance | 0.99891307 |
| original_glrlm_ShortRunEmphasis | 0.99854702 |
| original_glrlm_ShortRunHighGrayLevelEmphasis | 0.98237224 |
| original_glszm_GrayLevelNonUniformity | 0.99720999 |
| original_glszm_GrayLevelNonUniformityNormalized | 0.99374715 |
| original_glszm_GrayLevelVariance | 0.97685513 |
| original_glszm_HighGrayLevelZoneEmphasis | 0.98368385 |
| original_glszm_LargeAreaEmphasis | 0.99951233 |
| original_glszm_LargeAreaLowGrayLevelEmphasis | 0.99043257 |
| original_glszm_SizeZoneNonUniformity | 0.99388815 |
| original_glszm_SizeZoneNonUniformityNormalized | 0.98525502 |
| original_glszm_SmallAreaEmphasis | 0.98534297 |
| original_glszm_SmallAreaHighGrayLevelEmphasis | 0.98383168 |
| original_glszm_ZoneEntropy | 0.99148274 |
| original_glszm_ZonePercentage | 0.99768718 |
| original_glszm_ZoneVariance | 0.99952276 |
| original_ngtdm_Busyness | 0.96568378 |
| original_ngtdm_Coarseness | 0.98486123 |
| original_ngtdm_Complexity | 0.95005029 |
| original_ngtdm_Contrast | 0.98304773 |
| original_ngtdm_Strength | 0.94388592 |
| original_shape_Elongation | 0.96703827 |
| original_shape_Flatness | 0.96104607 |
| original_shape_LeastAxisLength | 0.99666372 |
| original_shape_MajorAxisLength | 0.99217379 |
| original_shape_Maximum2DDiameterColumn | 0.99116051 |
| original_shape_Maximum2DDiameterRow | 0.99713 |
| original_shape_Maximum2DDiameterSlice | 0.98859649 |
| original_shape_Maximum3DDiameter | 0.99423563 |
| original_shape_MeshVolume | 0.99696802 |
| original_shape_MinorAxisLength | 0.99671082 |
| original_shape_SurfaceArea | 0.99815682 |
| original_shape_SurfaceVolumeRatio | 0.9933811 |
| original_shape_VoxelVolume | 0.99698763 |
